# Surveillance of Food and Waterborne Pathogens in North-East India: Protocol for a Laboratory-Based Sentinel Surveillance Study

**DOI:** 10.2196/56469

**Published:** 2024-10-21

**Authors:** Venencia Albert, Thandavarayan Ramamurthy, Madhuchhanda Das, Samaresh Das, Anup Kumar Ojha, Pallab Sarmah, Dimpu Gogoi, Karma G Dolma, Tapan Majumdar, Indira Sarangthem, Tapan Dutta, Suranjana Chaliha Hazarika

**Affiliations:** 1 Indian Council of Medical Research New Delhi India; 2 National Institute For Research in Bacterial Infections (NIRBI) Indian Council of Medical Research Kolkata India; 3 Centre for Development of Advanced Computing (C-DAC) Kolkata India; 4 Regional Medical Research Centre, Dibrugarh Indian Council of Medical Research Assam India; 5 Department of Microbiology Sikkim Manipal Institute of Medical Sciences Sikkim Manipal University Gangtok India; 6 Agartala Government Medical College Agartala, Tripura India; 7 Institute of Bioresources and Sustainable Development (IBSD) Imphal India; 8 College of Veterinary Sciences & Animal Husbandry Central Agricultural University Aizawl India; 9 Gauhati Medical College and Hospital Assam India

**Keywords:** foodborne diseases, enteric pathogens, surveillance, food safety, outbreak, antimicrobial resistance, public health

## Abstract

**Background:**

Food safety is a global concern, which is often underestimated owing to challenges in investigating foodborne diseases. These challenges arise from the increased globalization of the food trade, advancements in agricultural practices, and shifts in environmental factors. In North-East India, common diarrheal outbreaks from fermented foods pose significant health risks. Despite these challenges, systematic data on foodborne pathogens is lacking in India, highlighting a crucial gap in understanding food safety issues.

**Objective:**

The aim of this research protocol is to establish an integrated surveillance system to identify enteric pathogens circulating within humans, food animals, and the environment through a health approach in North-East India, and to conduct outbreak investigations.

**Methods:**

The Indian Council of Medical Research (ICMR) initiated a surveillance study across all 8 North-East states in India, employing a centralized digital database for data collation. The project aims to enhance the infrastructure for microbial culture, antibiotic sensitivity testing, and molecular epidemiological studies. The study involves laboratory-based surveillance of foodborne pathogens in market foods, hospitalized diarrheal patients, poultry and animal farms, slaughterhouses, butcher shops, and diarrheal outbreaks. A standardized case report form ensures consistent data collection of age, sex, signs, symptoms, and admission dates for diarrheal cases. Stool and rectal swabs will undergo testing for pathogen identification and antimicrobial resistance. Similarly, samples of market foods, food animals, and the environment will be collected. Outbreaks confirmed by the Integrated Disease Surveillance Project (IDSP) will be thoroughly investigated following standardized guidelines.

**Results:**

In phase I, 5 surveillance centers were established across 4 states (ie, Assam [Dibrugarh and Guwahati], Tripura, Sikkim, and Arunachal Pradesh) in 2020. Following an interim phase I data assessment and the successful establishment of a streamlined system for data procurement, investigation, recording, and analysis, along with the implementation of regular training and monitoring programs, phase II expansion was initiated in 2023-24. This includes the addition of 7 more centers (including 3 veterinary centers) in the remaining 4 states (ie, Manipur, Meghalaya, Mizoram, and Nagaland), eventually covering the entire North-Eastern Region of India.

**Conclusions:**

Food and waterborne diseases are a constant public health problem in many countries. Key challenges to the enhancement of food safety policy include the paucity of systematic data and awareness. With this background, ICMR’s initiative is the first systematic surveillance study in the country to adopt a single health approach. Data obtained from this project will help to understand the risk of acquiring food and waterborne pathogens, their transmission pathways, and antimicrobial resistance patterns. The scientific evidence generated through this project will be helpful in formulating and strengthening food safety policy and in initiating government programs to protect the health of the nation.

**International Registered Report Identifier (IRRID):**

DERR1-10.2196/56469

## Introduction

### Background

Food and waterborne diseases and outbreaks are emerging as a greater global health burden, analogous to malaria, HIV/AIDS, or tuberculosis. The globalization of the food trade, changing agriculture and animal farming practices, and rapid shifts in environmental factors have created conditions favorable for the emergence, re-emergence, and spread of food and waterborne pathogens, compounding the challenge of effectively responding to foodborne threats to public health [1].

More than 200 different infections are spread through contaminated food and water, ranging from diarrhea to cancer [2]. The World Health Organization (WHO) has estimated that 2 million deaths occur every year due to consumption of contaminated food or water and that almost 30% of foodborne infection–associated mortality cases occur among children younger than 5 years [1]. The reported incidence of foodborne diseases represents less than 1% to 10% of the actual number [3], as most patients with mild symptoms do not seek medical care, causing cases to go unreported [4].

The developing world is at a higher risk of foodborne diseases. South East Asia has the second highest burden of foodborne diseases after Africa. Moreover, the WHO has reported a high burden of diarrheal and invasive enteric diseases in the Indian region, which is nearly double the global average and 20 times higher than that in Europe [5]. The Ministry of Health and Family Welfare (MoHFW), Government of India has reported that food poisoning is one of the most common outbreaks reported apart from acute diarrheal disease. It was also noted that the incidence of acute diarrheal disease and contamination of food is high in places where food is cooked in bulk such as canteens, hotels, and wedding venues [6]. The coherent regulatory strategy adopted to reduce foodborne illness in India includes monitoring and surveillance programs for the assessment of biological risk and residues of various contaminants in food products through the Food Safety and Standards Authority of India (FSSAI) established under the Food Safety and Standards Act, 2006, and surveillance through the Integrated Disease Surveillance Project (IDSP) implemented by the National Centre for Disease Control (NCDC). India has a large unorganized food sector (roadside vendors, *dhabas*, food carts, street stalls, etc) that provides inexpensive food to the economically weaker sections. The hygiene and sanitary practices of street food vendors are a matter of grave concern [7]. Government schemes, such as *Clean Street Food Hubs*, *Clean and Fresh Fruit and Vegetable Markets*, and *Eat Right Campus* for schools, colleges, workplaces, and other campuses, for creating awareness around mindful eating have been successfully established in 2023 on a Pan-India basis. The Swachh Bharat Mission, which involves a sanitation initiative for open defecation-free status, has improved the overall situation regarding diarrheal diseases in India. However, there is a crucial need for systematic data to inform policy decisions and prevent the transmission of pathogens among humans, food, animals, and the environment.

A wide range of pathogenic microorganisms are capable of infecting food products before consumption [8,9]. The WHO has prioritized 22 enteric foodborne pathogens for assessing the burden of foodborne illness [7]. These infections are attributed to specific foods, such as chicken, precooked meat, fish, dairy products, fermented foods, fruits, and vegetables [1]. Food-producing animals (eg, cattle, chickens, pigs, turkeys, etc) are also major reservoirs for pathogens such as *Campylobacter* species, non-Typhi serotypes of *Salmonella enterica*, Shiga toxin–producing *Escherichia coli*, and *Listeria monocytogenes* [10]. Further, many pathogens that are commonly associated with food can also be transmitted indirectly through the environment, animals, or infected or carrier people [11]. However, without data on the major foodborne pathogens circulating in each region and their ability to cause foodborne diseases and outbreaks, it is difficult to make strong food safety guidelines.

The North-Eastern Region (NER) of India shares international borders with China, Bangladesh, Bhutan, Nepal, and Myanmar. The food habits and cultures of each state are different and influenced by the neighboring countries. The preferred diet of the North-Eastern population in India is mainly raw, boiled, fermented, smoked, grilled, and roasted food. The preparation of these traditional foods often lacks appropriate sanitary practices and involves uncontrolled natural fermentation, which can transmit pathogens from food to humans. Additionally, an irregular electricity supply may support the proliferation of pathogens due to temperature fluctuations that affect stored meats and other food items [12]. Although intermittent outbreaks due to the consumption of traditional and fermented foods in the NER are commonly reported, detailed scientific investigations or interventions are limited [13]. This is mainly attributed to insufficient laboratory support; limited collaboration with the NCDC, state health authorities, and community leaders; and inadequate support for researchers, outbreak investigations, training of field workers, and funding.

As the first step toward bridging this knowledge gap, the Indian Council of Medical Research (ICMR), New Delhi, undertook an initiative to conduct sentinel surveillance of foodborne pathogens through a strong laboratory network in the 8 states of North-East India. Here, we present the methodology that has been adapted to initiate the ICMR Foodborne Pathogen Surveillance and Research Network (ICMR-FoodNet), covering the entire NER of India in phases I and II. The successful deployment of this program in all states of the NER will serve as a strong foundation for extending the network to other states within the country. Phase II expansion holds the potential to strengthen the impact of ICMR-FoodNet on a national scale, thus enhancing the overall food safety landscape in India.

### Aims and Objectives

The overall aim is to identify food and waterborne pathogens to determine the risk factors that contribute to sporadic diarrheal outbreaks through population-based studies while examining the importance of different risks associated with food safety and hygiene practices as contributors to infections in the NER of India. The specific objectives are given in Textbox 1.

**Objectives for the foodborne pathogen surveillance.**

**Primary objectives**

- To identify the major circulating pathogens in humans, the food chain (food items and animal-sourced food), and the environment, causing food and waterborne diseases through market, hospital, animal husbandry, and community-based sentinel surveillance and outbreak investigations
- To document regional and seasonal variations in diarrheal diseases caused by food and waterborne pathogens
- To identify sources and pathogens causing outbreaks of food and waterborne diseases through systematic investigation of public health response
- To document genotyping and antimicrobial susceptibility patterns of identified bacterial pathogens

**Secondary objectives**

- To develop capacity for bacterial culture, antimicrobial susceptibility testing, molecular testing, metagenomics, and outbreak investigations at North-East institutes for food and waterborne pathogens
- To determine clonality using molecular tools to trace the spread of infections

## Methods

### Study Design

Laboratory-based multicentric sentinel surveillance is used to collect prospective data from markets (market surveillance); hospitals (hospital surveillance); and poultry farms, animal farms, slaughterhouses, and butcher shops (animal husbandry surveillance) and assess the outbreak situation through 12 ICMR-FoodNet centers.

### Study Setting

ICMR initiated a foodborne pathogen survey and research network (ICMR-FoodNet) in North-East India in 2020. In phase I, 5 sites were initiated in 4 states (Assam [Dibrugarh and Guwahati], Sikkim, Arunachal Pradesh, and Tripura). Based on the interim data of phase I, the ICMR-FoodNet is being expanded to the remaining 4 NER states (ie, Meghalaya, Manipur, Mizoram, and Nagaland) with 4 medical and 3 veterinary centers to cover the entire NER.

**Figure 1:**
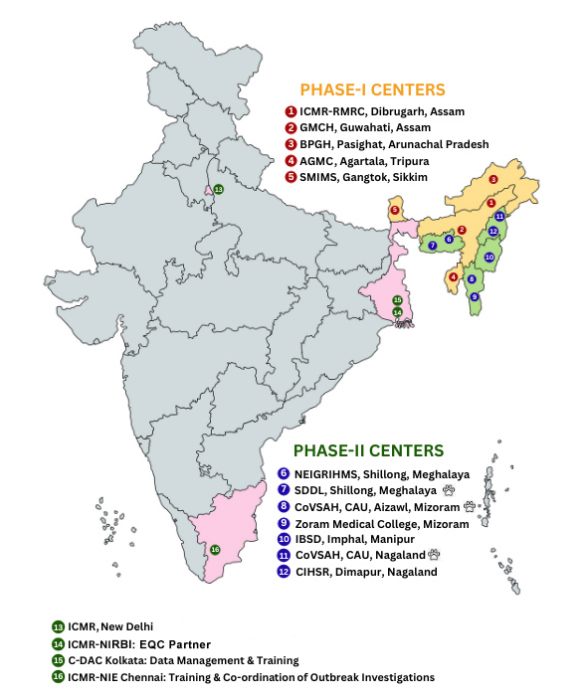
Geographical map of ICMR-FoodNet centers and partner sites. AGMC: Agartala Government Medical College; BPGH: Bakin Pertin General Hospital; CAU: Central Agricultural University; C-DAC: Centre for Development of Advanced Computing; CIHSR: Christian Institute of Health Sciences and Research; CoVSAH: College of Veterinary Science and Animal Husbandry; EQC: external quality control; FoodNet: Foodborne Pathogen Surveillance and Research Network; GMCH: Guwahati Medical College and Hospital; IBSD: Institute of Bioresources and Sustainable Development; ICMR: Indian Council of Medical Research; NEIGRIHMS: North Eastern Indira Gandhi Regional Institute of Health & Medical Science; NIE: National Institute of Epidemiology; NIRBI: National Institute for Research in Bacterial Infections; RMRC: Regional Medical Research Centre; SDDL: State Disease Diagnostic Laboratory; SMIMS: Sikkim Manipal Institute of Medical Sciences.

As shown in Figure 1, the ICMR-FoodNet centers include (1) ICMR-Regional Medical Research Centre (RMRC) Dibrugarh, Assam; (2) Guwahati Medical College and Hospital (GMCH), Guwahati, Assam; (3) Bakin Pertin General Hospital & Training Centre (BPGH&TC), Pasighat, Arunachal Pradesh; (4) Agartala Government Medical College (AGMC), Agartala, Tripura; (5) Sikkim Manipal Institute of Medical Sciences (SMIMS), Gangtok, Sikkim; (6) North Eastern Indira Gandhi Regional Institute of Health & Medical Science (NEIGRIHMS), Shillong, Meghalaya; (7) State Disease Diagnostic Laboratory, (SDDL), Shillong, Meghalaya; (8) College of Veterinary Science and Animal Husbandry (CoVSAH), Central Agricultural University (CAU), Aizawl, Mizoram; (9) Zoram Medical College (ZMC), Mizoram; (10) Institute of Bioresources and Sustainable Development (IBSD), Imphal, Manipur; (11) CoVSAH, CAU, Nagaland; and (12) Christian Institute of Health Sciences and Research (CIHSR), Dimapur, Nagaland. These centers have helped to develop a comprehensive laboratory network in the NER. ICMR-National Institute for Research in Bacterial Infections (NIRBI) and Centre for Development of Advanced Computing (C-DAC), Kolkata act as external quality assurance/training and data management centers, respectively.

The network provides advanced diagnostic services for all enteric pathogens, conducts laboratory training to develop skilled personnel, and investigates outbreaks and antimicrobial resistance (AMR).

### Duration

The duration of each phase is 5 years, including the identification of sites or institutions, laboratory and data entry training, and pilot studies.

### Case Definition

Case definitions adapted for this project are according to the guidelines of the IDSP, MoHFW, Government of India [14].

The term *foodborne disease*, including foodborne intoxication and foodborne infection, covers illnesses acquired through the consumption of contaminated food [15]. *Diarrhea* is defined as the passage of three or more loose watery stools in the past 24 hours (with or without vomiting) [16]. *Foodborne disease outbreaks* are defined as the occurrence of two or more cases of a similar illness resulting from ingestion of a common food or the observed number of cases of a particular disease exceeding the expected number. These can be confirmed (when at least one causal agent is identified) or suspected (based on clinical and epidemiological information). Although most cases are sporadic, these diseases draw attention to themselves due to outbreaks, and a thorough investigation can help in identifying control measures [15].

### Ethical Considerations

#### Approval

This protocol has received ethics approval from the ICMR-Central Ethics Committee on Human Research (ICMR-CECHR) (reference number: CECHR 003/2023). Additionally, approval has been obtained from the following institutional ethics committees: ICMR- RMRC, Dibrugarh, Assam (RMRC/Dib/IEC (Human)/2019-20/1883); GMCH, Guwahati, Assam (MC/190/2007/PI-11/Oct-2019/19); BPGH&TC, Pasighat, Arunachal Pradesh (IIP/IEC/07/2019); AGMC, Agartala, Tripura (4(6-11)-AGMC/Medical Education/Ethics Com./2018/27980); SMIMS, Gangtok, Sikkim (SMIMS/IEC/2019-116); NEIGRIHMS, Shillong, Meghalaya (NEIGR/IEC/M11/F33/2023); CoVSAH & CAU, Aizawl, Mizoram (CVSC/CAU/IAEC/22-23/P-2); ZMC, Mizoram (F.20016/1/18-ZMC/IEC/107); IBSD, Imphal, Manipur (IEC-02/2023-002); CoVSAH & CAU, Nagaland (1476/Go/Re/SL/11/CPCSEA); and CIHSR, Dimapur, Nagaland (027/2023-24/IEC-CIHSR). Memorandums of agreement have been signed between the ICMR and all FoodNet centers for shared principles, objectives, and responsibilities.

#### Consent

Written informed consent for hospital surveillance will be obtained from each patient among patients aged ≥18 years, from a parent or guardian among patients aged ≤10 years, and from a parent or guardian with additional assent among patients aged 11-17 years. Additionally, informed consent will be obtained from each food or animal handler for animal husbandry and market surveillance. Consent will also be obtained for the storage of clinically relevant enteric bacterial strains, stool sample collection, and the use of participant data for future studies.

#### Privacy and Confidentiality

All information and data obtained from the medical records of the patients for this project will be considered confidential. The digital documentation of the clinical data will take place in an anonymized fashion. Nonidentifiable data (eg, name and date of birth) will be entered into the database. There will be no pseudonyms, which would make a retrospective reidentification of the patient possible. All data and results will be stored for at least 10 years after the publication of the results.

#### Compensation Details

No compensation will be provided to participants in this surveillance.

### Standard Operating Procedure

The procedures of sample collection, transport, isolation, and identification of bacteria and viruses for the diagnosis of foodborne infections and their antimicrobial susceptibility testing have been detailed in the standard operating procedure presented in Multimedia Appendix 1.

### Setting of Data Collection

To cover a state comprehensively, 4 districts from each state will be chosen, covering East, West, North, and South zones, and within each district, 1 local market, 1 hospital, 1 poultry farm, 1 animal farm, 1 slaughterhouse, and 1 butcher area, covering both rural and urban areas, will be identified for sample collection. The shops in the market will be chosen randomly during each visit for food sample collection (twice a week) by the research teams. This sentinel surveillance is designed to be ongoing for at least 5 years. The sampling will be carried out throughout the year to monitor trends and detect outbreaks early.

Food handlers directly involved in preparing or handling suspected food items will be interviewed. Similarly, samples from diarrheal cases will be collected from health care facilities (primary health centers and community health centers or district hospitals having provision for the admission of patients) in each district and will be visited twice a week for clinical data and sample collection.

Outbreak investigations will be conducted based on “alerts” or “signals” from the community or IDSP. The NCDC-recommended 10-step approach for outbreak investigations will be followed to generate data [17]. The FoodNet team along with the state IDSP team will work together to investigate the outbreak.

### Sample Size

The sample size has been calculated based on the data collected and sample positivity in the first 3 months.

#### Market Surveillance

A total of 75 food samples, including cooked food items (n=30), uncooked food items (n=40), and traditional state-specific food items (n=5), will be collected from each state every month. Additionally, 20 samples from food handlers will be collected from each state every month.

#### Hospital Surveillance

Depending on the number of cases, about 20 diarrheal stool samples will be collected from each district every month.

#### Animal Husbandry Surveillance

A total of 20 samples from dead and slaughtered animals, and 60 animal food products will be collected from each state. Additionally, 20 samples from animal feed and fodder, and 20 samples from animal handlers and butchers will be collected from each state every month.

#### Outbreak Investigations

In large outbreaks, clinical samples will be collected from at least 15% to 20% of all cases manifesting illness (cases) and from an appropriate number of exposed but not ill people (controls). In smaller outbreaks, clinical samples will be collected from as many cases as practicable.

Five environmental samples, including water samples and surface swabs, will be collected monthly from hospitals, markets, butcher houses, and poultry farms. Additionally, during all outbreak investigations, communal water sources, along with the water and surfaces used in suspected settings, will be sampled.

### Sample Collection

For market surveillance, food items will be collected from restaurants, street food vendors, vegetable and fruit sellers, etc. They are broadly categorized as (1) cooked or processed food items (5 subcategories), (2) uncooked food items (6 subcategories), and (3) state-specific or traditional food items (Textbox 2). Swab samples from working surfaces used for cutting or chopping food items and refrigerator surfaces in shops will also be collected.

**Market surveillance: food item categorization.**

**Cooked food (restaurant/street food/mid-day meal)**

- Meat/fish/seafood (nonveg)
- Rice/flour/pulses
- Vegetables
- Milk products and sweets (processed)
- Refrigerated

**Uncooked food**

- Raw/dried meat
- Raw/dry fish
- Raw milk
- Whole or cut fruits and vegetables
- Frozen fruits and vegetables
- Dough

**State-specific food items (ethnic/traditional food items)**

- No soybean/legumes/cereal
- Fermented vegetables/bamboo/pickle
- Meat/fish/seafood (nonveg)
- Edible insects
- Leafy vegetables
- Beverages (milk/tea/alcohol)

For hospital surveillance, samples of stool, rectal swabs, and vomit will be collected from confirmed diarrheal disease patients who present to the hospitals. For animal husbandry surveillance, feces, rectal or cloacal swabs, and intestinal content from carcasses will be collected from poultry or animal farms, slaughterhouses, and butcher areas. Animal-sourced food samples (meat, milk, and eggs) will be collected. Moreover, animal feed and fodder samples will be collected. Furthermore, hand swab, nail bed swab, and nasal swab samples will be collected from suspected carriers (food and animal handlers).

Environmental samples, such as water from a stream or local pond, will be collected. Moreover, water used for cooking or drinking, food processing, hand washing, cleaning, and washing of utensils from market food shops and restaurants; farm water used for drinking and farm activities; and wash water of animal food products from poultry or animal farms and slaughterhouses will be collected.

Three types of samples will be collected for outbreak investigations: (1) clinical samples from patients (stool, rectal swabs, and vomit) and suspected carriers (nasal swabs, nail bed swabs, and rectal swabs); (2) suspected food samples (common foods consumed at events and gatherings, leading to an outbreak); and (3) environmental samples, such as water extracted from a communal source used by the community (eg, a nearby stream, pond, bore well, or tube well).

### Laboratory Investigations

A list of enteric pathogens to be screened from samples collected in market, hospital, and animal husbandry surveillance and outbreak investigations is presented in Table 1.

**Figure 2:**
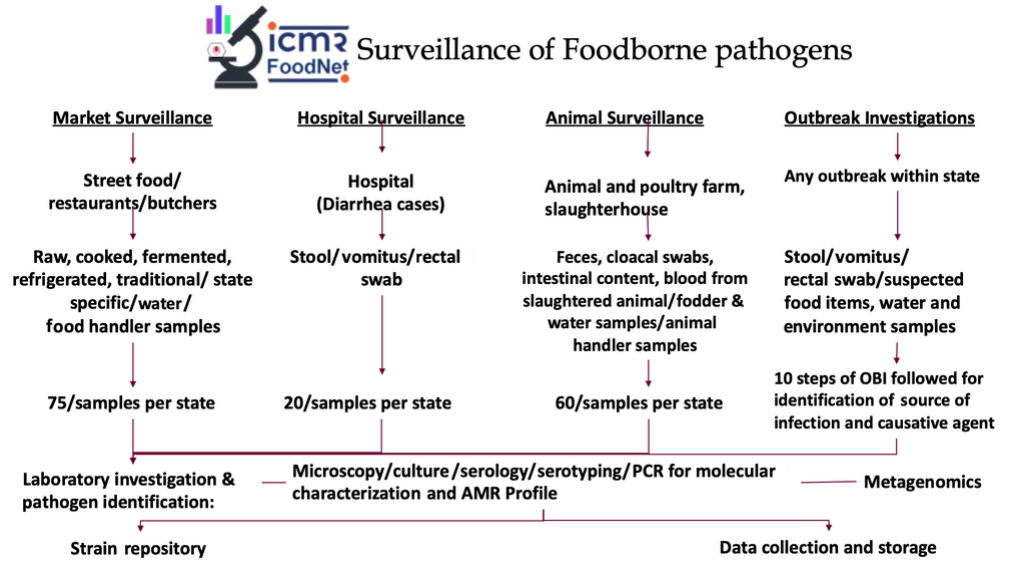
Flowchart of the work plan for sample collection and data analysis. AMR: antimicrobial resistance; OBI: outbreak investigation; PCR: polymerase chain reaction.

Culture and sensitivity testing will be performed along with identification by biochemical and serological tests. Polymerase chain reaction (PCR) assays will be performed for bacterial confirmation and detection of virulence genes. Stool samples will be tested by microscopy for the detection of parasites. Viral diagnostic kits (Vitassay qPCR, Spain) will be used during outbreak investigations for the detection of rotavirus, adenovirus, astrovirus, and norovirus. The work plan for the sample collection and data analysis is illustrated in Figure 2.

**Table 1:** List of pathogens to be screened from samples collected in market, hospital, and animal husbandry surveillance and outbreak investigations.

Sample type/source	Pathogens to be screened	Laboratory examination
Raw milk (locally produced and supplied), ice cream, and dry milk powder	*Listeria monocytogenes*, *Yersinia enterocolitica*, *Campylobacter* spp, diarrheagenic *Escherichia coli*^a^, *Staphylococcus aureus*, *Salmonella* spp, *Shigella* spp, *Brucella* spp, *Mycobacterium bovis*, hepatitis A virus (HAV), hepatitis E virus (HEV), and norovirus	Culture, biochemical tests, serotyping, PCR^b^ assays, and rapid diagnostic kits for HAV, HEV, and norovirus
Raw pork, beef, mutton, poultry meat, and bush meat	Diarrheagenic *Escherichia coli*^a^, *Staphylococcus aureus*, *Salmonella* spp, *Shigella* spp, *Yersinia enterocolitica*, *Clostridium perfringens*, *Listeria monocytogenes*, *Campylobacter* spp, *Clostridioides difficile*, *Brucella* spp, *Leptospira* spp, *Mycobacterium bovis*, parasite ova and cyst, HAV, HEV, and norovirus	*Culture, biochemical tests, serotyping, PCR assays, a* *nd* *rapid diagnostic kits for HAV, HEV, and norovirus*
Fish (raw fish or tinned fish)	Pathogenic *Vibrio parahaemolyticus*, *Vibrio cholerae* serogroups O1 and O139, diarrheagenic *Escherichia coli*^a^, *Salmonella* spp, *Staphylococcus aureus*, *Listeria monocytogenes*, *Clostridium botulinum*, *Clostridium perfringens*, *Campylobacter* spp, HAV, HEV, and norovirus	Culture, biochemical tests, serotyping, PCR assays, and rapid diagnostic kits for HAV, HEV, and norovirus
Raw vegetables	*Salmonella* spp, *Shigella* spp, diarrheagenic *Escherichia coli*^a^, *Vibrio cholerae* serogroups O1 and O139, *Salmonella* spp, *Listeria monocytogenes*, helminth ova and cyst, HAV, HEV, and norovirus	Microscopy, culture, biochemical tests, serotyping, PCR assays, helminth ova and cyst examination, and rapid diagnostic kits for HAV, HEV, and norovirus
Environment (water)	Diarrheagenic *Escherichia coli*^a^, *Staphylococcus aureus*, *Salmonella* spp, *Shigella* spp, *Yersinia enterocolitica*, *Clostridium perfringens*, *Listeria monocytogenes*, *Campylobacter* spp, *Vibrio cholerae* serogroups O1 and O139, *Staphylococcus aureus*, *Leptospira* spp, parasite ova and cyst, HAV, HEV, and norovirus	Microscopy, culture, biochemical tests, serotyping, PCR assays, parasite ova and cyst examination, and rapid diagnostic kits for HAV, HEV, and norovirus
Environment food (cooked rice as targeted sampling); Equipment (slicers, grinders, cutting boards, knives, and storage containers)	Diarrheagenic *Escherichia coli*^a^, *Staphylococcus aureus*, *Salmonella* spp, *Shigella* spp, *Yersinia enterocolitica*, *Clostridium perfringens*, *Listeria monocytogenes,* *Campylobacter* *spp,* *Vibrio cholerae* serogroups O1 and O139, pathogenic *Vibrio parahaemolyticus*, parasite ova and cyst, HAV, HEV, and norovirus	Microscopy, culture, biochemical tests, serotyping, PCR assays, and rapid diagnostic kits for HAV, HEV, and norovirus
Food items of suspected hepatitis patients	HAV, HEV, and norovirus	RT-PCR for HAV, HEV, and norovirus
Human (case) stool and vomitus; Human (control) stool	Diarrheagenic *Escherichia coli*^a^, *Salmonella* spp, *Shigella* spp, *Yersinia enterocolitica*, *Campylobacter* spp, *Vibrio cholerae* serogroups O1 and O139, pathogenic *Vibrio parahaemolyticus*, parasite ova and cyst, HAV, HEV, and norovirus	Microscopy, culture, biochemical tests, serotyping, PCR assays, parasite ova and cyst examination, and rapid diagnostic kits for HAV, HEV, and norovirus
Food handler (during outbreak) skin swabs, nasal swabs, etc	Diarrheagenic *Escherichia coli*^a^	Culture, biochemical tests, serotyping, and PCR assays
Animal tissues	*Leptospira* spp	Microscopy, culture, biochemical tests, serology, and PCR assays

^a^The pathotypes include enterotoxigenic *E. coli* (ETEC), enteropathogenic *E. coli* (EPEC), enteroaggregative *E. coli* (EAEC), enteroinvasive *E. coli* (EIEC), Shiga toxin–producing *E. coli* (STEC), and diffusely adherent *E. coli* (DAEC).

^b^PCR: polymerase chain reaction.

### Metagenomics Sample Analysis

Testing will be performed for (1) diarrheal stool samples in which at least one pathogen has been identified by culture or PCR, (2) healthy stool samples as controls from the same population, and (3) suspected food samples for outbreak or food poisoning and samples in which no pathogen was identified. DNA will be extracted from stool samples, and barcoded 16S rRNA gene amplification will be performed, followed by multiplexing of samples for amplicon sequencing.

### Quality Assurance

All identified bacterial strains will be preserved in Brain Heart Infusion (BHI)-Glycerol mix at –80 °C and transferred to ICMR-RMRC, Dibrugarh and ICMR-NIRBI, Kolkata for further confirmation, quality control, and genotyping.

### Strain Repository

A pathogen repository has been established at RMRC, Dibrugarh and NIRBI, Kolkata in phase I, to preserve fully characterized enteric pathogens for the maintenance and sharing of these pathogens for future research. The same arrangement will be continued in phase II.

### Data Management

A centralized, secured role-based web platform [18] has been designed and will be used by data collectors, lab technicians, and researchers for real-time data collection from hospitals, markets, animal farms, and outbreak locations. The data repository has been securely hosted on a cloud server. An electronic case report form (eCRF) has been designed with a drop-down menu to minimize data entry errors, and for additional security, the double data entry method will be followed. An ICMR-FoodNet mobile app has also been developed for data collection. The ICMR-FoodNet app will be maintained on a secure web server at C-DAC, Kolkata and ICMR, New Delhi. The study centers will register online to initiate the work. Site-based login credentials with access control will be used for data entry, data monitoring, and data download. Data validation will be performed by the principal investigator of the respective center. Data on patient age, sex, signs, symptoms, date of admission, date of discharge or death, diagnostic tests, identified pathogen, treatment history, outcome, etc will be recorded in the hospital eCRF record.

### Data Analysis

Descriptive analysis of the data will be performed by generating means and proportions. Bivariate and multivariate analyses will be undertaken. Further, analysis of the data will be performed based on a data analysis plan developed by the expert committee and requirements of the state health department.

### Dissemination of Data

All centers will conduct networking workshops to sensitize the stakeholders, IDSP officials, and state health officials about the project and induct them into the network. This includes timely dissemination of foodborne disease surveillance data for effective prevention. The involvement of local health agencies in reporting and disseminating information within communities will be emphasized. ICMR-FoodNet will collaborate with IDSP for outbreak investigations, and the resulting recommendations will be shared with state health officials for appropriate action. Communication strategies, such as newsletters, talks, and press releases, will be used to ensure the effective dissemination of information. Additionally, electronic dissemination methods through the ICMR-FoodNet website will be used for efficient data sharing in phase II.

### Project Management

ICMR, New Delhi functions as the coordinating site for planning, funding, and all logistic support. Around 10 eminent scientists (including chairpersons and co-chairpersons) with field expertise have been identified to formulate a project steering committee. These experts will review the task force proposals, suggest changes if any, and recommend for funding. In this project, ICMR-NIRBI, Kolkata is the external quality assurance service (EQAS) and laboratory training site, ICMR-National Institute of Epidemiology (NIE), Chennai is the outbreak investigation training site, and C-DAC, Kolkata is the data management site.

The project at each site will be overseen by an experienced microbiologist serving as the principal investigator, along with epidemiologists, social scientists (co-principal investigators), and a dedicated scientific research team. Regular visits by core committee members, monthly meetings with mentors, half-yearly reviews by the technical review committee, and centralized real-time data monitoring are the important measures implemented to ensure effective management and performance monitoring at each site. These strategies are essential for addressing issues promptly, ensuring data quality, and sustaining the surveillance system effectively.

## Results

This surveillance project has been systematically implemented in 2 phases. Five centers were established across 4 states in 2020 in phase I. Data collection began in October 2020 at all 5 study sites, after approval from the ethics committee. The ICMR-FoodNet surveillance system for monitoring foodborne diseases has successfully integrated with key organizations like the IDSP and FSSAI, which are the 2 prime organizations working on foodborne diseases in India under the MoHFW, Government of India. A secured FoodNet Digital platform has been developed for real-time data collection, role-based user dashboards, and a comprehensive e-Lab module [18]. Training sessions have been organized to instruct the project staff in laboratory techniques, data entry and recording, outbreak investigation and reporting, and metagenomic analysis. Following an interim data assessment and the successful establishment of a streamlined system for data procurement, investigation, recording, and analysis, along with the implementation of regular training and monitoring programs in phase I, phase II of the surveillance network was taken up.

**Figure 3:**
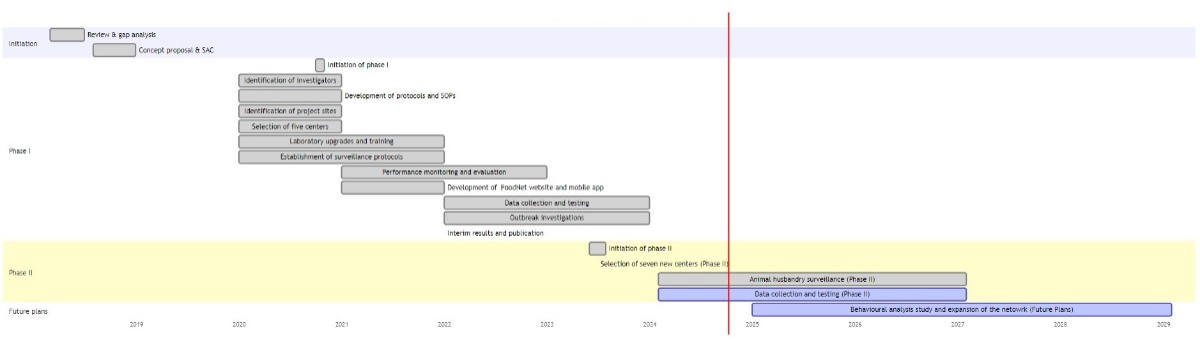
Strategic implementation and Gantt chart timeline overview of the ICMR-FoodNet surveillance project: A multiphase approach for enteric pathogen surveillance in North-East India. FoodNet: Foodborne Pathogen Surveillance and Research Network; ICMR: Indian Council of Medical Research; SOP: standard operating procedure.

The detailed process of developing the ICMR-FoodNet network through 8 systematic steps and the encountered challenges have been previously outlined [19] (Figure 3).

Phase II of the ICMR-FoodNet, initiated in January 2024, aims to expand the surveillance network to Manipur, Meghalaya, Mizoram, and Nagaland by establishing 7 new centers. From January to June 2024, the focus will be on upgrading laboratories for culture, antibiotic sensitivity testing, and molecular studies, alongside training staff in pathogen isolation, identification, and metagenomics. Starting in June 2024 and continuing through December 2027, systematic surveillance and data collection will involve food, clinical, animal, and environmental samples from designated districts. A key component is the animal surveillance module, monitoring poultry farms, animal farms, and slaughterhouses, with samples from animals, handlers, and the environment. Special attention will be given to enteric pathogen profiles in ethnic and traditional foods. Throughout this phase, foodborne and waterborne disease outbreaks will be systematically investigated, and the findings will be promptly reported to state health authorities for swift remedial actions. Continuous quality assurance and monitoring will be ensured through regular visits, meetings, and evaluations, supported by external quality assurance programs and centralized data monitoring. Ongoing data analysis using artificial intelligence and machine learning techniques will help identify trends and sources of foodborne illnesses, with a pathogen repository maintained for future research. By July 2027, further expansion to major metro cities and other states will be planned based on insights and funding, aiming for comprehensive national coverage. This phase will enhance existing infrastructure, integrate a comprehensive animal surveillance module, and focus on detailed data collection of enteric pathogens in ethnic foods, which will improve food safety and inform targeted public health interventions across North-East India.

## Discussion

### Impact of the ICMR-FoodNet Program

**Figure 4:**
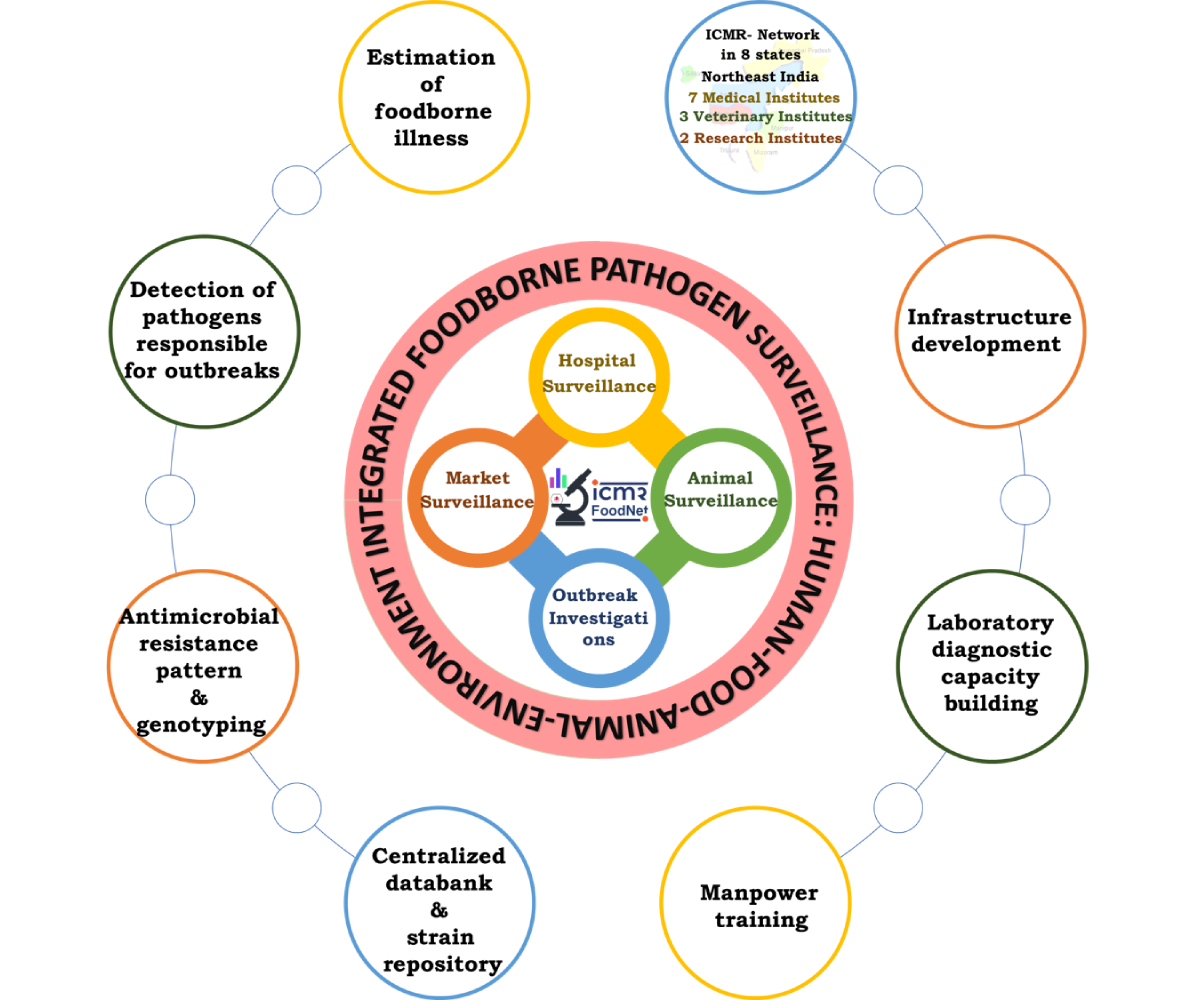
The modes of surveillance and outcomes of the ICMR-FoodNet program. FoodNet: Foodborne Pathogen Surveillance and Research Network; ICMR: Indian Council of Medical Research.

India encounters numerous challenges in safeguarding food safety, particularly in resource-limited states or areas of North-East India. Factors, such as culturally diverse populations, low economic development, inadequate sanitation, evolving dynamics in human-animal-environment interactions, and the emergence of AMR, collectively contribute to the prevalence of foodborne illnesses and outbreaks. Despite some prior fragmented studies in India, the real burden of foodborne illness remains significantly underreported and undetermined. The ICMR-FoodNet surveillance initiative is the first systematic study with a standardized methodology, targeting specific pathogens. It aims to generate dynamic region-specific data for monitoring common and emerging foodborne pathogens. This effort will contribute to updating Indian food safety and management protocols. Figure 4 illustrates the surveillance modes and outcomes of the ICMR-FoodNet program.

Laboratory-based surveillance provides crucial support in the timely reporting and monitoring of foodborne illnesses and outbreaks, essentially contributing to a well-informed public health response strategy [20,21]. Laboratory data play a vital role in understanding the source of the infection and implementing effective hygiene measures, especially for viral pathogens like norovirus, transmitted through both food and human contact, or rotavirus, which is known to cause high mortality among Indian children [22]. Recent surveillance of foodborne disease outbreaks in India reported 14.5% deaths in Assam, with only 6.4% outbreaks over 10 years, and concurrently, several states and union territories reported very few outbreaks in 10 years [23]. This emphasizes the importance of an improvement in surveillance and reporting, coverage of a large number of specimen tests during outbreaks and unified laboratory standards [24], generation of robust data and data analysis, constant monitoring by committees, and a higher priority for diseases of other vertical programs [25].

The initiative includes the enhancement of state-level laboratories to function as cutting-edge diagnostic facilities for foodborne pathogens. Through routine training programs, skilled manpower is generated, along with the establishment of standardized protocols for pathogen isolation and characterization. A robust laboratory network with internal and external quality assurance systems in place can be a valuable asset to the disease surveillance program. This project not only involves the collection of epidemiological data related to food hygiene and the prevalence of foodborne illnesses and outbreaks, but also strengthens the high-quality laboratory data on causative pathogens and their seasonal and regional trends. There is a significant shortage of manpower (particularly the ratio of food safety officers to the population) and a lack of sufficient resources, such as food testing laboratories, as noted by the IDSP. Integrating the ICMR-FoodNet with state health and national programs, especially during outbreak situations, will bolster the public health response in the region.

Despite the identified benefits of genomic surveillance of pathogens causing tuberculosis, malaria, HIV, SARS-CoV-2 infection, etc, the genomic information of enteric pathogens is comparatively low [21]. In order to enhance the methods of detecting and characterizing foodborne pathogens, profiling the virulence and pathogenicity attributes, monitoring AMR, tracking the source, and determining the causative pathogen during outbreak investigations, metagenomic studies will be undertaken in this project. All these aspects will focus on clinical and suspected food samples from outbreaks for characterizing known and unknown pathogens beyond the species or subspecies level (subtyping) in order to determine clonal relationships and trace the spread of infection in outbreak events. The development and validation of metagenome-based diagnosis could hold great potential for facilitating reliable and robust epidemiological investigations of enteric infections and disease surveillance, as culture-based techniques and PCR-based techniques have inherent technical limitations [26,27].

Many factors could be involved in food contamination, including those from the environment and human-related animal handling (slaughtering and processing practices, storage procedures, etc) [28]. Major foodborne pathogens, such as *Salmonella* and *Campylobacter*, originate from animals, are prevalent in farm environments, and are introduced into the food supply systems. Additionally, the indiscriminate use of antibiotics in livestock and agriculture has increased the rapid emergence of AMR pathogens and their transmission [29]. The ICMR-FoodNet integrated surveillance systems of human-food-animal-environment interactions will help understand multiple pathways of foodborne pathogen transmission and identify and address contamination risks in each stage of the food production chain [30]. Data analysis of the 3 main sectors (human, food, and animal) would allow a better understanding of risks across the food chain and source attribution, and facilitate a comprehensive estimation of the spread of AMR. This will help to formulate clinical management policy involving appropriate (eg, regarding AMR in humans) antimicrobial use policy in food-producing animals and horticulture [29,30] in alignment with the National Action Plan for containment of Antimicrobial Resistance (NAP-AMR) that was launched on April 19, 2017, by the NCDC and MoHFW, Government of India.

This project can bridge reporting gaps and initiate systematic collaborative outbreak investigations with IDSP and FSSAI officials. This effort will not only reduce the foodborne outbreak-associated losses in economic terms and human lives, but also enhance the robustness of the existing surveillance system and help national authorities to develop national plans and policies.

While this surveillance project aims to understand foodborne pathogen transmission and AMR in North-East India, several limitations must be acknowledged. The challenging geography and limited infrastructure of the NER of India, including difficult terrain, heavy monsoons, limited transportation, power shortages, and political unrest, can be a significant hurdle. Additionally, sociocultural diversity and language barriers may affect the uniformity of data collection. Owing to diverse farming practices and environmental conditions, the animal surveillance module may not capture all sources of enteric pathogens. The continuous nature of sentinel surveillance requires sustained funding and resources, which can fluctuate, impacting consistency. Recruiting and retaining skilled personnel, especially experienced bacteriologists, can further complicate efforts. Despite these challenges, the project’s structured approach aims to mitigate these issues and provide valuable insights into food safety and public health interventions.

### Future Plans

Future plans include a comprehensive situational analysis and behavioral assessment, particularly focusing on hygiene practices among food handlers, both at commercial and household levels. Furthermore, a study of mycotoxins in ethnic food samples has been planned. Consolidated data on mycotoxins will help to revise the food safety policy of the country. This network could be expanded to 4 major metro cities in India (ie, Delhi, Mumbai, Chennai, and Kolkata) to initiate nationwide coverage based on experiences from phases I and II and the availability of funds from ICMR/DHR, MoHFW. With accumulated data, proper risk analysis, including risk assessment, risk management, and risk communication, can be performed to estimate the burden of foodborne infections at the state and national levels.

### Conclusion

The ICMR-FoodNet program is of national importance as it covers 3 important aspects. The first aspect involves infrastructure development and capacity building of technical manpower in North-East India. The second aspect involves research to estimate the incidence of foodborne pathogens and AMR, and foodborne outbreak investigations for mitigating the risks associated with foodborne pathogens and reducing the incidence of foodborne infections. This project is aimed to provide data similar to the global estimated burden of foodborne illness. The third aspect involves the possible integration of the findings into national policy reforms in food safety in order to develop a well-aligned public health outbreak response.

## Appendix

Multimedia Appendix 1 Standard operating procedures for the Indian Council of Medical Research Foodborne Pathogen Surveillance and Research Network (North-East India).

## Abbreviations

AGMC: Agartala Government Medical College
AMR: antimicrobial resistance
BPGH&TC: Bakin Pertin General Hospital & Training Centre
CAU: Central Agricultural University
C-DAC: Centre for Development of Advanced Computing
CECHR: Central Ethics Committee on Human Research
CIHSR: Christian Institute of Health Sciences and Research
CoVSAH: College of Veterinary Science and Animal Husbandry
eCRF: electronic case report form
FoodNet: Foodborne Pathogen Surveillance and Research Network
FSSAI: Food Safety and Standards Authority of India
GMCH: Guwahati Medical College and Hospital
IBSD: Institute of Bioresources and Sustainable Development
ICMR: Indian Council of Medical Research
IDSP: Integrated Disease Surveillance Project
MoHFW: Ministry of Health and Family Welfare
NCDC: National Centre for Disease Control
NEIGRIHMS: North Eastern Indira Gandhi Regional Institute of Health & Medical Science
NER: North-Eastern Region
NIRBI: National Institute for Research in Bacterial Infections
PCR: polymerase chain reaction
RMRC: Regional Medical Research Centre
SMIMS: Sikkim Manipal Institute of Medical Sciences
WHO: World Health Organization
ZMC: Zoram Medical College

## Data Availability

All data underlying the results are available as part of the article, and no additional source data are required. The datasets used or analyzed during the study will be made available from the corresponding author upon reasonable request.
