# Supplementary material for: Surveillance of Food and Waterborne Pathogens in North-East India: Protocol for a Laboratory-Based Sentinel Surveillance Study
Source: JMIR Res Protoc. 2024 Oct 21;13:e56469. doi: 10.2196/56469 (PMC11535797; doi:10.2196/56469)

ICMR

INDIAN COUNCIL OF MEDICAL RESEARCH

Serving the nation since 1911

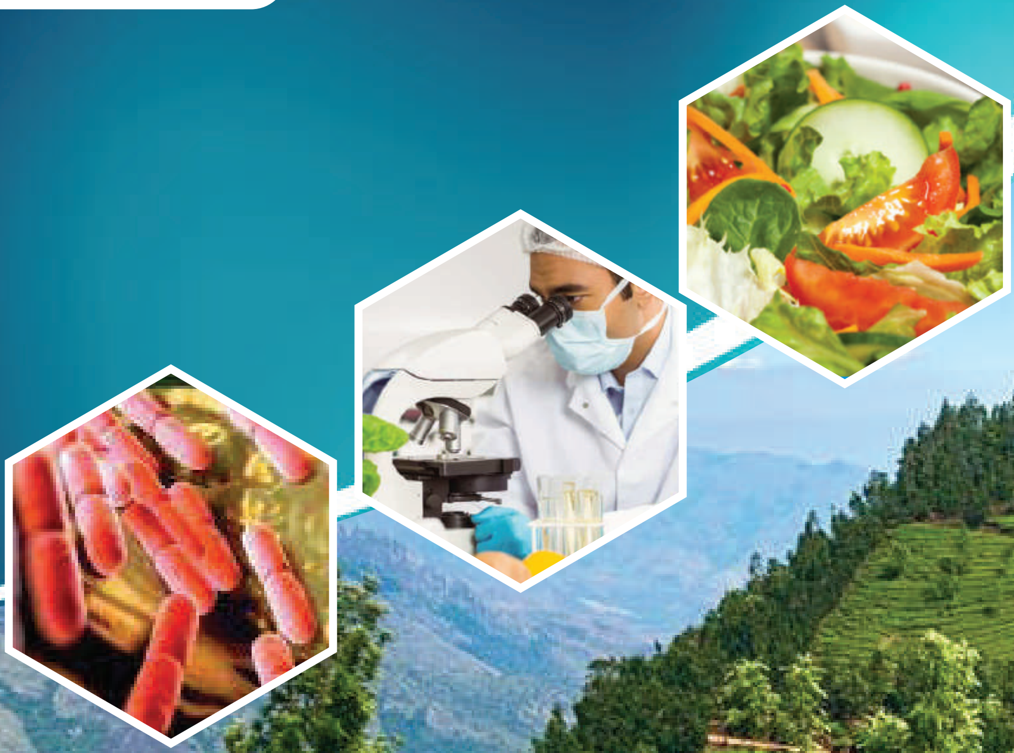

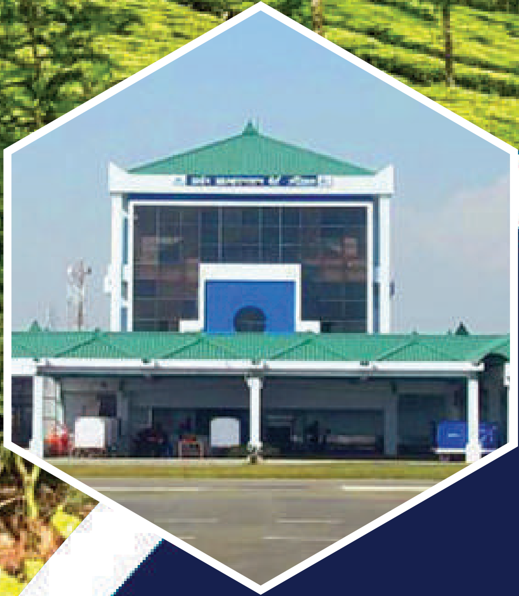

STANDARD OPERATING PROCEDURES

ICMR Foodborne pathogen survey and research Network (North-East India)

STANDARD OPERATING PROCEDURES

ICMR Foodborne Pathogen Survey and Research Network
(North-East India)

CONTRIBUTORS

STANDARD OPERATING PROCEDURES

ICMR Foodborne Pathogen Survey and Research Network
(North-East India)

Copyright © 2022 ICMR, New Delhi

All rights reserved. No part of this document may be reproduced or used in any other manner without prior permission of the copyright owner.

FOREWORD

Prof. (Dr.) Balram Bhargava
Secretary, DHR & Director General, ICMR

EDITORIAL BOARD

Prof. Iddya Karunasagar

Dr. T. Ramamurthy

Dr. Madhuchhanda Das

Dr. Utpala Devi

Dr. Pranjal Baruah

Dr. Samiran Panda

INSTITUTIONAL SUPPORT

Dr. Shanta Dutta,
Director, ICMR-NICED, Kolkata

Dr. Nabarun Bhattacharya,
Sr. Director, C-DAC, Kolkata

CREDITS

Technical Support:
ICMR Communication Unit

Dr. Rajni Kant
Director, ICMR-RMRC, Gorakhpur and
Scientist G & Head, Research Management, Policy, Planning and Coordination

Dr. Enna Dogra Gupta
Scientist C, Content Coordinator, Communications Unit
Research Management, Policy, Planning and Coordination

Dr. Priya Gaur
Scientist C, Content Coordinator, Communications Unit
Research Management, Policy, Planning and Coordination

Design and Production by:
Aakhya India (Media Consultant to ICMR)

प्रोफेसर (डा.) बलराम भार्गव, पदम श्री

एमडी, डीएम, एफआरसीपी (जी.), एफआरसीपी (ई.), एफएसीसी, एफएएचए, एफएएमएस, एफएनएएससी, एफएएससी, एफ एन ए., डी एस सी.

सचिव, भारत सरकार

स्वास्थ्य अनुसंधान विभाग

स्वास्थ्य एवं परिवार कल्याण मंत्रालय एवं

महानिदेशक, आई सी एम आर

Prof. (Dr.) Balram Bhargava, Padma Shri

MD, DM, FRCP (Glasg.), FRCP (Edin.), FACC, FAHA, FAMS, FNASc, FASc, FNA, DSc

Secretary to the Government of India

Department of Health Research

Ministry of Health & Family Welfare &

Director-General, ICMR

भारतीय आयुर्विज्ञान अनुसंधान परिषद

स्वास्थ्य अनुसंधान विभाग

स्वास्थ्य एवं परिवार कल्याण मंत्रालय

भारत सरकार

वी. रामलिंगस्वामी भवन, अंसारी नगर

नई दिल्ली - 110 029

Indian Council of Medical Research

Department of Health Research

Ministry of Health & Family Welfare

Government of India

V. Ramalingaswami Bhawan, Ansari Nagar

New Delhi - 110 029

FOREWORD

Foodborne diseases and outbreaks are the important public health hazards and causes of morbidity and mortality in developing countries. ICMR foodborne pathogens survey and research network from North East region is intended to address the food safety issue for Public Health management. This Standard Operating Procedure (SOP) details the procedures of sample collection, transport, isolation, identification of bacteria and viruses for diagnosis of foodborne infections and their antimicrobial susceptibility testing. This SOP is aimed to assist as a reference material for routine laboratory activities across the ICMR Foodnet centers from North-East India. This SOP was designed using standard methods to carry out different laboratory procedures as well as quality control and quality assurance in a concise manner. This is a base document not only for the use of ICMR Foodnet Research Network but it will help the researchers who are working on foodborne infections across the country.

The editorial board has done a commendable job in compiling and presenting the SOP in simple and precise manner. I applaud the diligent efforts of all the contributors. I hope that users will find it impactful and will be benefited immensely from this SOP. I am optimistic that this manual will meet its intended objectives and it will further evolve for clinical or research purposes through periodic revision and updates. I convey my best wishes to all.

Balram Bhargava

Prof. Balram Bhargava

Secretary, DHR & Director General, ICMR

Tele.: 26588204, 26589620, Fax (Off.) : 91-11-26588662, E-mail: secy-dg@icmr.gov.in

CONTENTS

1. Purpose 1
2. Scope 1
3. Associated documents 1
4. Safety 2
5. Methodology 3
   5.1 List of food items and the corresponding pathogens to be screened 3
   5.2 Sample collection and transportation 6
   5.3 Processing of different samples 9
   5.4 Procedure for isolation of pathogens 24
   - Salmonella & Shigella 24
   - Campylobacter 28
   - Escherichia coli O157 32
   - Multiplex PCR for the detection of E. Coli pathotypes (EAEC, EPEC, EHEC, ETEC, EIEC, DAEC) 33
   - Vibrio cholerae 37
   - Vibrio parahaemolyticus 42
   - Listeria monocytogenes 44
   - Bacillus cereus 46
   - Staphylococcus aureus 50
   - Yersinia enterocolitica 53
   - Hepatitis A virus (HAV) 56
   - Hepatitis E virus (HEV) 57
   - Norovirus 58
   - Parasites 59
6. Antibiotic susceptibility testing 60
7. Foodborne disease outbreak investigation 62
8. Quality control 63
9. Data recording and documentation 64
10. Report and sending of strains/DNA/RNA 64

Annexure-1 Tests for identification of Salmonella &Shigella 65
Annexure-2 Tests for identification of Campylobacter 69
Annexure-3 Tests for identification of Escherichia coli O157:H7 73
Annexure-4 Tests for identification of vibrios 74
Annexure-5 Tests for identification of Listeria monocytogens 75
Annexure-6 Tests for identification of Bacillus cereus 78
Annexure-7 Tests for identification of Staphylococcus aureus 83

CONTENTS

Annexure-8 Tests for identification of Yersinia enterocolitica **85**

Annexure-9 Long-term cold storage of bacterial strains **89**

Annexure-10 List of control strains **91**

Annexure-11 Over view of bacterial culture media and other tests for characterization **92**

Annexure-12 Equipment & Standards **95**

Annexure-13 Abbreviations **96**

Annexure-14 References **97**

Table S1. List of antimicrobials for susceptibility testing of foodborne pathogens 99

Table S2. Zone diameter and MIC breakpoints for Enterobacterales and Campylobacte 100

Table S3. Zone diameter and MIC breakpoints for non-Enterobacterales 101

Table S4. Disk diffusion QC ranges for Non-fastidious organisms and antimicrobial agents 102

1. PURPOSE

To be used as a standard operation procedure for isolation and identification of foodborne pathogens from different food sources, clinical specimens as well as environmental sources.

2. SCOPE

To find out the burden of foodborne pathogen disease burden
The methods described below outline the cultural procedures to isolate and identify foodborne pathogens as well as to characterize the isolates in respect of its antimicrobial susceptibility, serotype, genotype or toxin production.

3. ASSOCIATED DOCUMENTS

	Processing of milk for isolation of foodborne pathogens
	Processing of meat for isolation of foodborne pathogens
	Processing of fish for isolation of foodborne pathogens
	Processing of vegetables for isolation of foodborne pathogens
	Processing of dried foods like rice for isolation of foodborne
	Processing of stool for isolation of foodborne pathogens
	Processing of environmental swabs for isolation of foodborne pathogens
	Processing of skin and nasal swabs from food handlers for isolation of
	Isolation & identification of
	Isolation & identification of
	solation and identification of enterohaemorrhagic
	Isolation and Identification *Vibrio Cholerae/ Vibrio parahaemolyticus*
	Isolation and Identification *ae /Vibrio parahaemolyticus*
	solation and identification of *Listeria monocytogenes*
	solation and identification of
	solation and identification of
	Steps for isolation and identification of
	ests for identification of
	ests for identification of
	ests for identification of
	ests for identification of *Listeria monocytogenes*
	ests for identification of
	ests for identification of
	ests for identification of
	torage of bacterial strains
	view of bacterial culture media and other tests for characterization
	Equipment & Standards

4. SAFETY

- Carry out all procedures in accordance with the local codes of safe practice
- Universal precautions such as barrier protection, hand washing, safe techniques, safe handling of sharp items & specimen are used.
- All potentially contaminated materials (culture tubes, plates, glass slides, gloves, tips etc.) are disposed after decontamination. Any spills should be wiped up thoroughly using Lysol solution.
- Wear gloves and don't touch your eyes, nose, or other exposed membranes or skin with gloved hand.
- Wash hands thoroughly with soap and running water immediately after work. If gloves are worn, wash your hands with soap and water after removing the gloves.
- Wear laboratory coat, when working in the laboratory. Remove this protective clothing before leaving the laboratory. While handling stool specimens, use personal protective equipment (PPE).
- Never pipette by mouth.
- Care should be taken when using sharp or pointed instruments.
- Broken glass should be picked up with a brush and pan. Hands must never be used. Chipped or cracked glassware should not be used.
- In case of a wound or cut in hand, do not perform the test.
- Do not eat, drink, smoke, and apply cosmetics or store food or personal items in the laboratory where specimens or kit reagents are being handled.
- Paperwork should not be done on a potentially contaminated surface/working table in the testing laboratory.
- Disinfect work surfaces when procedures are completed at the end of each working day.

5. METHODOLOGY

5.1 LIST OF FOOD ITEMS AND THE CORRESPONDING PATHOGENS TO BE SCREENED

	sources/environment	Pathogens to be screened
	produced and supplied)	*Diarrhoeagenic Escherichia coli* (DEC) [ETEC, EPEC, EAEC, EIEC, STEC and DAEC]<br>Hepatitis A Virus (HAV)
		Diarrhoeagenic Escherichia coli (DEC) [ETEC, EPEC, EAEC, EIEC, STEC and DAEC]<br>*Clostridium perfringens*<br>*Listeria monocytogenes*<br>HAV, HEV and Norovirus
		Vibrio parahaemolyticus<br>serogroups O1 and O139<br>*Diarrhoeagenic Escherichia coli* (DEC) [ETEC, EPEC, EAEC, EIEC, STEC and DAEC]<br>*Staphylococcus aureus*<br>*Listeria monocytogenes*<br>*Clostridium perfringens*

	sources/environment	Pathogens to be screened
		Diarrhoeagenic Escherichia coli (DEC) [ETEC, EPEC, EAEC, EIEC, STEC a<br>serogroups O1 and O139<br><br>*Listeria monocytogenes*
		Diarrhoeagenic Escherichia coli (DEC) [ETEC, EPEC, EAEC, EIEC, STEC and DAEC]<br><br>*Clostridium perfringens*<br>*Listeria monocytogenes*<br><br>*Vibrio cholerae serogroups O1 and O139*

DURING OUTBREAKS

			Pathogens to be screened
			Diarrhoeagenic Escherichia coli (DEC) [ETEC, EPEC, EAEC, EIEC,<br><br>*Vibrio cholerae serogroups O1 and O139*<br>*Pathogenic Vibrio parahaemolyticus*
			Diarrhoeagenic Escherichia c (DEC) [ETEC, EPEC, EAEC, EIEC,
			Diarrhoeagenic Escherichia coli<br><br>*Clostridium perfringens*<br>*Listeria monocytogenes*<br><br>*Vibrio cholerae serogroups O1 and O139*<br>*Pathogenic Vibrio parahaemolyticus*<br><br>HAV, HEV and Norovirus

5.2 SAMPLE COLLECTION AND TRANSPORTATION

Procedures and equipment for specimen collection

FOOD SAMPLES (Following the sampling protocol mentioned in Bacteriological Analytical Manual, FDA)

General
The samples will be collected aseptically. It will be put into sterile jars or plastic bags to avoid any cross-contamination

Obtain samples of approximately 20-50 gm or 20 ml.

Sampling Unit (SU): 100 gm; A sample unit may consist of more than one container when containers are smaller than 100gm (e.g., four 25g containers could constitute a sample unit, these four 25gm containers are considered as sub-samples of a sample).

The packaged foods will be taken to the laboratory in their original containers/wraps.

The original packages or containers will be checked and recorded for code numbers that can be used to identify the place and time of processing. Include any unopened packages or cans belonging to the same batch.

The samples of perishable foods shall be refrigerated at 4°C until they can be examined. [Do not freeze food samples as certain pathogens (e.g., Gram-negative bacteria, vegetative forms of Clostridium perfringens) die off rapidly when frozen, but foods that were frozen when collected should be kept frozen until examined].

Meat or fish
A portion of food (usually SU: 100gm of meat or SU: 250 gm of fish) shall be cut or separated out using a sterile knife or other utensil if necessary. It will be collected aseptically and put into a sterile plastic bag or wide-mouth jar. Collection will be done from top centre and elsewhere of the samples, as necessary, and refrigerated.

Liquid food or beverages

At first samples are to be stirred or shaken. Collection of samples will be done using one of the following methods: Using a sterile utensil, transfer approximately SU: 20 ml into a sterile container; refrigerate.

Frozen foods
They should be kept frozen, using dry ice as necessary. Transportation shall be done in an insulated container.

Dried foods
Samples shall be collected using a sterile spoon, spatula, or similar utensil and transferred to a sterile water- and airtight container.

Environmental swabs from food, equipment, pipes, slicers, grinders, cutting boards, knives, storage containers filter etc.

Moisten swabs with 0.1% peptone water or buffered distilled water and wipe over contact surfaces of equipment or environmental surfaces. Place in enrichment broth.

WATER
Water samples can be collected in narrow mouthed glass sterile bottles. The appropriate volume of the sample should be about SU: 2-4 liters. Transport of samples should be made in ice chests and processed within 2 hrs after collection. Use sterile membranes for filtering the water samples using a vacuum pump and filtration assembly (Millipore). Avoid leakage of water while processing.

1) Water samples should be collected in the narrow mouthed heat sterilized glass bottles containing freshly prepared sodium thiosulphate (1.8% w/v) to neutralise any chlorine contamination (1ml per 1lit) of water collected.
2) The water is allowed to drain for 2-3 minutes before sample collection.
3) For collection from stream/lakes/ponds etc., the collection of the sample should be done at a depth of 30 cm, with the mouth of the bottle facing the current direction of running water.
4) The stopper/lid to be put into the mouth of the bottle and properly labelled with full details of place, source, time and date of collection.

5) The collected samples need to be transported to the laboratory within 6 hrs under cold condition (4°C) using ice/gel pack.

STOOL SAMPLES

Two containers of about 25 ml capacity, screw capped, wide mouthed plastic bottle, preferably with spoon shall be used to collect 1-2 ml of faeces. The specimen shall be transported immediately to the laboratory. If delay is unavoidable in one of the containers the faeces shall be collected in a container holding about 6 ml buffered glycerol transport medium for isolation of bacteria. To permit diagnosis of certain viral agents the other container shall be immediately refrigerated at 4°C (do not freeze) and sent as soon as possible to the laboratory.

If faecal samples cannot be obtained rectal swabs shall be collected

Procedure for collection of rectal swabs

1. Insert swab into Cary-Blair medium to moisten it
2. Insert swabs 3-5 cm into rectum and rotate gently
3. Remove swab and examine it to ensure that the cotton tip is stained with faeces.
4. Insert swabs immediately into tube of transport medium (Cary-Blair)
5. Push the swabs to the bottom of the tube
6. Break off the top parts of the sticks and tighten screw cap firmly.

For isolation of V. cholerae use Cary-Blair transport medium kept at room temperature (24-30°C).

SKIN LESIONS (boils, lesions, abscesses, secretions); Only during Outbreak Investigation

Clean the skin with normal saline or weak disinfectant. Apply pressure to the lesion using sterile gauzes and collect specimens on sterile swab, trying to obtain as much secretion as possible.

Transport immediately to laboratory at ambient temperature. If this is not possible, the specimen can be left for up to 24 hrs, at which time the swab should be placed in a container of ice.

NASAL SWABS; Only during Outbreak Investigation

Collect specimen with a sterile swab and immediately place in transport medium (e.g., Stuart's).

Transport immediately to laboratory at ambient temperature. If this is not possible, the specimen can be left for up to 24 hrs, at which time the swab should be placed in a container of ice.

5.3 PROCESSING OF DIFFERENT SAMPLES

Processing of Raw Milk, Pasteurised Milk, Dry Milk Powder and Milk Products

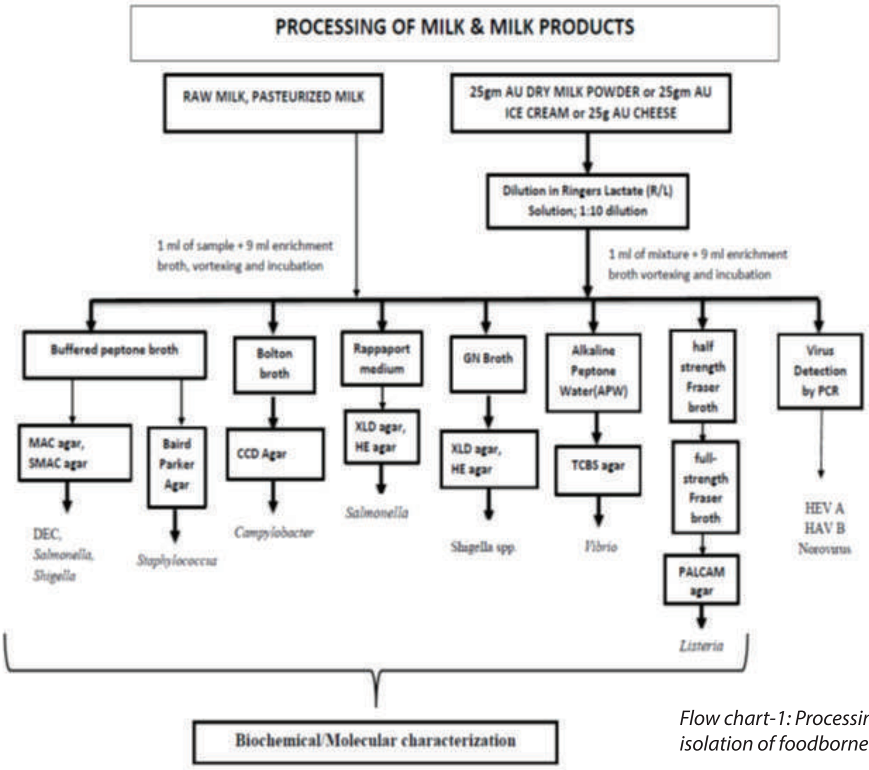

Flow chart-1: Processing of milk for isolation of foodborne pathogens

Procedure

1. From the SU, weigh aseptically 25gm AU Dry milk powder, cheese shall be diluted in 225 ml Ringers lactate in 1 in 10 dilutions
2. One ml of the diluted dry milk powder or cheese or 1 ml of raw milk, pasteurized milk shall be inoculated into 9 ml each of the following broths for enrichment: buffered peptone broth for enrichment of DEC, Salmonella, Shigella; Selenite F broth for enrichment of Shigella; Rappaport-Vassiliadis (RV) medium for enrichment of Salmonella; APW for enrichment of vibrios and half strength Fraser broth for enrichment of Listeria. Mix gently by vortexing.
3. After overnight incubation at 37°C, the sample shall be inoculated from Buffered peptone broth to a plate of MAC, SMAC, Baird Parker and CCD agar for isolation of DEC, Salmonella, Shigella, Staphylococcusaureus and Campylobacter, respectively.
4. Following 18-24 hrs of incubation, the growth from Rappaport-Vassiliadis (RV) medium and Selenite F broth is plated to HE (or XLD). The plates are incubated for 18-24 hrs at 37°C in a non-CO2 incubator for isolation of Salmonella and Shigella.
5. Following an incubation of 4~6 hrs at 37 C for enrichment, the growth from APW is subcultured on TCBS for isolation of vibrios.
6. For isolation of Listeria, with 0.1 ml of pre-enriched Fraser broth content in 10 ml of Fraser broth containing selective supplements for 48 hrs at a 37°C incubation temperature will be done.The inoculum will be plated on PALCAM agar and incubated for 48 hrs at 37°C for isolation.
7. Virus will be detected by RT-PCR directly after extraction of RNA from the diluted sample.

PROCESSING OF RAW MEAT

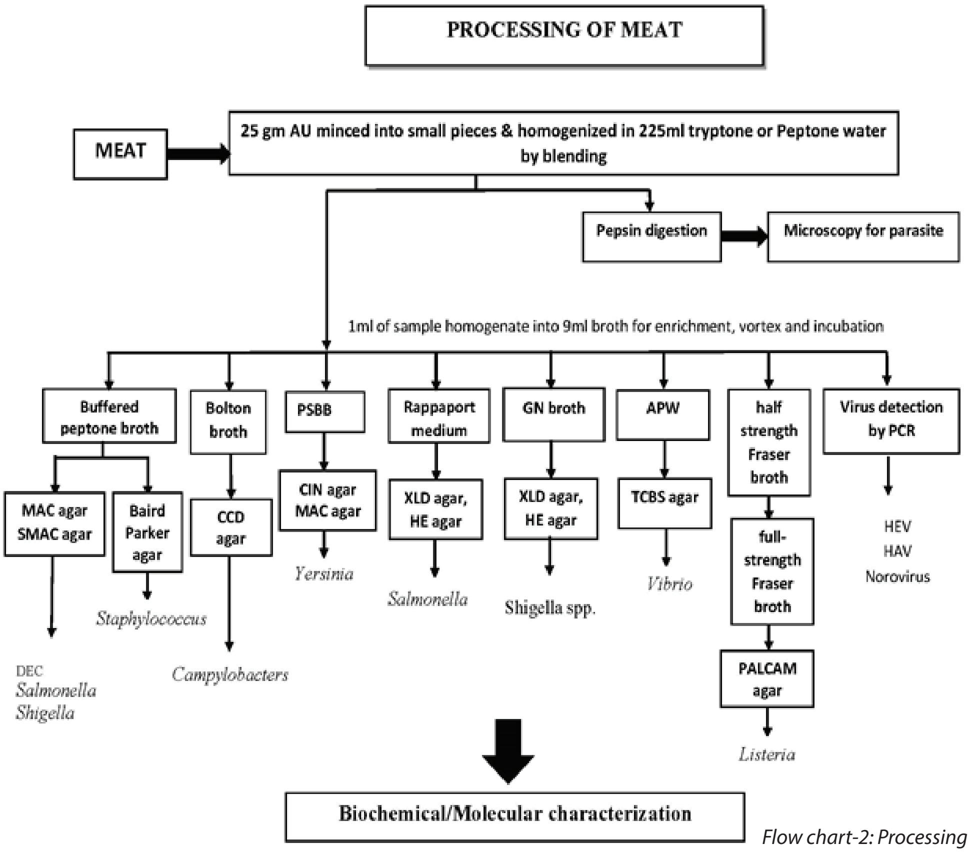

Flow chart-2: Processing of meat for isolation of foodborne pathogens

Procedure

1. Aseptically weigh 25gm AU of fish and other edible animals (frog, crab, snail, etc.,) from SU and out into a sterile wide mouth container will be minced into small pieces by a sterile scissor and homogenized in 225 ml tryptone or peptone water or PSBB by blending.
2. One ml of the sample homogenate shall be inoculated into each of the following broth (9 ml) for enrichment: buffered peptone broth for enrichment of DEC, Salmonella, Shigella; Selenite F broth for enrichment of Shigella; Rappaport-Vassiliadis (RV) medium for enrichment of Salmonella; PSBB for enrichment of Yersinia; APW for enrichment of vibrios and half strength Fraser broth for enrichment of Listeria. Mix gently by vortexing.
3. After overnight incubation at 37°C, 0.1 ml of the sample shall be inoculated from Buffered peptone broth to a plate of MAC, SMAC, Baird Parker and CCD agar for isolation of DEC, Salmonella, Shigella, Staphylococcus aureus and Campylobacter, respectively.
4. Following 18-24 hrs of incubation, the growth from Selenite F broth and Rappaport-Vassiliadis (RV) medium is plated to HE (or XLD). The plates are incubated for 18-24 hrs at 37°C in a non-CO2 incubator for the isolation of Shigella and Salmonella.
5. Following an incubation of 4~6hrs at 37°C for enrichment the growth from APW is subcultured on TCBS. The plates are incubated overnight for 18-24 hrs at 35°±2°C in a non - CO2 incubator for isolation of vibrios.
6. The PSBB is incubated at 10°C for 10 days, after which it is streaked on CIN and MacConkey plates for isolation of Yersinia.
7. For isolation of Listeria, with 0.1 ml of pre-enriched Fraser broth content in 10 ml full strength Fraser broth containing selective supplements for 48 hrs at a 37°C incubation temperature will be done. The inoculum will be plated on PALCAM agar and incubated for 48 hrs at 37°C for isolation.

9. Virus will be detected by RT-PCR directly after extraction of RNA from the diluted sample.
10. For parasite microscopic detection homogenization is done with saline and digestion in a mixture of pepsin in saline. After centrifugation the deposit is observed for parasite ova or cyst.

PROCESSING OF FISH AND OTHER EDIBLE ANIMALS

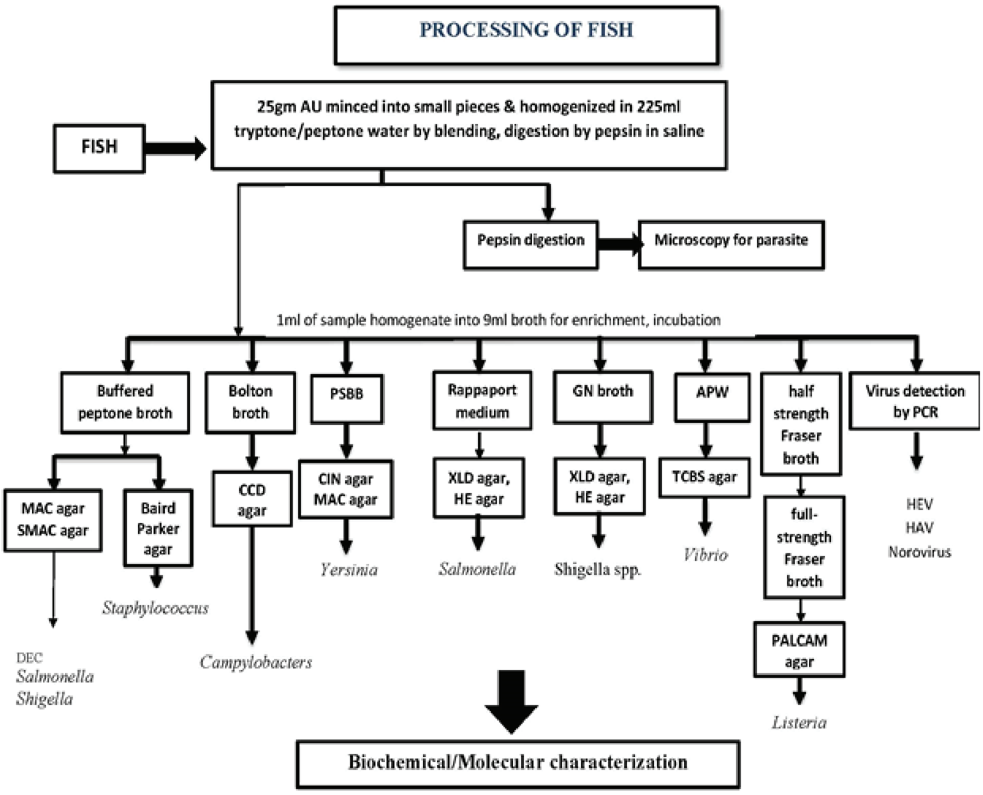

Flow chart-3: Processing of fish for isolation of foodborne pathogens

Procedure

1. Aseptically weigh 25gm AU of fish and other edible animals (frog, crab, snail, etc.,) from SU and out into a sterile wide mouth container will be minced into small pieces by a sterile scissor and homogenized in 225ml tryptone or peptone water or PSBB by blending.
2. One ml of the sample homogenate shall be inoculated into each of the following broth (9 ml) for enrichment: buffered peptone broth for enrichment ofDEC, Salmonella, Shigella; Selenite F broth for enrichment of Shigella; Rappaport-Vassiliadis (RV) medium for enrichment of Salmonella; PSBB for enrichment of Yersinia; APW for enrichment of vibrios and half strength Fraser broth for enrichment of Listeria. Mix gently by vortexing.
3. After overnight incubation at 37°C, 0.1 ml of the sample shall be inoculated from Buffered peptone broth to a plate of MAC, SMAC, Baird Parker and CCD agar for isolation ofDEC, Salmonella, Shigella, Staphylococcus aureus and Campylobacter, respectively.
4. Following 18-24 hrs of incubation, the growth from Selenite F broth and Rappaport-Vassiliadis (RV) medium is plated to HE (or XLD). The plates are incubated for 18-24 hrs at 37°C in a non-CO2 incubator for the isolation of Shigella and Salmonella.
5. Following an incubation of 4~6hrs at 37°C for enrichment the growth from APW is subcultured on TCBS. The plates are incubated overnight for 18-24 hrs at 35°±2°C in a non-CO2 incubator for isolation of vibrios.
6. The PSBB is incubated at 10°C for 10 days, after which it is streaked on CIN and MacConkey plates for isolation of Yersinia.
7. For isolation of Listeria, with 0.1 ml of pre-enriched Fraser broth content in 10 ml full strength Fraser broth containing selective supplements for 48 hrs at a 37°C incubation temperature will be done. The inoculum will be plated on PALCAM agar and incubated for 48 hrs at 37°C for isolation.
9. Virus will be detected by RT-PCR directly after extraction of RNA from the diluted sample.
10. For parasite microscopic detection homogenization is done with saline and digestion in a mixture of pepsin in saline. After centrifugation the deposit is observed for parasite ova or cyst.

PROCESSING OF VEGETABLES

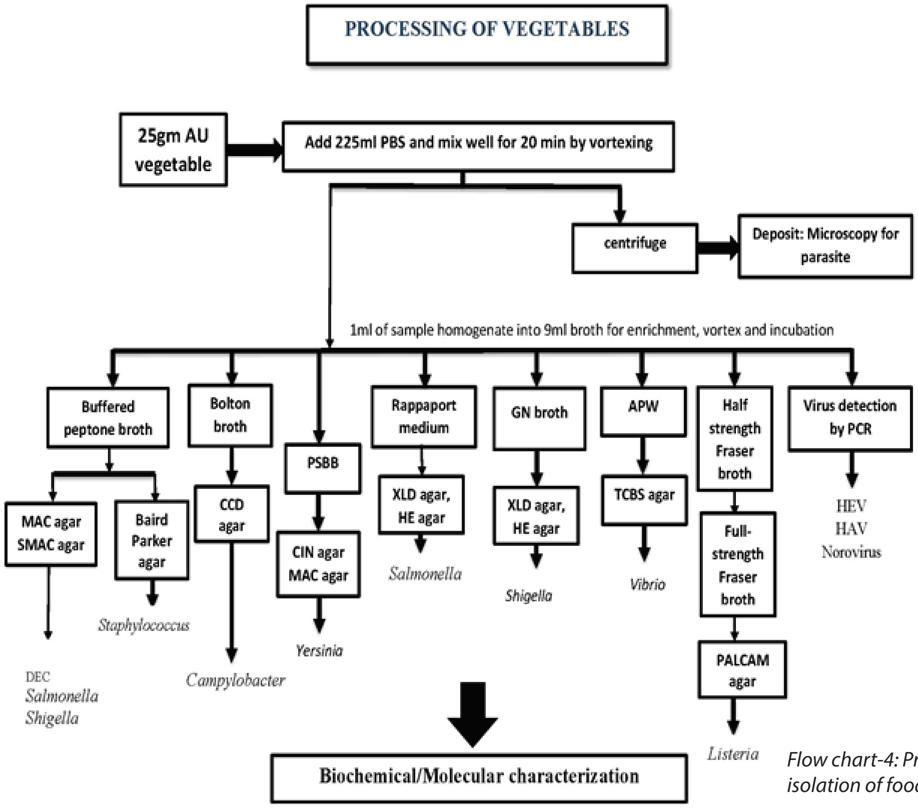

Flow chart-4: Processing of vegetables for isolation of foodborne pathogens

Procedure

1. From 100gm SU of vegetables, aseptically weigh 25g AU will be mixed with 225ml PBS and mixed well by swirling using sterile glass rod/spoon for 20 minutes. Incubate at 35° ± 2.0° C for 24 ± 2.0 hours.
2. One ml of the sample homogenate shall be inoculated into each of the following broth (9 ml) for enrichment: buffered peptone broth for enrichment of DEC, Salmonella, Shigella; Selenite F broth for enrichment of Shigella; Rappaport-Vassiliadis (RV) medium for enrichment of Salmonella; PSBB for enrichment of Yersinia; APW for enrichment of vibrios and half strength Fraser broth for enrichment of Listeria. Mix gently by vortexing.
3. After overnight incubation at 37°C, 0.1 ml of the sample shall be inoculated from Buffered peptone broth to a plate of MAC, SMAC, Baird Parker agar for isolation of DEC, Salmonella, Shigella, Staphylococcus aureus, respectively.
4. Following 18-24 hrs of incubation, the growth from Selenite F broth and Rappaport-Vassiliadis (RV) medium is plated to HE (or XLD). The plates are incubated for 18-24 hrs at 37°C in a non-CO2 incubator for the isolation of Shigella and Salmonella.
5. Following an incubation of 4~6 hrs at 37°C for enrichment the growth from APW is subcultured on TCBS. The plates are incubated overnight for 18-24 hrs at 35°±2°C in a non-CO2 incubator for isolation of vibrios.
6. The PSBB is incubated at 10°C for 10 days, after which it is streaked on CIN and MacConkey plates for isolation of Yersinia.
7. For isolation of Listeria, with 0.1 ml of pre-enriched Fraser broth content in 10 ml full strength Fraser broth containing selective supplements for 48 hrs at a 37°C incubation temperature will be done. The inoculum will be plated on PALCAM agar and incubated for 48 hrs at 37°C for isolation.
8. Virus will be detected by RT-PCR directly after extraction of RNA from the diluted sample.
9. For parasite microscopic detection each vegetable sample was eluted by vigorous agitation in a water bath in 1 L of sterile phosphate-buffered saline (pH 7.4), to which 50 ml of 0.01% Tween 80 will be added. The eluent was filtered through gauze and then dispensed into clean centrifuge tubes and centrifuged at 2000×g for 30 min. The centrifuged deposit will be observed for parasite ova or cyst.

PROCESSING OF FOODS: RICE

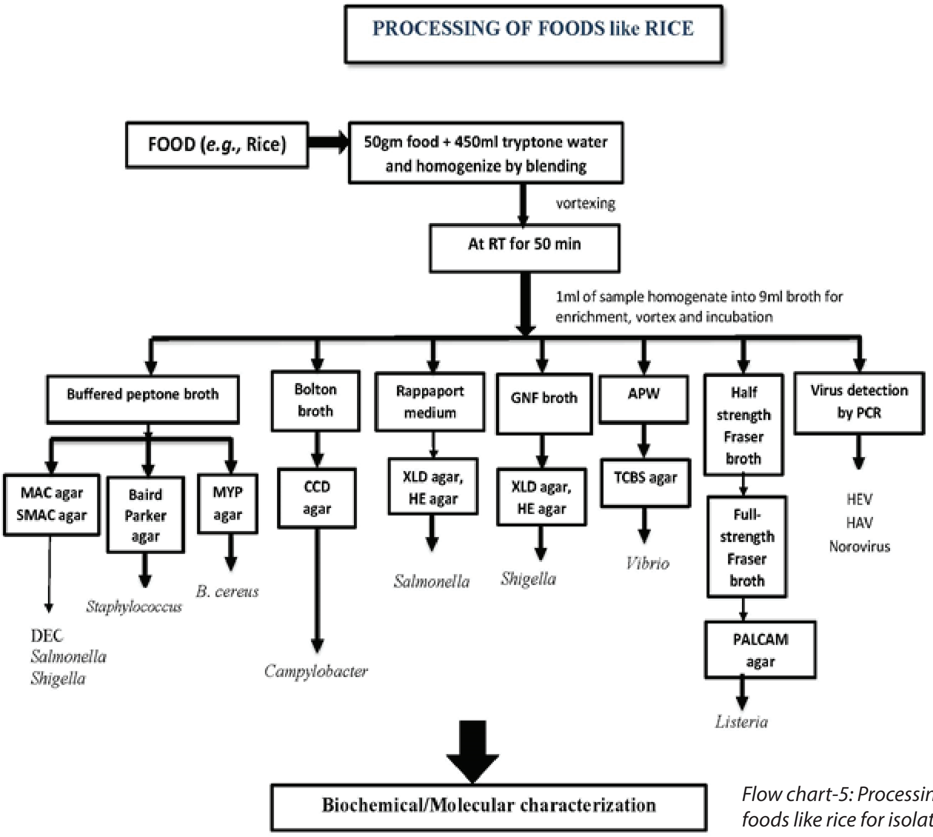

Flow chart-5: Processing of dried foods like rice for isolation of foodborne pathogens

Procedure

1. Fifty gm AU of food like rice will be mixed with 450 ml of tryptone water, homogenize and mixed well by blending for 50 minutes at room temperature.
2. One ml of the mixture homogenate shall be inoculated into each of the following broth (9 ml) for enrichment: buffered peptone broth for enrichment ofDEC, Salmonella, Shigella, B. cereus; Selenite F broth for enrichment of Shigella; Rappaport-Vassiliadis (RV) medium for enrichment of Salmonella; APW for enrichment of vibrios and half strength Fraser broth for enrichment of Listeria. Mix gently by vortexing.
3. After overnight incubation at 37°C, 0.1 ml of the sample shall be inoculated from Buffered peptone broth to a plate of MAC, SMAC, Baird Parker, CCD, MYP agar for isolation ofDEC, Salmonella, Shigella, Staphylococcus aureus, Campylobacter and Bacillus cereus, respectively.
4. Following 18-24 hrs of incubation, the growth from Selenite F broth and Rappaport-Vassiliadis (RV) medium is plated to HE (or XLD). The plates are incubated for 18-24 hrs at 37°C in a non-CO2 incubator for the isolation of Shigella and Salmonella.
5. Following an incubation of 4~6hrs at 37°C for enrichment the growth from APW is subcultured on TCBS. The plates are incubated overnight for 18-24 hrs at 35°±2°C in a non-CO2 incubator for isolation of vibrios.
6. For isolation of Listeria, with 0.1 ml of pre-enriched Fraser broth content in 10 ml full strength Fraser broth containing selective supplements for 48 hrs at a 37°C incubation temperature will be done. The inoculum will be plated on PALCAM agar and incubated for 48 hrs at 37°C for isolation.
7. Virus will be detected by RT-PCR directly after extraction of RNA from the diluted sample.

PROCESSING OF STOOL SAMPLES

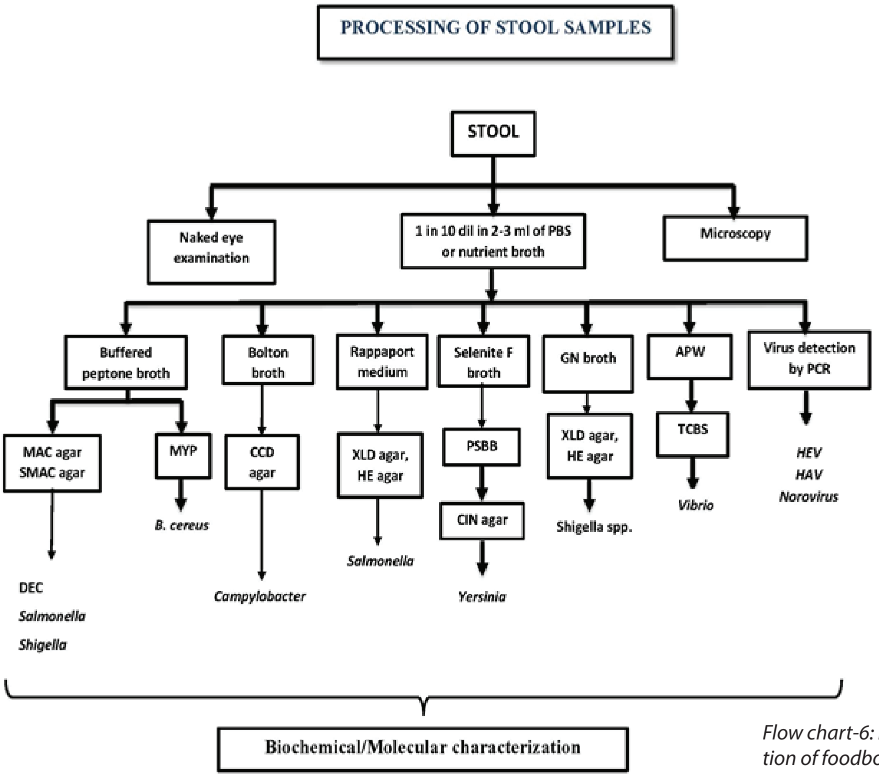

Flow chart-6: Processing of stool for isolation of foodborne pathogens

Procedure

1. The stool will be examined microscopically for consistency, presence of blood, or any helminth
2. A smear of stool will be also examined microscopically for parasitic ova or cyst.
3. For bacterial isolation, first the sample will be diluted 1-10 dilutions in 2-3ml PBS or nutrient broth.
4. For direct plating: Using an applicator swab, collect a small amount of feces. Collect from areas with visible blood or mucous, if present. The swabs are then to be rolled over the first quadrant of the MacConkey & Hektoen plate (or MacConkey & XLD plate). Then, using a sterile 1µl inoculating loop the plates are to be streaked for isolation. Insert a new, sterile swab into the sample and then drop the swab into the tube of Selenite F broth. Loosely cap the tube. Incubate all media at 36°C (+/- 1°C) in a non-CO2 incubator for 18-24 hrs (overnight). Following overnight (18-24 hrs) incubation, plates are examined for Salmonella-like or Shigella-like colonies:
5. One ml of the diluted sample shall be inoculated into each of the following broth for enrichment: buffered peptone broth, Selenite F broth, APW and half strength Fraser broth.
6. After overnight incubation at 37°C, 0.1 ml of the sample shall be inoculated from Buffered peptone broth to a plate of MAC, SMAC, CCD, and MYP for isolation of E.coli pathogroups, Salmonella, Shigella, Staphylococcusaureus, Campylobacter and Bacillus cereus,respectively.
7. Following 18-24 hrs of incubation, the growth from Selenite F broth is plated to HE (or XLD). The plates are incubated for 18-24 hrs at 37°C in a non-CO2 incubator for isolation of Salmonella and Shigella.
8. Following an incubation of 4~6hrs at 37°C for enrichment the growth from APW is subcultured on TCBS for isolation of vibrios.
9. The PSBB is incubated at 10°C for 10 days, after which it is streaked on CIN and MacConkey plates for isolation of Yersinia.
10. For isolation of Listeria, with 0.1 ml of pre-enriched Fraser broth content in 10 ml full strength Fraser broth containing selective supplements for 48 hrs at a 37°C incubation temperature will be done.The inoculum will be plated on PALCAM agar and incubated for 48 hrs at 37°C for isolation.
11. Virus will be detected by RT-PCR directly after extraction of RNA from the diluted sample.

PROCESSING OF ENVIRONMENTAL SAMPLES

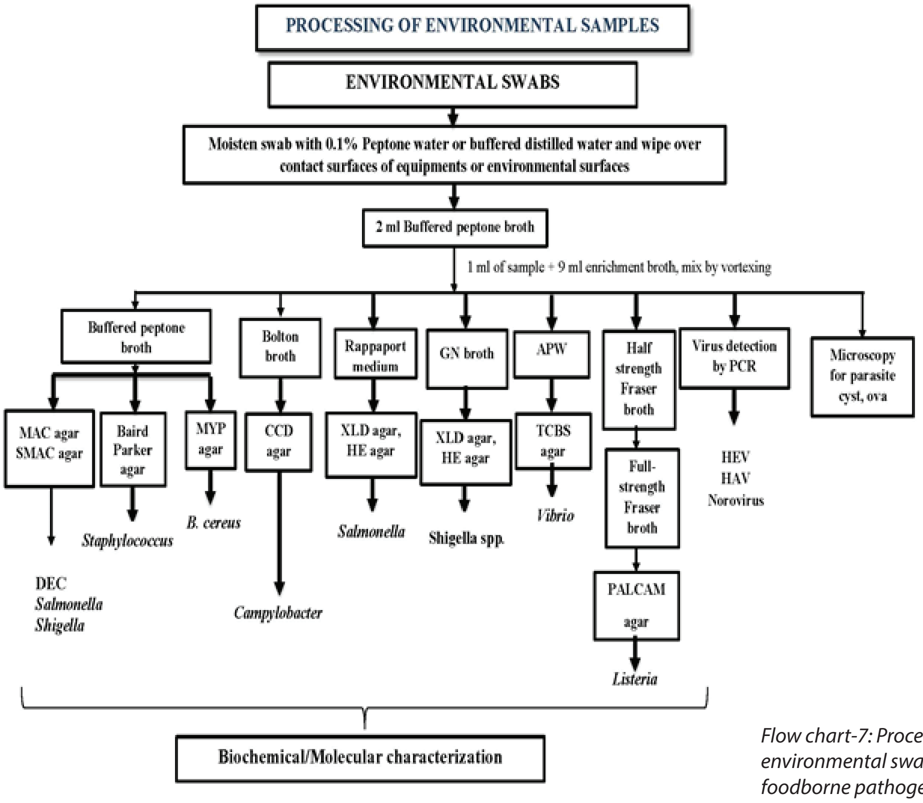

Flow chart-7: Processing of environmental swabs for isolation of foodborne pathogens

Procedure

1. Swab will be moistened with 0.1% peptone water or buffered distilled water and wiped over different surfaces.
2. The swab will be inoculated into 2ml of buffered peptone water. The sample will be carried to the laboratory.
3. One ml of the diluted sample shall be inoculated into each of the following broth (9 ml) for enrichment: buffered peptone broth for enrichment ofDEC, Salmonella, Shigella, B. cereus; Selenite F broth for enrichment of Shigella; Rappaport-Vassiliadis (RV) medium for enrichment of Salmonella; PSBB for enrichment of Yersinia; APW for enrichment of vibrios and half strength Fraser broth for enrichment of Listeria. Mix gently by vortexing.
4. After overnight incubation at 37°C, 0.1 ml of the sample shall be inoculated from Buffered peptone broth to a plate of MAC, SMAC, CCD, and MYP for isolation ofDEC, Salmonella, Shigella, Staphylococcus aureus, Campylobacter and Bacillus cereus respectively.
5. Following 18-24 hrs of incubation, the growth from Selenite F broth Rappaport-Vassiliadis (RV) medium is plated to HE (or XLD). The plates are incubated for 18-24 hrs at 37°C in a non-CO2 incubator for the isolation of Shigella and Salmonella.
6. Following an incubation of 4~6hrs at 37°C for enrichment the growth from APW is subcultured on TCBS for isolation of vibrios.
7. The PSBB is incubated at 10°C for 10 days, after which it is streaked on CIN and MacConkey plates for isolation of Yersinia.
8. For isolation of Listeria, with 0.1 ml of pre-enriched Fraser broth content in 10 ml full strength Fraser broth containing selective supplements for 48 hrs at a 37°C incubation temperature will be done.The inoculum will be plated on PALCAM agar and incubated for 48 hrs at 37°C for isolation.
9. Virus will be detected by RT-PCR directly after extraction of RNA from the diluted sample.
10. Parasitescan be detected directly from the diluted sample by microscopy following centrifugation

PROCESSING OF SKIN LESIONS, NASAL SWABS (Only during outbreak)

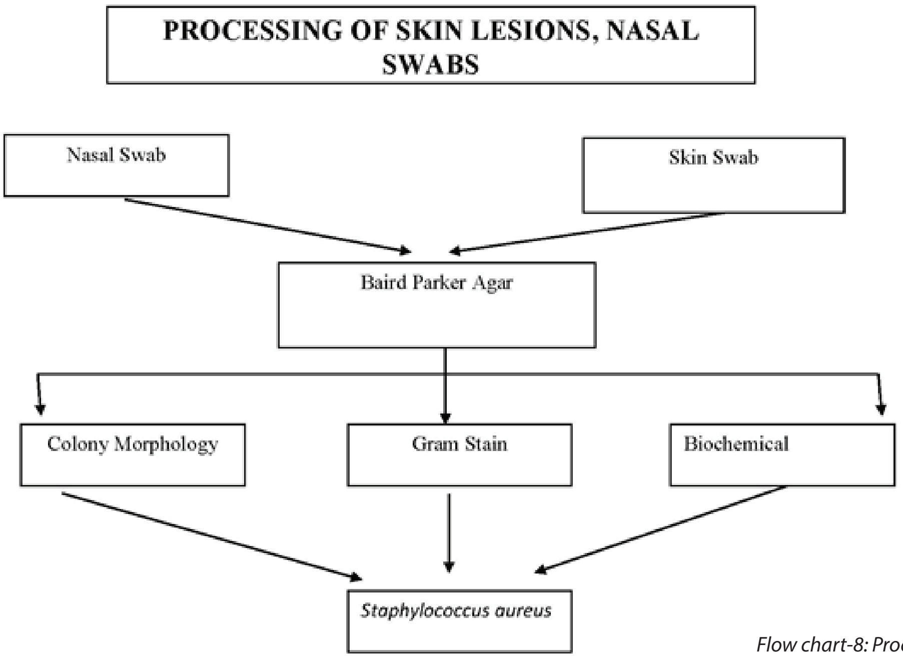

Flow chart-8: Processing of skin and nasal swabs from food handlers for isolation of foodborne pathogens

1. Nasal and skin swabs will be directly plated onto Baird parker agar plate and incubated overnight at 37°C.
2. Staphylococcusaureus will be identified based on colony morphology, Gram stain and biochemical tests.

5.4 PROCEDURE FOR ISOLATION OF PATHOGENS

SALMONELLA & SHIGELLA

Steps for isolation from stool

1. Using an applicator swab, collect a small amount of faeces. Collect from areas with visible blood or mucous, if present. The swabs are then to be rolled over the first quadrant of the MacConkey & Hektoen plate (or MacConkey & XLD plate).
2. Then, using a sterile 10 µL inoculation loop the plates are to be streaked for isolation.
3. Insert a new, sterile swab into the sample and then drop the swab into the tube of Selenite F broth. Loosely cap the tube. Incubate all media at 36°C (+/- 1°C) in a non-CO2 incubator for 18-24 hrs (overnight). Following overnight (18-24 hrs) incubation, plates are examined for Salmonella-like or Shigella-like colonies.
4. Insert a new, sterile swab into the sample and then drop the swab into the tube of Rappaport-Vassiliadis (RV) medium. Loosely cap the tube. Incubate all media at 36°C (+/- 1°C) in a non-CO2 incubator for 18-24 hrs (overnight). Following overnight (18-24 hrs) incubation, plates are examined for Salmonella-like or Shigella-like colonies.

	produce colourless (lactose negative)
**Hektoen Enteric Agar (HE)**	typically produces clear colonies with distinct black centres (hydrogen sulphide: H<br>ser. Typhi are typically clear with pinpoint black centres and<br>ser. Paratyphi A are typically clear (no H<br><br>typically produce colonies on HE, which range in colour from clear to white/pale
**Xylose Lysine Desoxycholate Agar (XLD)**	typically produces clear to light pink colonies with distinct black centres on XLD. Colonies of<br>typically clear with pinpoint black centres and colonies of<br>ser. Paratyphi A are typically clear (no H<br><br>typically produce colonies on XLD which range in colour from clear to white/pale

Broth Enrichment:

Following 18-24 hrs of incubation, the Selenite F broth and Rappaport-Vassiliadis (RV) medium is plated to HE (or XLD) using the same technique as above. The plates are incubated for 18-24 hrs at 36°C in a non-CO2 incubator.

Following overnight incubation, the plate is examined as described above,

Suspect colonies must be biochemically confirmed,

Steps for isolation from foods and water:

1. Mix the incubated Rappaport-Vassiliadis (RV) medium (in tube) by vortexing. Culture from the broth is plated to MacConkey, HE (or XLD) agar. The plates are incubated at 36°C (+/- 1°C) in a non-CO2 incubator for 18-24 hrs (overnight). Following overnight incubation, the plate is examined for Salmonella-like colonies as described above.

   Suspect colonies must be biochemically confirmed.
2. Mix the incubated Selenite F broth (in tube) by vortexing. Culture from the broth is plated onto MacConkey, HE (or XLD) agar. The plates are incubated at 36°C (+/- 1°C) in a non-CO2 incubator for 18-24 hrs (overnight). Following overnight incubation, the plate is examined Shigella-like colonies as described above.

   Suspect colonies must be biochemically confirmed.

Biochemical Identification:

Following 18-24 hrs of incubation, the Selenite F broth and Rappaport-Vassiliadis (RV) medium is plated to HE (or XLD) using the same technique as above. The plates are incubated for 18-24 hrs at 36°C in a non-CO2 incubator.

Biochemical Identification of Salmonella & Shigella (WHO-GFN laboratory protocol) (Figs. S1b and S2b)

Scheme for identification of Salmonella enterica

Isolate identification- Based on colony morphology and non-lactose fermentation, the isolatesare identified using standard biochemical tests. The Enterobacteriaceae with biochemical indicating Salmonella Typhi or Paratyphi A, B or C will be further confirmed by slide agglutinationtest using the Salmonella antisera.

Serologic identification of Shigella: rapid slide agglutination test

1. If the test for urease is negative on non-motile cultures with TSI reactions suggestive of Shigella species, we shall do rapid slide agglutination tests, with the appropriate sera, according to indole test and mannitol fermentation test results.
2. Shigella is divided based on mannitol fermentation

   Mannitol negative- Shigella dysenteriae (Group A)

   Mannitol positive- Shigella flexnerii(Group B)

   Shigella boydii (Group C)

   Shigella sonnei (Group D)
3. Group A (10 serotypes) is divided on the indole production into

   Indole negative serotypes 1, 3, 4, 5, 6, 9, 10

   Indole positive serotypes 2, 7, and 8
4. Groups B, C, D are based on lactose fermentation and indole production
5. Group D is the only late lactose fermenter; it doesn't produce indole and has only one serotype.
6. Between Group B (6 serotypes) and Group C (15 serotypes); only serotypes 6 of Group B is indole negative along with serotypes 1, 2, 3, 4, 6, 8, 10, 12, 14 of group C and serotypes 1-5 of group B with serotypes 5, 7, 9, 11, 13, 15 of group C are indole positive.
7. Suspend growth from cultures, which appear to be Shigella, but which do not react with Shigella antisera in saline, heat at 100°C for 30 min, and retest.

Isolation & identification of *Salmonella* & *Shigella* from food and environmental samples

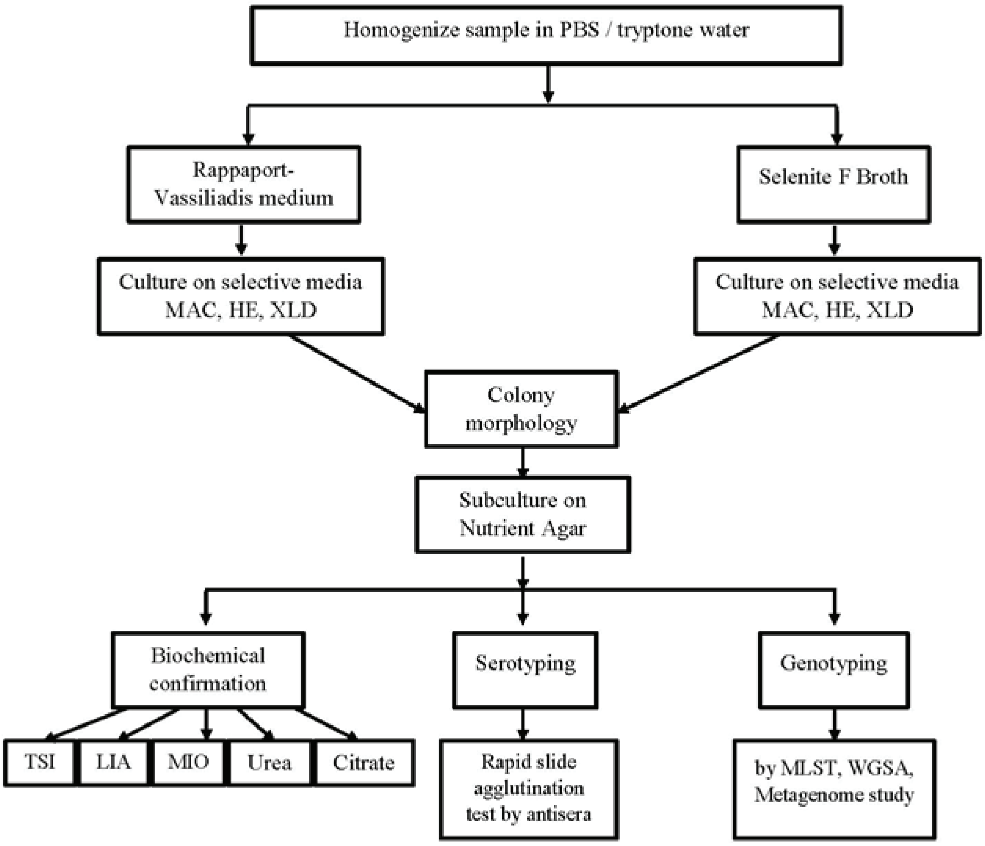

Flow Chart-9:Isolation & identification of Salmonella and Shigella from food & human faeces

CAMPYLOBACTER

Steps for isolation of thermotolerant Campylobacter from faeces, food or water

Procedure for faeces

Day 1: Selective enrichment with CCD agar plates
Pick faeces by a swab, and streak it onto CCD agar plate.

Incubate the plate at 42°C for 1-5 days under microaerophilic conditions.

Day 3: Spreading on Columbia agar plates containing 5% cattle blood
Characteristic growth from CCD-agar plates is transferred to a blood agar plate in a way that single colonies can be expected.

Incubate under microaerophilic conditions overnight at 42°C.

Procedure for food

Day 1: Enrichment in selective medium

Transfer 25gm 10 gm of food to a flask containing 225 ml 90 ml of Preston broth. Incubate the enrichment broth at 42°C for 24 to 48 hrs. The flask must be equipped with a cotton plug and incubated undermicroaerophilic conditions.

Day 2: Isolation on solid selective medium, CCD-agar

Using a 10µl loop, transfer material from the incubated enrichment broth toa CCD agar plate. Incubate under microaerophilic conditions at 42°C for 1-5 days.

Day 3: Spreading on Columbia agar platescontaining 5% sheep blood

Characteristic growth from CCD-agar plates is transferred to a blood agar plate in a way thatsingle colonies can be expected. Incubateunder microaerophilic conditions overnight at42°C.

Procedure for water

Day 1: Enrichment in selective medium

Transfer 10 ml of the water to a flask containing 90 ml of Preston broth or more in general to filtrate a portion of water, do not let filter become completely dry. Immediately transfer finished filter aseptically to broth using a sterile forceps. Enrich the filter in 10-25 ml Preston broth. Incubate the enrichment broth at 42°C for (24-) 48 hrs. The flask must be equipped with a cotton plug and incubated under microaerophilic conditions.

Day 2: Isolation on solid selective medium, CCD-agar

Using a 10 µl loop, transfer material from the incubated enrichment broth to a CCD agar plate. Incubate under microaerophilic conditions at 42°C for 1-5 days.

Day 3: Spreading on Columbia agar plates containing 5% sheep blood

Characteristic growth from CCD-agar plates is transferred to a blood agar plate in a way that single colonies can be expected. Incubate under microaerophilic conditions overnight at 42°C.

Identification

Biochemical characterization:

Generally, they are inert organisms. The morphology on Gram's stain is confirmatory.

Reactions are as follows.

Oxidase-positive, nitrate reduction positive, indole negative, urease negative, methylred negative, Voges-Proskauer negative.

Multiplex PCR for the detection of C. jejuni and C. coli

Shall be carried out using published primers

Primers

Primer Col1: 5' AGG CAA GGG AGC CTT TAA TC 3'

Primer Col2: 5' TAT CCC TAT CTA CAA ATT CGC 3'

Primer Jun3: 5' CAT CTT CCC TAG TCA AGC CT 3'

Primer Jun4: 5' AAG ATA TGG CAC TAG CAA GAC 3'

Procedure

1. One ml of PBS is transferred to a 1.5 ml Eppendorf tube. Using a disposable inoculation loop a loop full of bacteria is picked from a plate and transferred to the Eppendorf tube.
2. Centrifuge at 14,000 rpm for 5 min. Supernatant is discarded and the pellet is re-suspended in 100µl TE 10:1.
3. Boil the suspension (or heat at 95˚C) for 5-10 minutes and transfer directly to ice. Dilute the lysed DNA 10 fold in TE 10:1
4. Check the number of samples and calculate the amount of PCR master mix needed.
5. Prepare the PCR master mix in a tray of crushed ice.

6. Aliquot the PCR master mix to the required number of PCR tubes (24 µl per tube). Depending on the PCR machine that is used, one drop of mineral oil should be added.
7. Add 1 µl of water to the negative control tube and close the lid.
8. Add 1 µl of sample to the sample tube and close the lid.
9. Finish the procedure by adding the positive control DNA and close the lid.
10. Place the tubes into the PCR thermocycler
11. Program the PCR thermocycler
12. Run the program as given below

PCR Program

5 min. 94°C

1 min. 94°C, 1 min. 64°C, 1 min 72°C, 2 cycles

1 min. 94°C, 1 min. 62°C, 1 min 72°C, 2 cycles

1 min. 94°C, 1 min. 60°C, 1 min 72°C, 2 cycles

1 min. 94°C, 1 min. 58°C, 1 min 72°C, 2 cycles

1 min. 94°C, 1 min. 56°C, 1 min 72°C, 2 cycles

1 min. 94°C, 1 min. 54°C, 1 min 72°C, 30 cycles

10 min. 72°C

hold 4°C

Preparation of the agarose gel

1. Assemble the gel tray and make a proper set-up.

2. Prepare a 1% agarose solution by boiling the solution a few minutes till completely solved by the use of a water bath or microwave oven.

3. Cool the agarose to 40-50°C in a water bath and poor the agarose into the gel tray.

4. Let the gel solidify for 15-30 minutes.

5. Prepare a staining bath containing a final concentration of 5 ug/ml ethidium bromide.

Strains for Positive control

C. jejuni ATCC 29428: Positive control

C. coli ATCC 33559: Positive control

C. lari ATCC 35221: Negative control

Detection

Look for the presence of specific bands. For C. jejuni this should be 773 bp and for C. coli 364 bp.

Identification and characterization of *Campylobacter*

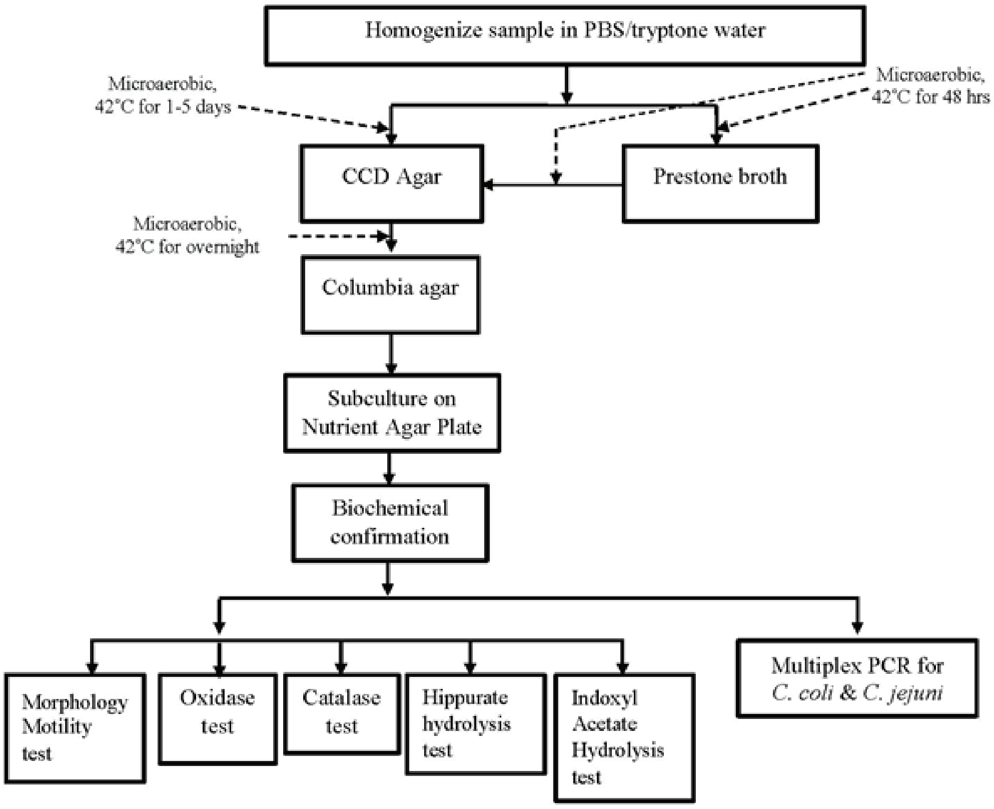

Flow chart-10: Isolation & identification of Campylobacter from food & human faeces

ESCHERICHIACOLIO157: H7

Steps for isolation and identification of Escherichia coliO157:H7 from faecesand food

Day 1: Enrichment
Shall be carried out in buffered peptone water

Spread on selective agar plates:

Spread-inoculate two plates of each dilution to Cefixime TelluriteSorbitol MacConkey (CT-SMAC agar) and one plate of MacConkey agar. Incubate at 37°C for 18 hrs. Typical growths in MaC and SMAC media are shown in Fig. S3a.

Day 2: Subculturing of presumptive colonies of Escherichia coli O157: H7.

Observe the CT-SMAC plates.

Isolate five presumptive sorbitol negative colonies from each agar plate and inoculate onto Nutrient agar plates. Incubate at 37°C for 18-24 hrs.

Escherichia coli O157:H7 on CT-SMAC plates. The colonies are transparent/colourless with a weak, pale brownish appearance because the bacterium does not ferment sorbitol (**Fig. S3a**).

Observe the MacConkey agar for otherDEC(Fig. S4a). Typical biochemical test results are shown in Figs. S3b and S4b.

Pick five Escherichia coli O157:H7 suspect colonies from the CT-SMAC agar plates and streak the colonies

onto non-selective medium, e.g. nutrient agar plates for biochemical confirmation and agglutination. Incubate the plates at 37°C for 18-24 hrs. Continue with the biochemical confirmation on pure cultures.

Day 3 & 4: Biochemical confirmation.

From a pure culture on nutrient agar plates we shall carry out the indole and other tests (Figs. S3b and S4b).

Escherichia coli O157:H7should then be agglutinated. If negative, test for the other major STEC serogroups (O26, O103, O111, and O145).

Conventional agglutination of Escherichia coliO157:H7

Shall be carried out using latex kit

MULTIPLEX PCR FOR THE DETECTION OF E. COLI PATHOTYPES (EAEC, EPEC, EHEC, ETEC, EIEC, DAEC)

**Primers / Reference**	**Size**
**DEC multiplex PCR**	**(Vidal et al., 2005)**
**EHEC**	
stx1: Forward 5′-CAG TTA ATG TGG TGG CGA AGG-3′	348 bp
Reverse 5′-CAC CAG ACA ATG TAA CCG CTG-3′	
stx2: Forward 5′-ATC CTA TTC CCG GGA GTT TAC G-3′	584 bp
Reverse 5′-GCG TCA TCG TAT ACA CAG GAG C-3′	
**EPEC**	
eae: Forward 5′-TCA ATG CAG TTC CGT TAT CAG TT-3′	482 bp
Reverse 5′-GTA AAG TCC GTT ACC CCA ACC TG-3′	
bfp: Forward 5′-GGA AGT CAA ATT CAT GGG GGT AT	300bp
Reverse 5′-GGA ATC AGA CGC AGA CTG GTA GT	
**ETEC**	
lt: Forward 5′-GCA CAC GGA GCT CCT CAG TC	218 bp
Reverse 5′-TCC TTC ATC CTT TCA ATG GCT TT	
stII: Forward 5′-AAA GGA GAG CTT CGT CAC ATT TT	129 bp
Reverse 5′-AAT GTC CGT CTT GCG TTA GGA C	
**EIEC**	
virF: Forward 5′-AGC TCA GGC AAT GAA ACT TTG AC	618 bp
Reverse 5′-TGG GCT TGA TAT TCC GAT AAG TC	
ipaH: Forward 5′-CTC GGC ACG TTT TAA TAG TCT GG	933 bp
Reverse 5′-GTG GAG AGC TGA AGT TTC TCT GC	
**DAEC**	
daaE: Forward 5′-GAA CGT TGG TTA ATG TGG GGT AA	542 bp
Reverse 5′-TAT TCA CCG GTC GGT TAT CAG T	
**EAEC**	
aafII: Forward 5′-CAC AGG CAA CTG AAA TAA GTC TGG	378 bp
Reverse 5′-ATT CCC ATG ATG TCA AGC ACT TC	

ETEC **(Osek, 2001)**

stal: ICRV 214 (In-house control)

Forward 5′-TCTTTCCCCTCTTTAGTCAG-3′ 166 bp

Reverse 5′-ACAGGCCGGATTACAACAAAG-3′

EAEC **(Muller et al., 2007)**

astA: ICRV 181(In-house control)

Forward 5′-TGCCATCAACACAGTATATCCG-3′ 102

Forward 5′-ACGGCTTTGTAGTCCTTCCAT-3′

EAEC multiplex PCR **(Cerna et al., 2003)**

aap: Forward 5′-CTT GGG TAT CAG CCT GAA TG-3′ 310 bp

Reverse 5′-AAC CCA TTC GGT TAG AGC AC-3′

aggR: Forward 5′-CTA ATT GTA CAA TCG ATG TA-3′ 457

Reverse 5′-AGA GTC CAT CTC TTT GAT AAG-3′

AA probe: Forward 5′-CTG GCG AAA GAC TGT ATC AT-3′ 629 bp

Reverse 5′-CAA TGT ATA GAA ATC CGC TGT T-3′

Procedure

1. One ml of BHI is transferred to a 1.5-ml Eppendorf tube. Using a disposable inoculation loop a loop full of bacteria is picked from a plate and transferred to the Eppendorf tube.
2. Centrifuge at 15,000 x g for 3 min.Supernatant is discarded and wash the pellet twice in physiological saline.
3. Resuspend the pellet in 100µl TE,boil the suspension (or heat at 95˚C) for 5-10 minutes and transfer directly to ice. Dilute the lysed DNA 10 fold in TE 10:1. Store at -20˚C.
4. Check the number of samples and calculate the amount of PCR master mix needed.
5. Prepare the PCR master mix in a tray of crushed ice
6. Aliquot the PCR master mix to the required number of PCR tubes (24 µl per tube). Depending on the PCR machine that is used.
7. Add 1 µl of water to the negative control tube and close the lid.
8. Add 1 µl of sample to the sample tube and close the lid.
9. Finish the procedure by adding the positive control DNA and close the lid.
10. Place the tubes into the PCR Thermocycler
11. Program the PCR Thermocycler
12. Run the program as given below

The PCR protocol:

DEC multiplex PCR thermal profile

5 min at 94°C,

1.5 min at 94°C,
1.5 min at 60°C,
1.5 min at 72°C, followed by a } 35 cycles of

final extension step at 72°C for 7 min.

hold at 4°C

ETEC PCR thermal profile

5 min at 94°C,

1 min at 94°C,
1 min at 60°C,
2 min at 72°C, } 30 cycles of

final extension step at 72°C for 5 min.

hold at 4°C

EAEC PCR thermal profile

5 min at 95°C,

45 Sec at 94°C,
45 Sec at 55°C,
45 Sec at 72°C, } 30 cycles of

final extension step at 72°C for 10 min and at hold at 4°C.

Detection

The multiplex PCR protocol will detect the 6 pathotypes E. coli and the presence of the virulence factors.

Flow diagram for isolation and identification of enterohaemorrhagic *Escherichia coli* O157 from food and faeces

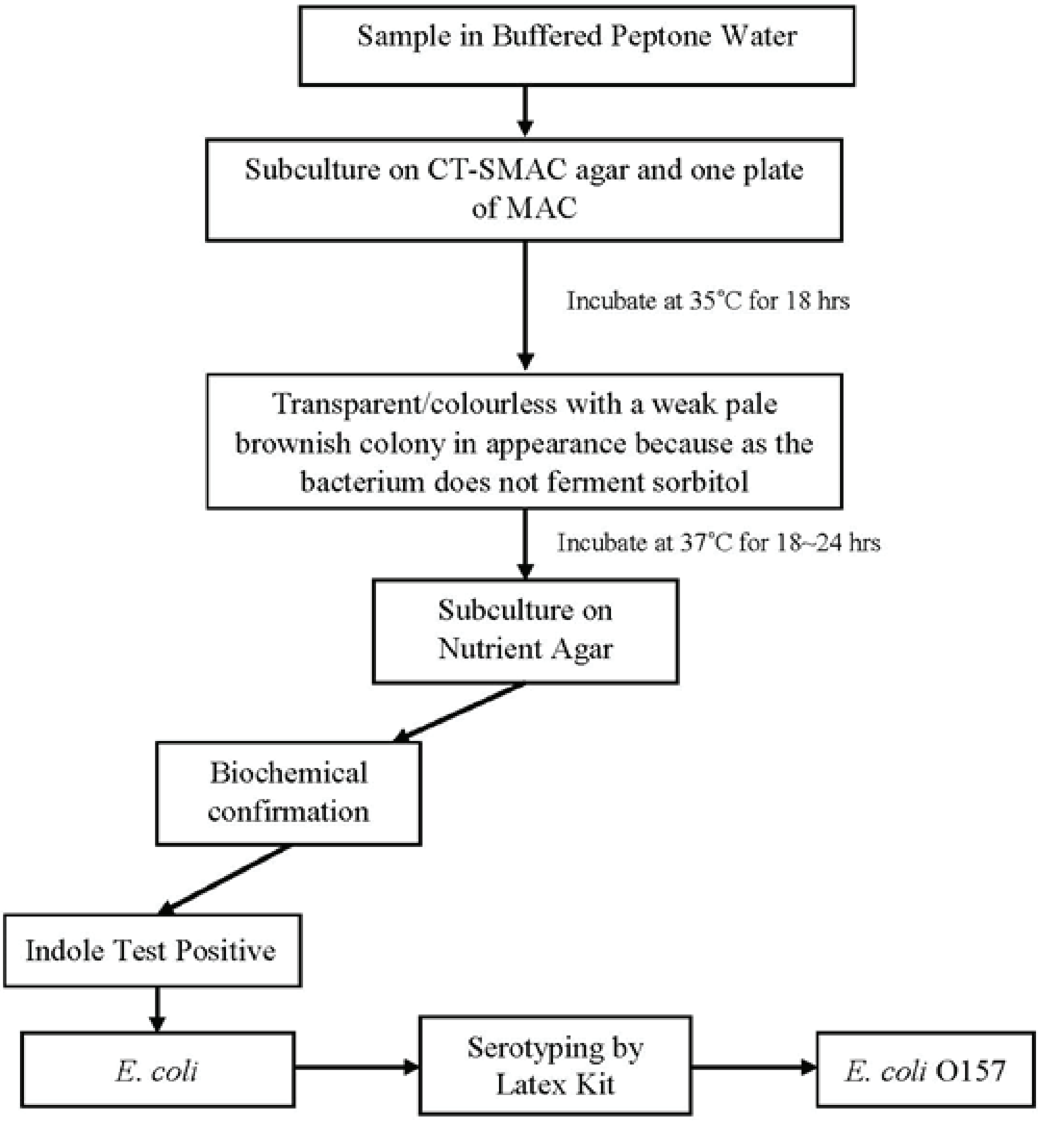

Flow Chart 11: Isolation and identification of enterohaemorrhagic *Escherichia coli* O157 from food and faeces

VIBRIO CHOLERAE

Identification of Vibrio cholerae O1 and O139 strains from clinical specimens as well as environmental sources.

Enrichment, isolation and presumptive identification of V. cholerae:

Procedure for processing faecal specimens

Isolation of V. cholerae O1/O139 from faecal specimens is accomplished with the use of highly selective media that suppress the growth of other organisms. Alkaline peptone water (APW, pH 8.0) is suggested as an enrichment broth, and thiosulfate citrate bile salts sucrose (TCBS) agar is the selective agar medium. APW enrichment for 4-6 hrs at 37°C and a selective plating medium on TCBS should always be used.

Procedure for processing water samples

For water samples, use the whole membrane for enrichment in 10-20 ml of APW. The membrane should be well immersed in the APW enrichment medium and incubated for 4-6 hrs at 37°C.

Using a sterile loop, a small portion from the surface of the enriched culture to be streaked onto TCBS agar plate. After overnight incubation at 37°C, observe the plates for the presence of well isolated 2~4 mm diameter umbilical shaped yellow coloured colony, which is the indicator for tentative presence of V. cholerae in the test samples (Fig. S5a).

Characterisation of presumptively identified V. cholerae:

Select well-separated yellow coloured colonies from TCBS plate (use five separated colonies for each sample) and inoculate on nutrient agar plates and incubate at 37°C for 18 hrs. Isolates that showed oxidase positivity are identified as V. cholerae.Typical biochemical test results are shown in Figs. S5b.

Serotyping of V. cholerae:

Cultures that were presumptively identified as V. cholerae will be tested for positivity in the oxidase test followed serological agglutination with polyvalent anti-O1 and anti-O139 sera. Positive agglutination with either O1 or O139 antisera signifies the presence of V. cholerae strains of the respective serogroup.

Strains that were identified as V. cholerae by the property of giving yellow coloured colony on TCBS, gave oxidase positivity, but not agglutinable with either of the anti-O1 or -O139 sera should be considered as V. cholerae strains belonging to non-O1, non-O139 serogroups.

Strains that were identified, as O1 can be further tested with monoclonal anti-Ogawa or anti-Inaba sera for placing these strains in either of the serotype based on their agglutination result.

Interpretation:

Isolates that produced yellow coloured colony on TCBS and showed positive oxidase reaction should be tested for serological character.

V. cholerae strains do not produce gas, so any indication related to gas production to be considered as the presence of observation.

Interpretation should be based on the results obtained from serological tests and to confirm the presence or absence of V. cholerae strains belonging to either O1 or O139 serogroups.

Flow diagram for Isolation and Identification *Vibrio Cholerae /Vibrio parahaemolyticus* from stool sample

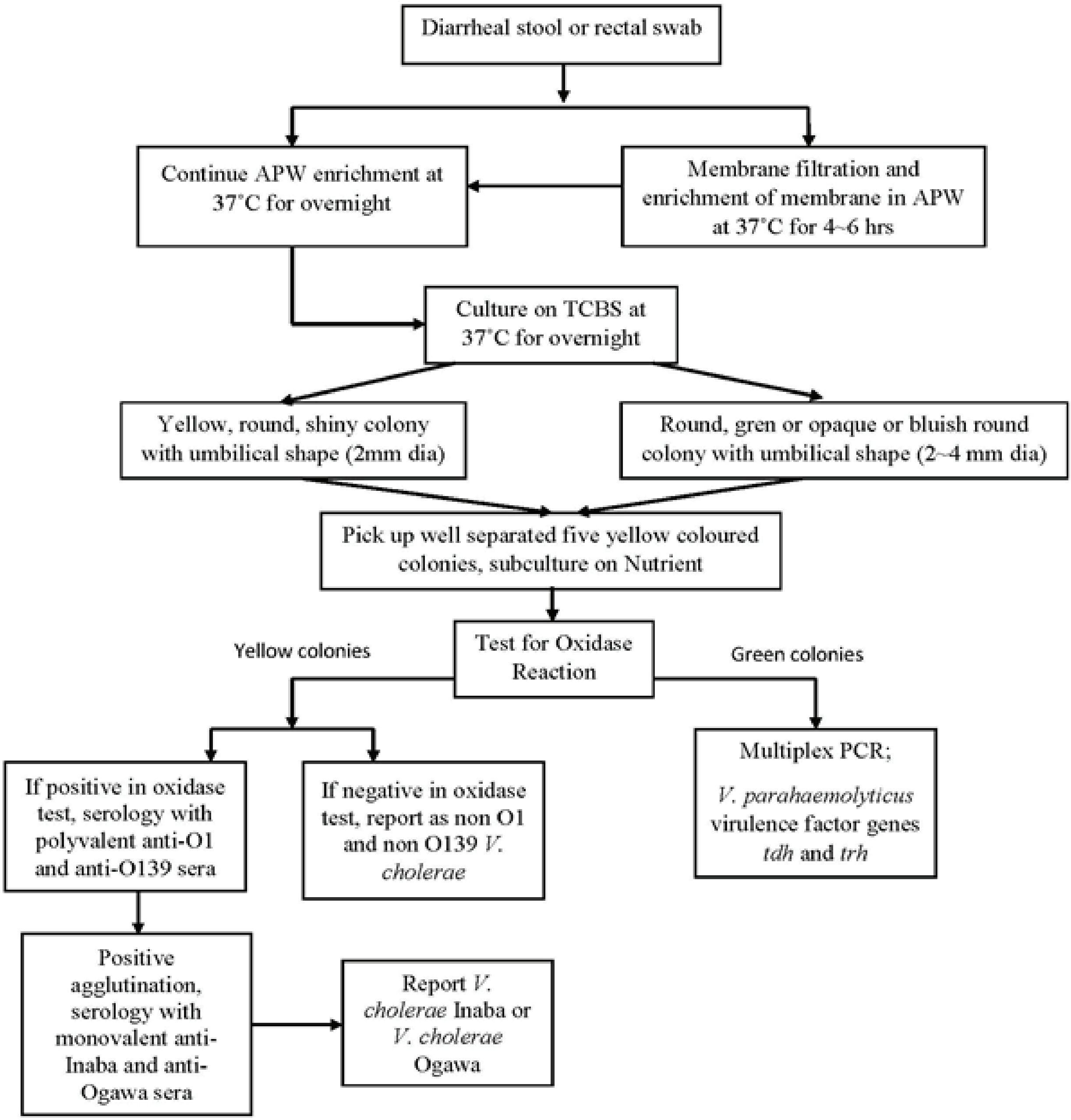

Flow Chart 12: Isolation and Identification Vibrio Cholerae /Vibrio parahaemolyticus from stool sample

Isolation and Identification *Vibrio Cholerae /Vibrio parahaemolyticus* from Water sample

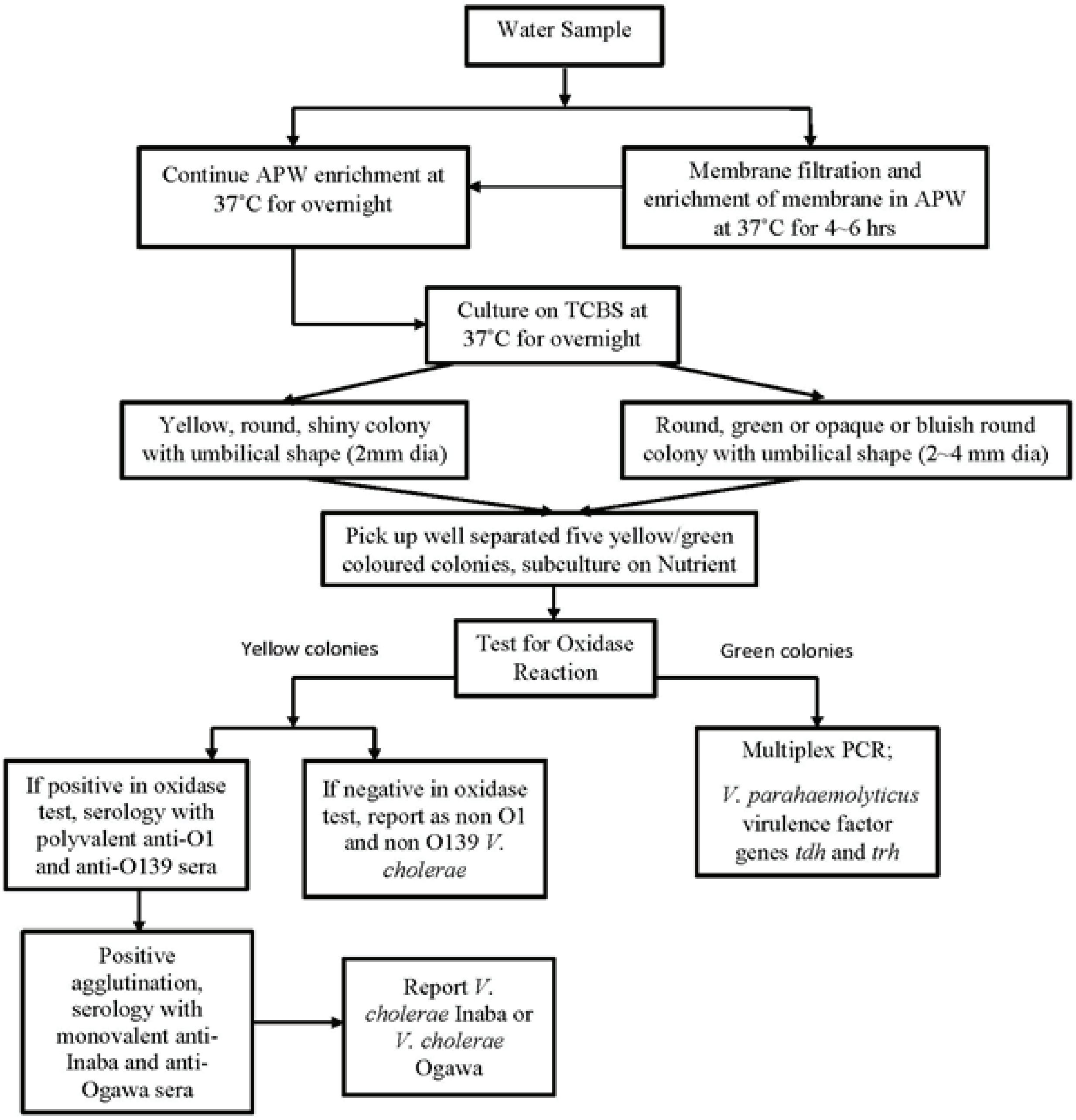

Flow Chart 13: Isolation and Identification Vibrio Cholerae /Vibrio parahaemolyticus from Water sample

Detection of Cholera toxin gene (ctx gene) in V. cholerae

A genotypic assay such as PCR amplification of the ctx gene is recommended.

Shall be carried out using published primers

Primers

Primer ctx F: 5'TGA AAT AAA GCA GTC AGG TG3'

Primer ctx R: 5' GGT ATT CTG CAC ACA AAT CAG 3'

Procedure

1. One ml of APW is transferred to a 1.5-ml Eppendorf tube. Using a disposable inoculation loop a loop full of bacteria is picked from a plate and transferred to the Eppendorf tube.
2. Boil the suspension (or heat at 95˚C) for 5-10 minutes and transfer directly to ice. Dilute the lysed DNA 10 fold in TE 10:1. Store at -20˚C.
3. Check the number of samples and calculate the amount of PCR master mix needed.
4. Prepare the PCR master mix in a tray of crushed ice
5. Aliquot the PCR master mix to the required number of PCR tubes (24 µl per tube). Depending on the PCR machine that is used.
6. Add 1 µl of water to the negative control tube and close the lid.
7. Add 1 µl of sample to the sample tube and close the lid.
8. Finish the procedure by adding the positive control DNA and close the lid.
9. Place the tubes into the PCR thermocycler
10. Program the PCR thermocycler
11. Run the program as given below

PCR Program

3 min. 94°C

1 min. 94°C	35 Cycles
1 min. 55°C	
2 min. 72°C	

3 min. 72°C

hold 4°C

Preparation of the agarose gel

1. Assemble the gel tray and make a proper set-up.
2. Prepare a 1% agarose solution by boiling the solution a few minutes till completely solved by the use of a water bath or microwave oven.
3. Cool the agarose to 40-50°C in a water bath and poor the agarose into the gel tray.
4. Let the gel solidify for 15-30 minutes.
5. Prepare a staining bath containing a final concentration of 5 µg/ml ethidium bromide.

Detection

Look for the presence of specific bands, the amplifiedctx geneshould be 777 bp.

VIBRIO PARAHAEMOLYTICUS

Enrichment, isolation and presumptive identification of V. parahaemolyticus:

A loop-full of diarrhoeal stool or the swab stick containing clinical specimen will be used to inoculate APW broth for selective enrichment of V. parahaemolyticus. Allow an incubation of 4~6 hrs at 37°C for enrichment.

Using a sterile loop, a small portion from the surface of the enriched culture to be streaked onto TCBS agar plate. After overnight incubation at 37°C, observe the plates for the presence of well-isolated 2~4 mm diameter umbilical shaped green or opaque, or bluish coloured colony, which is the indicator for the tentative presence of V. parahaemolyticus (Fig. S5a).

Characterization of presumptively identified V. parahaemolyticus

Pickup well separated green coloured colony from TCBS plate (use five separated colonies for each sample) to inoculate on nutrient agar plate and incubate at 37°C for 18hrs

Isolates that showed oxidase positivity,presumptively to be identified as V. parahaemolyticus.Typical biochemical test results are shown in Fig. S5b.

Serotyping of V. parahaemolyticus

Cultures that were presumptively identified as V. parahaemolyticus will be tested for positivity in the oxidase test and send to NICED, Kolkata for serotyping.

Multiplex PCR for the detection of V. parahaemolyticus virulence factors by amplification of tdh and trh genes (BAM protocol)

Shall be carried out using published primers

Primers

Primer L-TL: 5' AAA GCG GAT TAT GCA GAA GCA CTG 3'

Primer R-TL: 5' GCT ACT TTC TAG CAT TTT CTC TGC 3'

Primer VPTRH-L: 5' TTG GCT TCG ATA TTT TCA GTA TCT 3'

Primer VPTRH-R: 5' CAT AAC AAA CAT ATG CCC ATT TCC G 3'

Primer VPTDH-L: 5' GTA AAG GTC TCT GAC TTT TGG AC 3'

Primer VPTDH-R: 5' TGG AAT AGA ACC TTC ATC TTC ACC 3'

Procedure

1. One ml of PBS is transferred to a 1.5 ml Eppendorf tube. Using a disposable inoculation loop a loop full of bacteria is picked from a plate and transferred to the Eppendorf tube.
2. Centrifuge at 15,000 x g for 3 min. Supernatant is discarded and wash the pellet twice in physiological saline.
3. Resuspend the pellet in 100µl TE, boil the suspension (or heat at 95˚C) for 5-10 minutes and transfer directly to ice. Dilute the lysed DNA 10 fold in TE 10:1. Store at -20˚C.
4. Check the number of samples and calculate the amount of PCR master mix needed.
5. Prepare the PCR master mix in a tray of crushed ice
6. Aliquot the PCR master mix to the required number of PCR tubes (24 µl per tube). Depending on the PCR machine that is used.
7. Add 1 µl of water to the negative control tube and close the lid.
8. Add 1 µl of sample to the sample tube and close the lid.
9. Finish the procedure by adding the positive control DNA and close the lid.

10. Place the tubes into the PCR thermocycler
11. Program the PCR thermocycler
12. Run the program as given below

PCR Program

3 min. 94°C

1 min. 94°C

1 min. 60°C 25 Cycles

2 min. 72°C

3 min. 72°C

hold 4°C

Preparation of the agarose gel

1. Assemble the gel tray and make a proper set-up.
2. Prepare a 1% agarose solution by boiling the solution a few minutes till completely solved by the use of a water bath or microwave oven.
3. Cool the agarose to 40-50°C in a water bath and poor the agarose into the gel tray.
4. Let the gel solidify for 15-30 minutes.
5. Prepare a staining bath containing a final concentration of 5 µg/ml ethidium bromide.

Detection

Look for the presence of specific bands, the amplifiedtrh, tdh and tlh geneshould be 500 bp, 270 bp and 450 bp respectively.

LISTERIA MONOCYTOGENES

Isolation and identification of *Listeria* spp

Pre-enrichment:

1. Shall be done in half strength Fraser broth containing selective supplements for 24 hrs at 30°C.

Second enrichment

- Add 0.1 ml of pre-enriched Fraser broth content in 10 ml full strength Fraser broth containing selective supplements and incubate for 48 hrs at 37°C.

2. The inoculum will be plated on PALCAM agar and incubated for 48 hrs at 37°C.
3. The gray-green colonies surrounded by diffuse black zone on PALCAM agar will be picked up and further purification on Tryptone Soya Yeast Extract agar (TSYEA) will be done.
4. The pinpoint colonies in TSYEA will be subjected for Gram staining to look for positive, coccobacillary or short rod-shaped bacterial cells. Typical colony morphology in PALCAM medium and biochemical test results are shown in Figs. S6.
5. Catalase test and oxidase test will be done. Colonies showing catalase positive and oxidase negative will be subcultured in Brain-Heart Infusion broth at 25°C for 12-18 hrs.
6. Motility test can be done by hanging drop preparation. Cultures showing typical tumbling motility will be considered as "presumptive" listeria isolates.

Detection of Listeria monocytogenes virulence-associated gene by PCR

- The L. monocytogenes isolates will be screened for the presence or absence of virulence-associated gene(s) by using the standard PCR protocols for the detection of actA, hlyA, and iapgenes.

Primers:

Gene	Primer sequence	Base pair
act A	F: CGC CGC GGA AAT TAA AAA AAG<br>R: ACG AAG GAA CCG GGC TGC TAG	839 bp
hly A	F: GCA GTT GCA AGC GCT TGG AGT GAA<br>R: GCA ACG TAT CCT CCA GAG TGA TCG	456 bp
iap	F: ACA AGC TGC ACC TGT TGC AG<br>R: TGA CAG CGTGTG TAG TAG CA	131 bp

The PCR protocol:

Initial denaturation will be carried out at 95°C for 2 min followed by denaturation 95°C for 15 sec followed by annealing 60°C for 30 s and extension at 72°C for 1 min 30 sec and repeated for 35 cycles.

Isolation and identification of Listeria monocytogenes from faeces and food

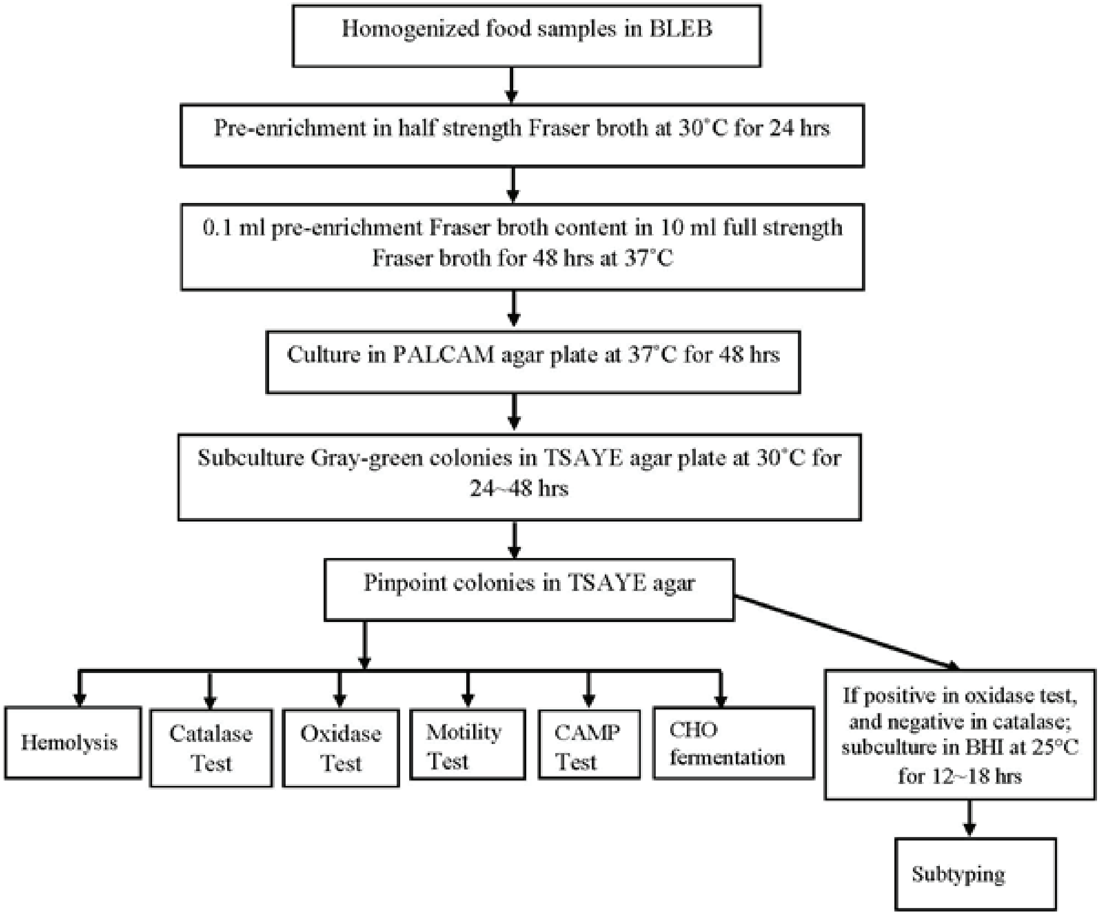

Flow Chart 14: Isolation and identification of *Listeria monocytogenes* from faeces and food

BACILLUS CEREUS

Procedure of isolation

- Serial dilutions will be prepared, and 2 µl of each diluted sample will be streaked in MYP agar medium.
- Plates will be incubated for 24 hrs at 30°C.
- Typical colonies of pink-orange, surrounded by a zone of precipitation indicating lecithinase production (Fig. S7)
- These colonies will be presumptively identified to be B. cereus.Typical biochemical test results are shown in Fig. S7.
- From each sample, a typical colony presumed to belong to the B. cereus group will be subcultured on brain heart infusion-yeast extract (BHI-YE) agar and incubated for 24 hrs at 30°C.

Molecular identification

1. Detection of *Bacillus cereus group*

Procedure

- B. cereus-like isolates will be grown overnight at 30°C on brain heart infusion-yeast extract (BHI-YE)
- Genomic DNA will be extracted by using commercially available bacterial DNA isolation kit.
- Spore structural protein gene (sspE) sequence specific to the B. cereus group will be targeted for PCR amplification using published primers.

Primers		Base pair	Reference
Forward: (5'-GAAAAAGATGAGTAAAAAACAACAA-3')	71bp		(Kim et al., 2005)
Reverse: (5'-CATTTGTGCTTTGAATGCTAG-3')			

The PCR protocol:

4min at 95°C,

30 s at 95°C,

30 sec at 59°C,30 cycles of

1min at 72°C, followed by a

final extension step at 72°C for 7 min.

hold at 4°C

Detection

Look for the presence of specific bands, the amplifiedsspE geneshould be 71 bp.

2. Detection of Emetic strains of B. cereus

Method-1

Primers	Base pair	Reference
CER1: ATCATAAAGGTGCGAACAAGA	188 bp	(Horwood et al., 2004)
EMT1: AAGATCAACCGAATGCAACTG		

Procedure

1. Check the number of samples and calculate the amount of PCR master mix needed.
2. Prepare the PCR master mix in a tray of crushed ice
3. Aliquot the PCR master mix to the required number of PCR tubes (24 µl per tube). Depending on the PCR machine that is used

4. Add 1 µl of water to the negative control tube and close the lid
5. Add 1 µl of sample to the sample tube and close the lid
6. Finish the procedure by adding the positive control DNA and close the lid.
7. Place the tubes into the PCR thermocycler
8. Program the PCR thermocycler
9. Run the program as given below

PCR Program

10 min. 94°C

1 min. 94°C
1 min. 52°C } 35 Cycles
1 min. 72°C

7 min. 72°C

hold 4°C

Preparation of the agarose gel

1. Assemble the gel tray and make a proper set-up.
2. Prepare a 1% agarose solution by boiling the solution a few minutes till completely solved by the use of a water bath or microwave oven.
3. Cool the agarose to 40-50°C in a water bath and poor the agarose into the gel tray.
4. Let the gel solidify for 15-30 minutes.
5. Prepare a staining bath containing a final concentration of 5 µg/ml ethidium bromide.

Detection

Look for the presence of specific bands, a 188 bp amplicon confirms the presence of emetic strains of Bacillus cereus.

OR

Method-2

Primers	Base pair	Reference
EM1F: 5'-GACAAGAGAAATTTCTACGAGCAAGTACAAT-3'	635 bp	(Ehling-Schulzetal., 2004)
EM1R: 5'-GCAGCCTTCCAATTACTCCTTCTGCCACAGT-3'		

Procedure

1. Check the number of samples and calculate the amount of PCR master mix needed.
2. Prepare the PCR master mix in a tray of crushed ice
3. Aliquot the PCR master mix to the required number of PCR tubes (24 µl per tube). Depending on the PCR machine that is used.
4. Add 1 µl of water to the negative control tube and close the lid
5. Add 1 µl of sample to the sample tube and close the lid
6. Finish the procedure by adding the positive control DNA and close the lid
7. Place the tubes into the PCR thermocycler
8. Program the PCR thermocycler
9. Run the program as given below

PCR Program

15 min. 95°C

30 sec 95°C
30 sec 60°C
1 min. 72°C
} 30 Cycles

5 min. 72°C

hold 4°C

Preparation of the agarose gel

1. Assemble the gel tray and make a proper set-up
2. Prepare a 1% agarose solution by boiling the solution a few minutes till completely solved by the use of a water bath or microwave oven
3. Cool the agarose to 40-50°C in a water bath and poor the agarose into the gel tray
4. Let the gel solidify for 15-30 minutes
5. Prepare a staining bath containing a final concentration of 5 µg/ml ethidium bromide

Detection

Look for the presence of specific bands, a 635 bp amplicon confirms the presence of emetic strains of Bacillus cereus.

Isolation and identification of *Bacillus cereus* from food like rice

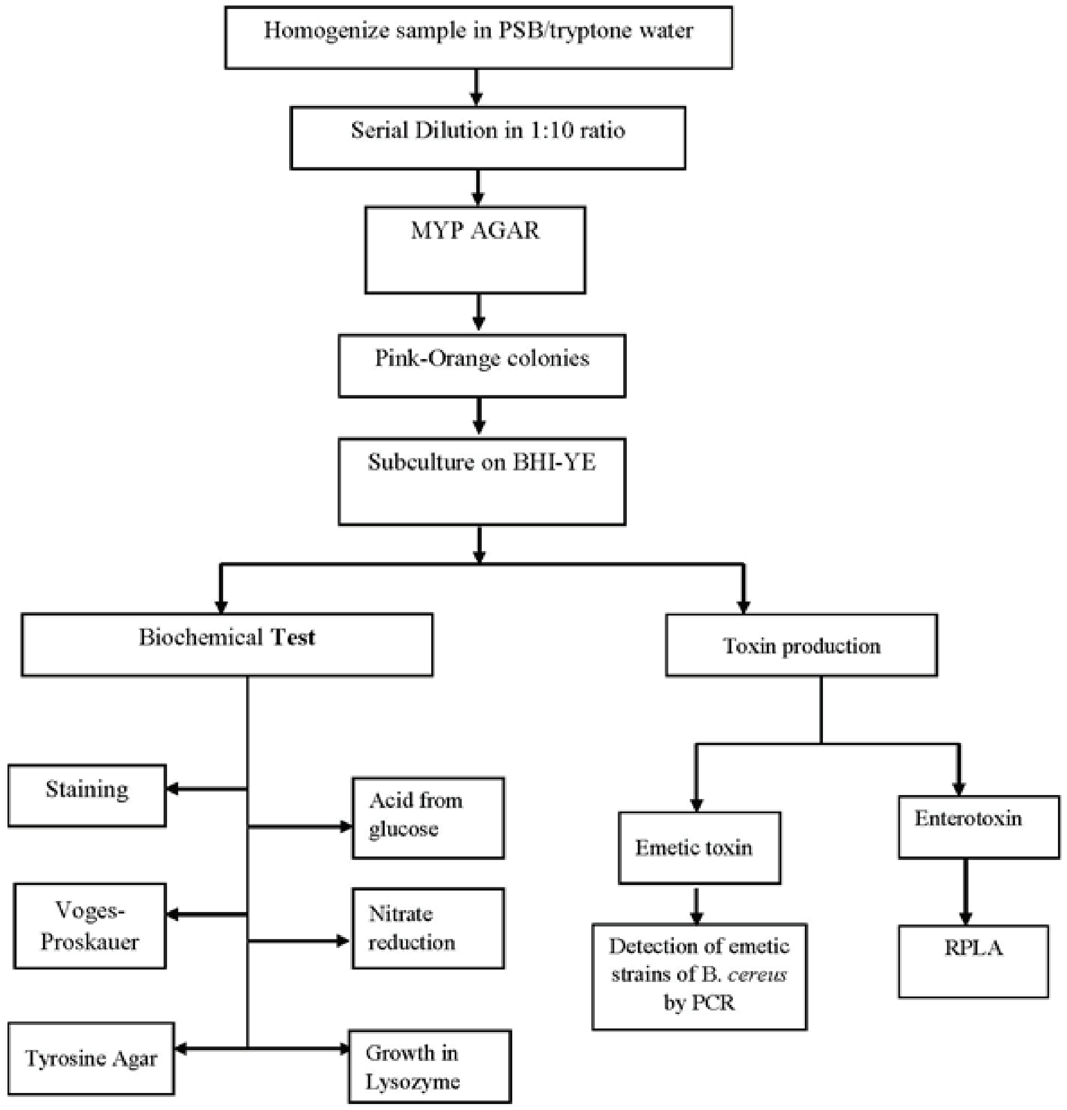

Flow Chart 15: Isolation and identification of *Bacillus cereus* from food like rice

STAPHYLOCOCCUS AUREUS

Steps for isolation and identification of Staphylococcus aureus from food samples (routine surveillance), nasal swabs and skin lesions (Outbreak investigation)

Enrichment

For food samples enrichment shall be carried out in Peptone Water (PW) culture on selective media.

The selective medium used for isolation of S. aureus will be Baird Parker Agar (BPA). A loopful of inoculum from enrichment media will be streaked on Baird-Parker agar and incubated for 48 hours at 37°C.

Identification

Characteristic appearance of jetblack colonies surrounded by a white halo will be presumptively identified as Staphylococcus aureus (Fig. S8).

Subculture shall be done on nutrient agar for biochemical characterization. Typical biochemical test results are shown in Fig. S8.

Biochemical identification

The following tests shall be carried out-

A. Additional Tests

(i) Gram staining for Gram-positive cocci in cluster

(ii) Catalase test: S. aureus will be positive

(iii) Anaerobic utilization of glucose: Acid production anaerobically and change in the colour toyellow throughout the tube confirms the presence S. aureus

(iv) Anaerobic utilization of mannitol: Acid production anaerobically and change in the colour to yellow confirms the presence S. aureus

(v) Lysostaphin sensitivity test: S. aureus is generally positive

B. Coagulase Test

S. aureus will be both tube and slide coagulase positive

Isolation and identification of *Staphylococcus aureus* from faeces and food

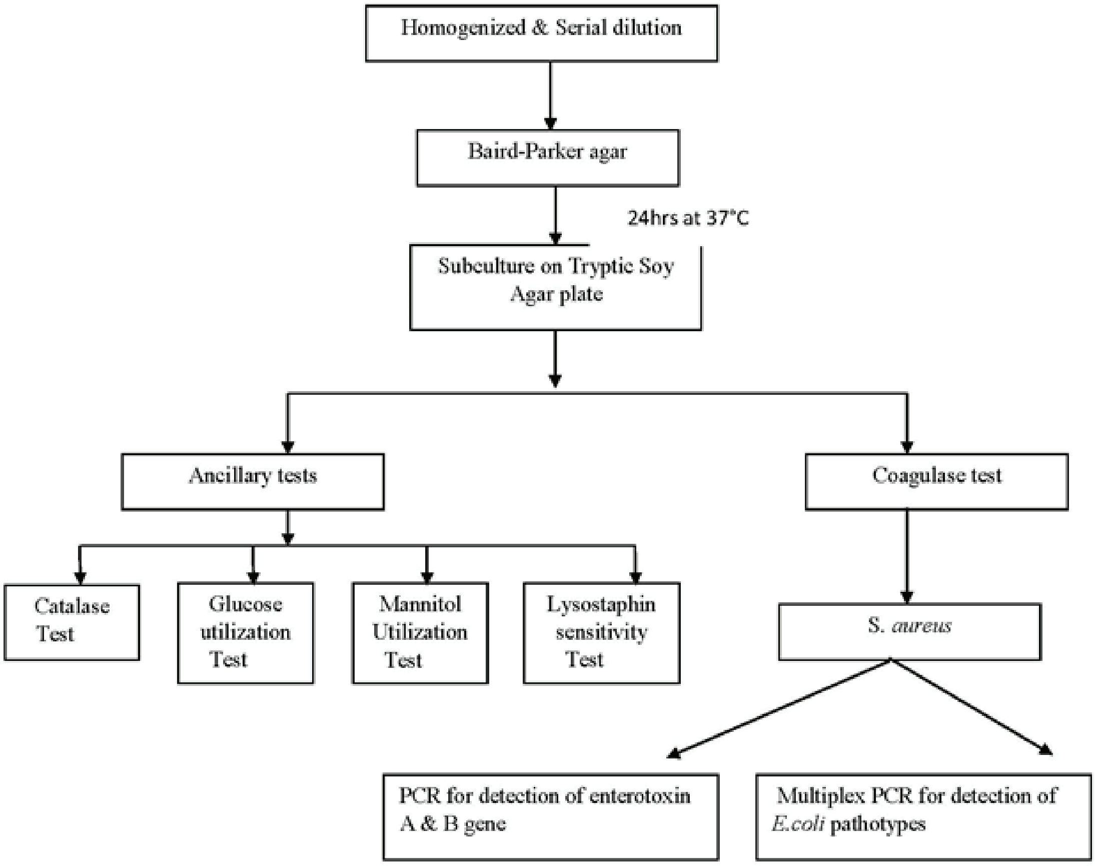

Flow Chart 16: Isolation and identification of *Staphylococcus aureus* from faeces and food

Multiplex PCR for the detection of genes encoding staphylococcal enterotoxins A to E

*P*rimers	Reference
Primer GSEAR-1: 5' GGT TAT CAA TGT GCG GGT GG 3'	Mehrotra et al., 2000
Primer GSEAR-2: 5' CGG CAC TTT TTT CTC TTC GG 3'	
Primer GSEBR-1: 5' GTA TGG TGG TGT AAC TGA GC 3'	
Primer GSEBR-2: 5' CCA AAT AGT GAC GAG TTA GG 3'	
Primer GSECR-1: 5' AGA TGA AGT AGT TGA TGT GTA TGG 3'	
Primer GSECR-2: 5' CAC ACTT TTA GAA TCA ACC G 3'	
Primer GSEDR-1: 5' CCA ATA ATA GGA GAA AAT AAA AG 3'	
Primer GSEDR-2: 5' ATT GGT ATT TTT TTT CGT TC 3'	
Primer GSEER-1: 5' AGGT TTT TTC ACA GGT CAT CC 3'	
Primer GSEDR-2: 5' CTT TTT TTT CTTC GGT CAA TC 3'	

Procedure

1. One ml of BHI is transferred to a 1.5-ml Eppendorf tube. Using a disposable inoculation loop a loop full of bacteria is picked from a plate and transferred to the Eppendorf tube
2. Centrifuge at 15,000 x g for 3 min.Supernatant is discarded and wash the pellet twice in physiological saline
3. Resuspend the pellet in 100µl TE,boil the suspension (or heat at 95˚C) for 5-10 minutes and transfer directly to ice. Dilute the lysed DNA 10 fold in TE 10:1. Store at -20˚C
4. Check the number of samples and calculate the amount of PCR master mix needed.
5. Prepare the PCR master mix in a tray of crushed ice
6. Aliquot the PCR master mix to the required number of PCR tubes (24 µl per tube). Depending on the PCR machine that is used
7. Add 1 µl of water to the negative control tube and close the lid
8. Add 1 µl of sample to the sample tube and close the lid
9. Finish the procedure by adding the positive control DNA and close the lid
10. Place the tubes into the PCR thermocycler
11. Program the PCR thermocycler
12. Run the program as given below

The PCR protocol:

5 min at 94°C,

2 min at 94°C,
2 min at 59°C,
1min at 72°C, followed by a } 35 cycles of

final extension step at 72°C for 7 min.

hold at 4°C

Preparation of the agarose gel

1. Assemble the gel tray and make a proper set-up.
2. Prepare a 1% agarose solution by boiling the solution a few minutes till completely solved by the use of a water bath or microwave oven.

3. Cool the agarose to 40-50°C in a water bath and poor the agarose into the gel tray.
4. Let the gel solidify for 15-30 minutes.
5. Prepare a staining bath containing a final concentration of 5 µg/ml ethidium bromide.

Detection

Look for the presence of specific bands, the amplifiedsea, seb, sec, sed, and see geneshould be 102 bp, 164 bp and 451 bp, 278 bp and 209 bp respectively.

YERSINIA ENTEROCOLITICA

Sample processing

- Analyze samples promptly after receipt, or refrigerate at 4°C. (Freezing of samples before analysis is not recommended, although Yersinia have been recovered from frozen products).
- Aseptically weigh 25 gms sample into 225 ml PSBB. Homogenize 30 sec and incubate at 10°C for 10 days.

Enrichment

- If high levels of Yersinia are suspected in product, spread-plate 0.1 ml on MacConkey agar and 0.1 ml on CIN agar before incubating broth.
- Also, transfer 1 ml homogenate to 9 ml 0.5% KOH in 0.5% saline, mix for 2-3 sec, and spread-plate 0.1 ml on MacConkey and CIN agars. Incubate agar plates at 30°C for 1-2 days.
- On day 10, remove enrichment broth from the incubator and mix well. Transfer one loop-full of enrichment to 0.1 ml 0.5% KOH in 0.5% saline and mix for 2-3 sec. Successively streak one loopful to MacConkey plate and one loopful to CIN plate.
- Transfer additional 0.1 ml enrichment to 1 ml 0.5% saline and mix 5-10 sec before streaking, as above. Incubate agar plates at 30°C for 1-2 days.

Isolation of Yersinia

- Examine CIN plates after incubation for one day. Select small (1-2 mm diameter) colonies having a deep red center with sharp border surrounded by clear colorless zone with entire edge (Fig. S9).
- Examine MacConkey agar plates after 1 to 2 days incubation. Select small (1-2 mm diameter) flat, colorless, or pale pink colonies.
- Inoculate each selected colony into LAIA slant, Christensen's urea agar plate or slant, and bile esculin agar plate or slant by stabbing with inoculation needle.
- Incubate 48 h at RT. Isolates giving alkaline slant and acid butt, no gas and no H2S (KA-) reaction in LAIA, which are also urease-positive, are presumptive Yersinia. Discard cultures that produce H2S and/or any gas in LAIA or are urease-negative. Give preference to typical isolates that fail to hydrolyze (blacken) esculin.
- Using growth from LAIA slant, streak culture to one plate of TSAYE and incubate at RT. Use growth on TSAYE to check for culture purity and do the oxidase test and Gram stain. Typical biochemical test results are shown in Fig. S9.

Biochemical tests for biotyping

From colonies on TSAYE, inoculate an agar medium containing egg yolk such as Anaerobic egg yolk (AEY) agar for lipase reaction (at 2-5 days, incubated aerobically at RT). Also, inoculate the following biochemical test media and incubate all at RT for 3 days (except one motility test medium and one MR-VP broth, which are incubated at 35-37°C for 24 hrs).

1. Fermentation of salicin, xylose and trehalose
2. VP reaction: Add 0.6 ml α-naphthol and shake well. Add 0.2 ml 40% KOH solution with creatine and shake. Read results after 4 hrs. Development of pink-to-ruby red color in medium is a positive test.
3. Lipase reaction: From colonies on TSAYE, inoculate an agar medium containing egg yolk such as Anaerobic egg yolk (AEY) agar for lipase reaction (at 2-5 days, incubated aerobically at RT).

4. Esculin

5. β-D-glucosidase test: Add 0.1 g 4-nitrophenyl-β-D-glucopyranoside to 100 ml 0.666 M NaH2PO4 (pH 6). Dissolve; filter-sterilize. Emulsify culture in physiologic saline to McFarland Turbidity Standard No. 3. Add 0.75 ml of culture to 0.25 ml of test medium. Incubate at 30°C overnight. A distinct yellow color indicates a positive reaction.

6. **Pyrazinamidase test:** After growth of culture on slanted pyrazinamidase agar at RT, flood 1 ml of 1% freshly prepared ferrous ammonium sulfate over slant. Development of pink color within 15 min is a positive test, indicating presence of pyrazinoic acid formed by pyrazinamidase enzyme.

Steps for isolation and identification of *Yersinia enterocolitica* from faeces and food

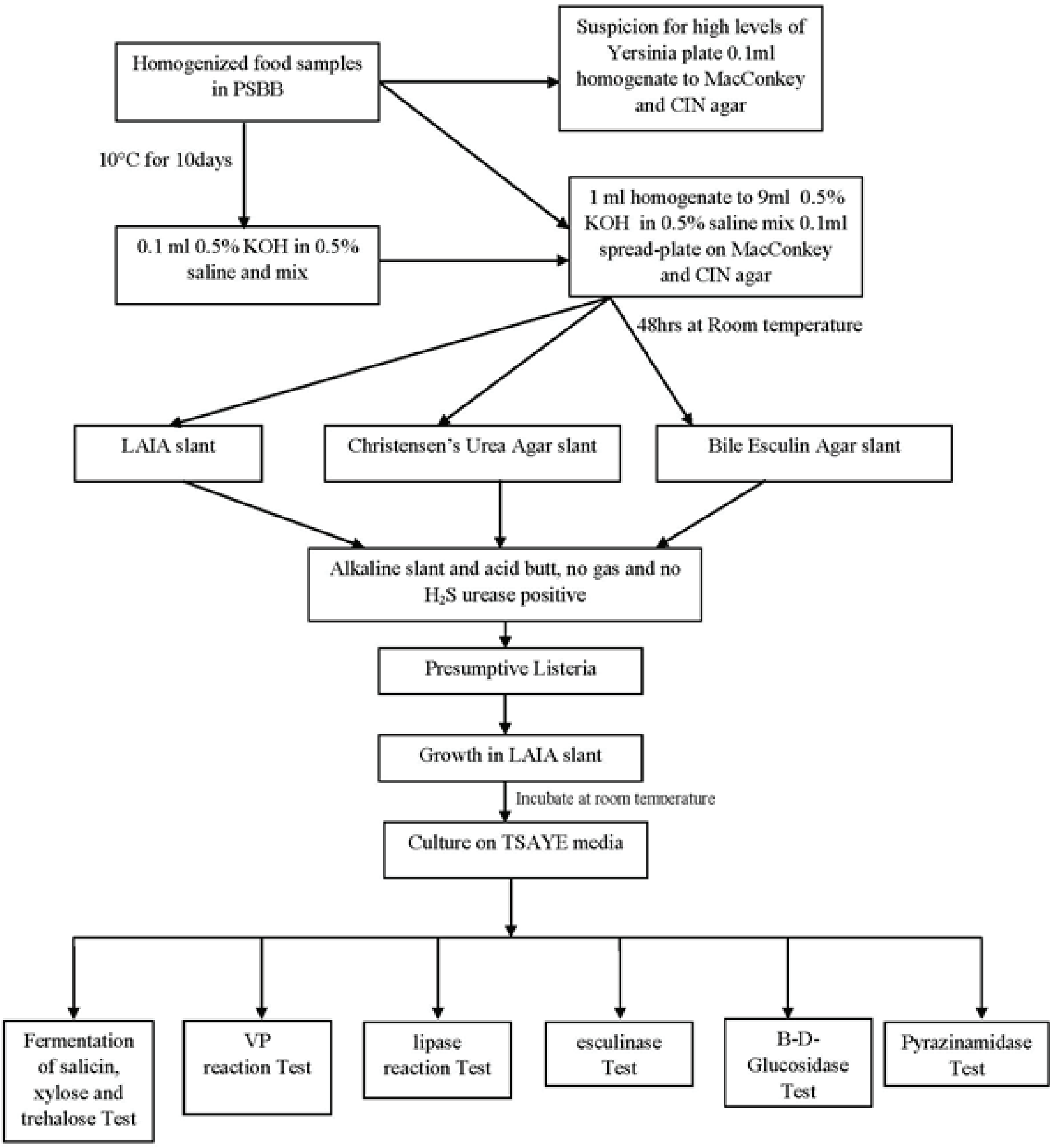

Flow Chart 17: Steps for isolation and identification of *Yersinia enterocolitica* from faeces and food

HEPATITIS A VIRUS (HAV)

Detection of HAV in food samples(fresh vegetables, milk and water samples)

Extraction of viral RNRA shall be carried out using commercial kits following manufacturer's instructions.

Preparation of faecal specimens

Add 1 ml of suitable sample diluents/DMEM to 1.5ml microfuge tube to prepare a 10% suspension or dilution of faecal specimen by the addition of approximately 0.1g of solid faeces (small pea-sized portion) or approximately 100 µl of liquid faeces. Mix thoroughly and centrifuge at 10,000 rpm for 10 min at 4°C. Collect the supernatant carefully in a new tube. Make aliquot accordingly and store at -80°C for further use.

Extraction of viral RNA/DNA for RT-PCR will be done by using commercial DNA/RNA extraction kit.

Procedure: As per manufacturer's protocol.

RT-qPCR for DETECTION of HAV

A. Reverse-Transcription

In the reverse-transcription step, to 10 µL of the extracted RNA will be added 1 µL of 100 µM Random Hexamer primer, 1 µL dNTP mix (10 mM each of the four deoxynucleoside triphosphate stocks), 2.5 µL DEPC-treated water, 4 µL 5 × RT buffer, 0.5 µL Ribolock RNase inhibitor and 1 µL of 200-U/µl RevertAidTM Premium reverse transcriptase (Fermentas, Burlington, ON, Canada) into a 0.5 mL PCR tube on ice. The reaction mixture (20 µL) will be briefly vortexed to ensure total mixing and then centrifuged. The tubes will be incubated at 25°C for 10 min followed by 30 min at 60°C. The reaction will be terminated by heating at 85°C for 5 min. The resulting 20 µL of cDNA will be kept at −20°C until use for qPCR.

B. Quantitative PCR (qPCR)

Hepatitis A virus primers/probes:

HAV68 (F): 5'-TCA CCG CCG TTT GCC TAG-3'

HAV240 (R): 5'-GGA GAG CCC TGG AAG AAA G-3'

HAV150 (P): 5'-FAM-CCT GAA CCT GCA GGA ATT AA-MGBNFQ-3

Briefly, 5 µL out of 20 µL of the cDNA will be mixed with 20 µL of a reaction buffer (containing 12.5 µL of 2 × TaqMan universal PCR master mix (Applied Biosystems), 400 nM sense primer, 400 nM antisense primer, and 250 nM TaqMan probe and PCR grade water to give a 25-µL total reaction mixture. Subsequently, the mixture will be added to a well of a 96-well micro-plate and loaded into the Real time PCR System. Fluorescence data will be collected at the end of annealing step. The thermal cycling protocols used.

Virus	Taq activation	45 cycles of		
		Denaturation	Annealing	Extension
Hepatitis A virus	10 min at 95°C	15 s at 95°C	1 min at 60°C	1 min at 70°C

HEPATITIS E VIRUS (HEV)

Detection of HEV from food & stool samples

A. RT-qPCR amplification.

Primer/Probe	Design and nucleotide sequence	Concentration 1X, µM
**HEV ORF 2/3**		
HEV forward primer	5'– AATAAATCATAAGGTGGTTTCTGGGGTGAC –3'	0.250
HEV reverse primer	5'– AATAAATCATAAGGGGTTGGTTGGATGAA –3'	1.000
HEV probe	5'– MGB-FAM-G*TGATTCTCAGCCCTTCG-NFQ –3'	0.400
**MS2 RNA internal control**		
MS2 forward primer	5'– CCA*TCAAA*GTCGA*GGTGCCTAAAGTG –3'	0.075
MS2 reverse primer	5'– ACGAACGCCATGCGGCTACAGGAAGCTC –3'	0.075
MS2 probe	5'–MGB-AP525-G*CTGTTGGTGGTGTAGAGC-NFQ–3'	0.200

Each 25-µl RT-qPCR reaction will include 12.5 µl of extracted RNA with primers and probes and 5x LibertyTaq One-Step RT-qPCR Master Mix containing heat-labileuracil-N-glycosylase (UNG) and ROX passive reference dye (Thermo Fisher Scientific,Waltham, MA). Amplification and detection will be performed on Real-Time PCR System using ROX reference dye normalization, 100% ramp rates.

Thermal cycling profile: 20 min at 30°C for one cycle, 5 min at 55°C for one cycle, 30 min at 49°C for one cycle, 2 min at 95°C for one cycle. (Or as per kit manufacturers instruction).

Followed by 50 cycles of a three-step cycle (15 sec at 95°C, 30 sec at 56°C, 30 sec at 72°C). This amplification profile was followed by dissociation curve analysis, which consisted of a 15 sec hold time each at 95°, 35°, and 95°C with 100% ramp rates, except for a 5% ramp rate during the final ramp from 35° to 95°C.

Fluorescence data will be collected at the end of annealing step.

The maximum change in normalized fluorescence (ΔRn) values obtained from the sample of 50 healthy control without evidence of HEV infection will be used to determine the mean background ΔRn and to establish a standardized cycle threshold (Ct) of 0.08 ΔRn (mean background ΔRn + 10 SD) for assessment of HEV target.

NOROVIRUS

Detection of Norovirus Genogroup I and II by multiplex real time RT-PCR

From stool samples: The stool samples will be subjected to viral RNA extraction using the QIA amp Viral RNA mini kit (QIAGEN, Hilden, Germany) followed by a multiplex real time RT-PCR using a specific MGB probe for simultaneous detection of both the genogroups. The primer and probe sequences used for the multiplex real time RT-PCR is listed in Table 1.

Table 1: Primers and probes used for one tube multiplex real-time RT-PCR

Genogroup	Primer	Sequences (5'-3')	Location
GI	NV192 (s)	5'-GCYATGTTCCGCTGGATGC	5282–5300
	NV193 (as)	5'-CGTCCTTAGACGCCAT-CATCA	5379–5359
	TM9-MGB probe	5'-VIC-TGGACAGGAGATC-GC-MGB-NFQ	5345–5359
GII	NV107a (s)	5'-AGCCAATGTTCAGATGGATG	5007–5026
	NV107c (s)	5'-AICCIATGTTYAGITGGATG	5007–5026
	NV119 (as)	5'-TCGACGCCATCTTCATTCAC	5100–5081
	TM3A probe	5'-6'FAM-TGGGAGGGCGATCG-CAATCTGGC-NFQ	5048–5070

**s- sense, as- antisense*

Thesingle-tube multiplex real time RT-PCR will be carried out in PCR tubes or plates using a TaqMan® 7700. The reactionwill be performed in 12 µl volumes using the Probe RT-PCR mix containing the Omniscript and Sensiscript reversetranscriptases, 0.2 µM of eachprimer as described in table 1; 80 nM GI TM9 MGB probe;160 nM GII TM3A probe. Two microliters of sample RNA preparation or standard DNA will be added to each reaction.

Thermal cycling for the TaqMan 7700 will be performed asfollows: 30 min at 50°C for reverse transcription, 15 minat 95°C for heat inactivation of the reverse transcriptasesand the initial activation of the HotStar polymerase, 45cycles of 20 sec at 94°C and 30 sec at 60°C. Thefluorescence data were collected at the end of the 60°Cstep. To generate a standard curve 10-fold serial dilutionof plasmid-DNA containing the appropriate GI or GIIsequences will be used.

PARASITES

Meat, fish

Sampling and sample preparation

From a sample weighing 1 kg, take a subsample (100 gm) of beef, pork, or poultry, or 250 gm of fish. Subsamples of most mammalian meat, poultry, or fish require no further preparation. They may be torn or separated into 5 or more pieces to increase the surface area. A 100 gm sample blended in 750 ml saline can improve digestion.

Digestion, sedimentation, and examination

Adjust incubator-shaker or water bath to 37 ± 0.5°C. Prepare digestion fluid in 1500 ml beaker by dissolving 15 gm pepsin in 750 ml saline, add sample, and adjust to pH 2 with concentrated HCl (about 3 ml). Place in an incubator or water bath and stir (about 100 rpm) after equilibration for about 15 min; check and adjust pH again. Cover the beaker with aluminum foil (if using stirrer, pierce hole for stirring rod) and continue incubating until digestion is complete. The time required for digestion will vary but should not exceed 24 hrs.

Carefully pour beaker contents through sieve into tray. Rinse remains with 250 ml saline and add to digest. Examine rinsed contents of sieve and record results. Larger parasites will remain on sieve.

Carefully transfer the contents of the tray to a centrifuge tube (50 ml capacity) and centrifuge at (2000×g for 30 min). The sediment will be mixed and examined for detection of parasitic eggs, cysts and larva.

Vegetable preparation

The vegetable samples will be transported to laboratory in plastic bags. They can be immersed immediately in tap water inside a sink/large container and left approximately 6-7 min for sedimentation of mud and dust. Each vegetable sample can be eluted by vigorous agitation of each specimen for 30 min in 1 L of sterile phosphate-buffered saline (pH 7.4), to which 50 ml of 0.01% Tween 80 to be added. The agitation can be carried out in a shaking water bath. The eluent can then be filtered through gauze and then dispensed into clean centrifuge tubes and centrifuged at 2000×g for 30 min. The supernatant to be discarded into disinfectant jar, and the pellet will be washed of Tween 80 by centrifugation (2000×g for 30 min) with sterile phosphate-buffered saline.

The precipitate will be mixed and examined for detection of parasitic eggs, cysts and larva:

Simple smear: a drop of the sediment will be applied on the center of a clean grease-free slide. A clean cover slip will be placed gently. The preparation will be examined under a light microscope using ×10 and ×40 objectives.

Iodine smear: a drop of the sediment will be mixed with a drop of Lugol's Iodine solution and examined as in simple smear.

Simple and iodine smears will be used for detection of parasitic eggs, cysts and larva. The process will be systematically repeated until the mixture in each test tube is exhausted.

Eggs, cysts and oocysts of parasites found under the light microscope will be identified

Staining of sediment smear can be performed by Modified Zeihl–Neelsenand modified trichrome to detect protozoal parasitic oocysts and spores of Microsporidium spp.

Each parasite's eggs, cysts or oocysts present in each sample will be enumerated and densities of each species will be expressed as

"many" (>three oocytes per high-power field; >20 eggs per low-power field);

“moderate” (two oocytes per high-power field; 10–19 eggs per-low power field);

“few” (one oocyte per high-power field; three to nine eggs per low-power field);

“rare” (two to five cysts and <two eggs per 100µ of sediment).

For simplification, numerical values were assigned to each density: many, 4; moderate, 3; few, 2; rare, 1; and none, 0.

6. ANTIBIOTIC SUSCEPTIBILITY TESTING

Disc diffusion testing: Basic Procedure
Inoculum: Isolated colonies of each type of organism that may play a pathogenic role should be selected from primary agar plates.

Preparation of inoculum
Direct colony suspension method – Prepare a saline suspension of the isolate from an overnight-incubated agar plate (use a non-selective medium, such as blood agar) to obtain 0.5 McFarland turbidity (1.5 x 108 cfu/ ml of E. coli ATCC25922).
Growth method – With a sterile straight wire touch the top of each of four to five colonies of the same morphological type, and inoculate MHB or any suitable broth. Incubate tube at 35ºC till turbidity of 0.5 McFarland tube or more is achieved. Then, with sterile normal saline adjust turbidity to exactly 0.5 McFarland.

Inoculating test plates - MHA plate should be inoculated within 15 min after theinoculum has been adjusted. A sterile cotton swab is dipped into the suspension, rotated several times, and gently pressed onto the inside wall of the tube above the fluid level to remove excess inoculum from the swab. The swab will then be streaked over the entire surface of the agar plate three times, with the plate rotated approximately 60°each time to ensure even distribution of the inoculum. A final sweep of the swab will be made around the agar rim. The lid may be left ajar for 3 to 5 min but no longer than 15 min to allow any excess surface moisture to be absorbed before the drug-impregnated discs are applied.

Application of antimicrobial discs to MHA plate – Ideally, this should be done within 15 min of inoculation of plates. The selected antimicrobial discs will be dispensed evenly onto the agar plate with the help of a forceps/ sterile needle/surgical blade. Flame the tips of the applicator intermittently. Each disc must be pressed down to ensure complete contact with the agar surface.

1. Ordinarily no more than 12 discs are applied on a 150 mm plate or 5 discs on a 100 mm plate, keeping at least a distance of 24 mm between discs. Dispensing too near to the edge of the plate should be avoided. Because some of the drugs diffuse instantaneously, a disc should not be relocated once it has come in contact with the agar surface.
2. It is advisable to place discs that give predictably small zones like aminoglycosides, next to those discs that give larger zones like cephalosporins.
3. Disc containers should be removed from the refrigerator or freezer one to two hrsbefore use, so they may equilibrate to room temperature before opening. This procedure minimizes the amount of condensation that occurs when warm air contacts cold disks.
4. Only those discs within the manufacturer’s expiration date stated on the label will be used.

Incubation. No longer than 15 min after the discs are applied, the plates will be inverted and incubated at 35º ± 2ºC in ambient air.

Interpretation and reporting of results – Each plate will be examined after overnight incubation (16-18 hrs), for confluent growth and circular zones of inhibition. The diameters of the zones of complete inhibition, including the diameter of the disc, will be measured to the nearest whole millimeter with callipers or a ruler. With unsupplemented MHA, the measuring device is held on the back of the inverted petri dish, which is illuminated with reflected light located a few inches above a black, nonreflecting background. Zone margin should be considered the area showing no obvious visible growth detectable with the unaided eye. Faint growth of tiny colonies visible only by lens should be ignored. Zone sizes should be measured from the upper inoculated surface of opaque media like MHA with added blood, illuminated with reflected light, with the cover removed. In case of presence of discrete colonies within the clear zone of inhibition, repeat test with a subculture of a single colony/ pure culture from the primary culture plate. With trimethoprim, the sulfonamides, and combinations of the two agents, antagonists in the medium may allow some minimal growth; therefore, the zone diameter should be measured at the obvious margin, and slight growth (20% or less of the lawn of growth) should be disregarded.

The sizes of the zones of inhibition are interpreted by referring to CLSI guidelines and the organisms are reported as either susceptible, intermediate, or resistant to the agents that have been tested. (Refer Tables S1-S3). Use E. coliATCC25922, P. aeruginosa ATCC27853, S. aureus ATCC25923) for quality control with known susceptibility standards (Table S4).

Preparation of 0.5 McFarland Standard (Bailey and Scott, 1994)

0.05 ml of 1.175% (w/v) solution of barium chloride dehydrate (Bacl 2 2h2O) (HiMedia Laboratories Limited, Mumbai, India) to be added slowly and with constant agitation to 9.95ml of 1% sulphuric acid (H2SO4) Merck (India) Ltd, Mumbai) to make a total volume of10 ml in a 15 ml screw capped test tube. The tube is to be stored in dark at room temperature.

7. FOODBORNE DISEASE OUTBREAK INVESTIGATION

Outbreak investigation will be done with the following objectives:

- Identification of specific risk factors related to the host, the agent and the environment
- Identification of factors that contributed to the contamination, growth, survival and dissemination of the suspected agent
- Prevention of future outbreaks and strengthening of food safety policies
- Acquisition of epidemiological data for risk assessment of foodborne pathogens

Investigation of a foodborne disease outbreak will include

- Epidemiological
- Environmental and Food investigation
- Laboratory Investigation(Microbiological)

A. Epidemiological:

Investigation of a potential outbreak will starts with the assessment of all available information. This assessment will include with

- Checking the validity of the information
- Obtaining reports of applicable laboratory tests that have been performed
- Identifying cases and obtaining information about them
- Ensuring the collection of appropriate clinical specimens and food samples.

Investigation of food establishments

During a foodborne disease outbreak, investigation of a food establishment will be done by interviewing managers; employees, reviewing hygiene and illness of the employees, including, specimens for analysis, food and environmental sampling, assessment of the water system and supply.

Investigation of a suspect food

The complete processing and preparation history will be reviewed, including source and ingredient, persons who handled the specific foods, the procedures and equipment used, potential source of contamination, and time and temperature conditions to which foods were exposed.

B. Food sampling

Food samples collection and testing will include ingredients used to prepare implicated foods, leftover foods from a suspect meal/foods from a menu that has been implicated epidemiologically, foods known to be associated with the pathogen in question.

Environmental samples

Samples will be taken from work surfaces, food contact surfaces of equipment, containers, and other surfaces such as refrigerators, door handles, etc. Environmental samples may also include clinical specimens (such as faecal specimens, nasal swabs) from food workers and water used for food processing.

Food-handlers

Stool specimens or rectal swabs, nasal, skin will be collected from food-handlers for laboratory analysis to identify potential carriers or sources of contamination.

B. Laboratory investigations will be done as per protocol

8. QUALITY CONTROL

A. Internal quality control (IQC)

The aim of a quality control program would be to monitor the following:

- Performance of the media and reagents used in the test.
- The precision (repeatability) and accuracy of the susceptibility test procedure
- The performance of persons
- All the participating laboratories would test all media, discs and reagents to be used for the study.
- Sterility: Each batch of medium should be tested for sterility. A plate would be selected at random and incubated at 37°C for 24-48 hrs. The media should be sterile before inoculation. Presence of surface or subsurface colonies warrants discarding of the media.
- Growth: The ability of the medium to support the growth is determined by inoculating the medium with a typical stock culture isolate. Diluted inoculum is used for this purpose. Appropriate growth is observed and recorded after incubation of the medium.
- Biochemical reactions: Media used for biochemical reactions are tested with strains, which give both positive and negative reactions.
- Negative controls (non-spiked autoclaved distilled water) and positive controls will be incorporated with each set of test samples and subjected to RNA extraction and PCR assays.

B. External quality assessment (EQA)

An EQA ensures approved centre staff members are making proper identification that are consistent with the qualification learning/activity outcomes and assessment criteria. For this purpose, ICMR-National Institute of cholera and Enteric Diseases (NICED), Kolkata will send 5 unknown bacterial strains once in year, following all the biosafety measures. Each participating center has to identify and send the results in a format that will be communicated along with the strains.

9. DATA RECORDING AND DOCUMENTATION

The results shall be recorded on the CRF and logbook. An online or offline mobile based application may be used rapid collection of data during routine surveillance and foodborne outbreak surveillance.

In the ICMR-FoodNet, transfer data to the computer and analytical tool/software.

10. REPORTS AND SENDING OF STRAINS/DNA/RNA

The results from all the centers shall be submitted to RMRC, Dibrugarh on a monthly basis.

The report from all the NE states shall be compiled at ICMR-RMRC, Dibrugarh and sent to ICMR, Delhi for analysis on a quarterly basis.

Bacterial strains and DNA/RNA extracted from samples shall be shared with ICMR-RMRC, Dibrugarh on a monthly basis.

ANNEXURE-1

Tests for identification of Salmonella & Shigella
Inoculation and Interpretation of Simmons Citrate Agar

Procedure
A small amount of growth is harvested with a sterile (1 µl) loop.

Lightly inoculate the surface of the agar slant.

Do not use a heavy inoculum.

Tubes are incubated under aerobic conditions at 36°C (+/- 1°C) with caps loosened.

Tubes should be examined and results recorded at 24 hrs, 48 hrs, and 3-5 days.

Interpretation/Results/Reporting
Positive - intense blue color (initially the color change may only occur on the agar slant)

Negative - agar remains green

Quality Control
Quality control testing is performed with each new lot and shipment of media.

Prior to use, the media are tested for sterility: uninoculated media should have no growth following 48 hrs of incubation at 36°C (+/- 1°C). Each new lot / shipment of media must also produce expected reactions with QC organisms. The following reactions must be observed:

Positive: Enterobacter aerogenes

Negative: Escherichia coli

Reference: WHO GFN Laboratory Protocol: "Biochemical Identification of Salmonella and Shigella, Using an Abbreviated Panel of Tests" – version 002; October 2015

Inoculation and Interpretation of Lysine Iron (LIA) Agar

Procedure
A small amount of growth is harvested with a sterile inoculating needle.

Lightly inoculate the surface of the agar slant.

Make a single stab into the butt of the tube.

Tubes are incubated under aerobic conditions at 36°C (+/- 1°C) with caps loosened.

Tubes should be examined and results recorded at 24 hrs, 48 hrs, and 5-7 days (unless H2S production occurs sooner).

Interpretation/Results/Reporting

A. H2S production:
Positive - black color along the streak or throughout the medium

Negative - no black color

B. Lysine Decarboxylase (LDC):
Decarboxylation of lysine is detected in the butt of the tube.

LDC positive organisms will turn the agar in the butt of the tube purple.

LDC negative organisms will turn the agar in the butt of the tube yellow.

C. Lysine Deamination:
Lysine Deamination is detected on the agar slant.

Lysine deaminase positive organisms will turn the agar slant red.

Lysine deaminase negative organisms will turn the agar slant purple

Quality Control
Quality control testing is performed with each new lot and shipment of media.

Prior to use, the media are tested for sterility: uninoculate media should show no growth following 48 hrs of incubation at 36°C (+/- 1°C). Each new lot / shipment of media must also produce expected reactions with QC organisms. The following reactions must be observed.

Bacteria	Slant	Butt	H2S
Proteus mirabilis	Red	Yellow	-
Salmonella Typhimurium	Purple	Purple	+
Shigella flexneri	Purple	Yellow	-

Reference: WHO GFN Laboratory Protocol: "Biochemical Identification of Salmonella and Shigella, Using an Abbreviated Panel of Tests" – version 002; October 2015

Inoculation and Interpretation of Motility-Indol-Ornithine Agar (MIO Agar)

Procedure
A small amount of growth is harvested with an inoculating needle.

Make a single stab into the tube of MIO agar. The stab should be made straight into the agar and stop approximately 1 cm from the bottom of the tube.

Do not make multiple stabs into the agar and do not twist the needle into the media.

Tubes are incubated under aerobic conditions at 36°C (+/- 1°C) with caps loosened.

Tubes should be examined and results recorded following overnight (18-24 hrs) incubation.

Interpretation/Results/Reporting

Motility
Positive: Visible growth extending away from the stab line. Typically the agar will become visibly turbid.

Negative: Growth only along the stab line. The agar remains clear. Isolates which only produce small tufts of growth along the stab line (similar to bristles on a brush) are considered non-motile.

Ornithine Decarboxylase
Positive: The agar in the middle of the tube turns a light, purple colour. These tubes are distinctly purple; however, they will be a lighter shade of purple than their uninoculated counterparts.

Negative: The agar in the middle of the tube turns yellow. Only the colour of the agar in the middle of the tube should be noted. Oxidation may cause the agar on the surface of the tube to turn purple this is not significant.

Quality Control
Quality control testing is performed with each new lot and shipment of media.

Prior to use, the media are tested for sterility: Uninoculated media should show no growth following 48 hrs of incubation at 36°C (+/- 1°C). Each new lot/shipment of media must also produce expected reactions with QC organisms. The following reactions must be observed:

Motile/Ornithine Positive/Indol Positive: E. coli.

Non-motile/Ornithine Negativ /indol Negative: Shigella flexneri

Reference: WHO GFN Laboratory Protocol: "Biochemical Identification of Salmonella and Shigella, Using an Abbreviated Panel of Tests"-version 002; October 2015

Inoculation and Interpretation of Triple Sugar Iron (TSI) Agar

Procedure
A small amount of growth is harvested with a sterile inoculating needle.

Lightly inoculate the surface of the agar slant.

Make a single stab into the butt of the tube.

Tubes are incubated under aerobic conditions at 36°C (+/- 1°C) with caps loosened.

Tubes should be examined and results recorded at 24 hrs, 48 hrs, and 5-7 days (unless H2S production occurs sooner).

Interpretation:

A. Carbohydrate fermentation:
Alkaline slant/alkaline butt- no sugars fermented

Alkaline slant/acid butt- only glucose fermented

Acid slant/acid butt- glucose fermented along with lactose and/or sucrose

B. Gas production:
Positive- gas bubbles in agar or splitting of agar

Negative- no bubbles or splitting of agar

C. H2S production:
Positive - black colour along the streak or throughout the medium

Negative - no black colour

Quality Control
Quality control testing is performed with each new lot and shipment of media.

Prior to use, the media are tested for sterility: uninoculated media should show no growth following 48 hrs of incubation at 36°C (+/- 1°C). Each new lot/shipment of media must also produce expected reactions with QC organisms. The following reactions must be observed:

Bacteria	Slant	Butt	Gas
Enterobacter aerogenes (e.g. strain CDC 659-66)	Acid	Acid	+
Citrobacter freundii	Alkaline	Acid	+
Pseudomonas aeruginosa (e.g. ATCC 27853)	Alkaline	Alkaline	-

Recording Results:

TSI results are recorded using the following notations:
Acidification is indicated with the capital letter "A"

Alkalization is indicated with the capital letter "K"

Gas production is indicated with a lower case letter "g"

Hydrogen sulphide production is indicated as follows:

"Tr" = Trace amounts of hydrogen sulphide

"+" = Small to moderate amount of hydrogen sulphide

"+++" = Large amounts of hydrogen sulphide

The fermentation reactions on the slant and butt are recorded. The reactions are separated by a diagonal line. The gas production is noted in the subscript and H2S production is noted in subscript

Reference: WHO GFN Laboratory Protocol: "Biochemical Identification of Salmonella and Shigella, Using an Abbreviated Panel of Tests" – version 002; October 2015

ANNEXURE-2

Tests for identification of Campylobacter

The media and reagents are available from companies like Oxoid, Merck and Difco. Also the media should be prepared according to the manufacturer's description if it differs from the description given here.

Preston Broth

Composition

Lab-Lemco meat extract	10.0 g
Peptone	10.0 g
Sodium chloride	5.0 g
Sodium pyruvate	0.25 g
Sodium metabisulphite	0.25 g
Ferrous sulphate	0.25 g
Water	1000 ml
Preston selective supplement	
Polymyxin B	5000 i.u.
Trimethroprim	10.0 mg
Rifampicin	10.0 mg
Cycloheximide (instead: Amphotericine-B)	100.0 mg
Lysed horse blood	50 ml

Preparation:
Dissolve the dehydrated medium in the water by heating if necessary. Transfer into a bottle and autoclave at 121°C for 15 min. Allow the media to cool to below 50°C before adding the selective (and growth) supplements and the lysed horse blood as appropriate.

CCD (Charcoal cefoperazone deoxycholate) agar

Composition

Campylobacter Blood-Free Selective Agar Base	45.5 g
Meat extract	10.0 g
Enzymatic digest of animal tissues	10.0 g
Sodium chloride	5.0 g
Charcoal	4.0 g
Casein hydrolysate	3.0 g
Sodium deoxycholate	1.0 g
Ferrous sulphate	0.25 g
Sodium pyruvate	0.25 g
Agar	18.0 g
Water	1000 ml

2 vials of CCDA Selective Supplement consisting of

Cefoperazone	32 mg
Amphotericin-B	10 mg

Preparation

Dissolve Campylobacter Agar Base in water by heating if necessary. Autoclave at 121oC for 15 min. Add to each of the 2 vials 2 ml of sterile water. Dissolve gently. Add the selective supplement to the 50oC warm Campylobacter Agar Base. Pour plates with about 15-20ml melted medium in each petridish.

Columbia-agar

Composition

Columbia agar base	45 g
Water	1000 ml

Preparation

Dissolve the Agar Base in water, and let it stand for 15 min. Boil the solution for 15 min and adjust pH~7.1-7.5. The medium is poured into 1000 ml flasks and autoclaved at 121oC for 15 min.

Gram-staining

Composition & Preparation

Crystal violet

Crystal violet	2.0
Ethanol 95% (vol/vol)	20.0 ml
Ammonium oxalate	0.8 g
Distilled water	80.0 ml

The crystal violet is first dissolved in the ethanol, then the ammonium oxalate is dissolved in the distilled water. The two solutions are added together. To aid the dissolving process, both mixtures are agitated in a bath of hot water.

Gram's iodine

Iodine crystals	1.0 g
Potassium iodide	2.0 g
Distilled water	200 ml

The iodine crystals and the potassium iodine are ground together in a mortar and the distilled water is added slowly. If necessary the mixture can be agitated in a bath of hot water to aid dissolution.

Decolourizer

Ethanol 95% (vol/vol)

Carbol fuchsine (counterstain)

Concentrated carbol fuchsine	10.0 ml
Distilled water	90.0 ml

10% (wt/vol) Indoxylacetate solution

Composition & Preparation

Indoxylacetat (C10H9NO2)	10 g
Acetone (C3H6O)	90 ml

Dissolve the chemical in acetone. Stored at +4oC in a dark bottle.

Oxidase solution

Composition & Preparation

L(+)-Ascorbicacid	0.03 g
N,N,N´,N´- Tetramethyl-p-Phenylendiamine Dihydrochloride ($C_{10}H_{16}N_2 \cdot 2HCl$)	0.03 g
Sterile water	30 ml

Dissolve the chemicals in water, and store the solution in a dark bottle at +5 oC for 3 weeks.

3.5% Ninhydrin solution

Composition & Preparation

Ninhydrin ($C_9H_6O_4$)	3.5 g
Acetone (C_3H_6O)	50ml
Butanol ($C_4H_{10}O$)	50ml

Dissolve the chemical in the solutions. Stored at + 5°C in dark bottles of 20 ml.

1% Hippurate solution

Composition & Preparation

Natriumhippurat ($C_9H_8NNaO_3$)	1g
PBS	99ml

Dissolve the chemical with the solutions. Stored at -20oC in tubes of 15 ml.

Test for catalase
Put a colony at a small spot on a slide. Put one drop of 3%-H_2O_2 on the spot with the bacterial material. Examine immediately for evolution of gas, which indicates catalase activity.

Test for oxidase
Transfer one colony to a filter paper. Soak the filter in an oxidase solution. Appearance of a blue color within 10 sec indicates a positive result.

Test for hippurate hydrolysis
Suspend a loopful of a growth from an 18-24 hrs Columbia agar plate containing 5% cattle blood culture in 400 µl of a 1%-hippurate solution. Incubate at 37 for 2 hrs.Then slowly add 200 µl 3.5%-ninhydrin solution to the side of the tube to form an overlay. Reincubate at 37°C for 10 min, and read the reaction.

Positive reaction: dark purple/blue. Negative reaction: clear or gray.

Hydrolysis of indoxyl acetate
Add 50 µl of a 10% (w/v) solution of indoxyl acetate in acetone to an absorbent paper disc

6 mm in diameter and allow to dry in air. Apply growth from a Campylobacter colony directly to disc and then wet with a drop of sterile distilled water. Appearance of a blue-green color within 5-10 minutes indicates a positive result.

Candle jar

Purpose:
The candle jar creates an atmosphere with reduced oxygen and elevated levels of carbon dioxide.

These conditions enhance the growth of microaerophiles.

Principle:
The flame of the candle within a closed environment will use up a certain percentage of the oxygen. When the available oxygen is reduced and elevated carbon dioxide created by the flame is increased, the flame will be extinguished. The plated medium within this atmosphere will show enhanced growth of certain bacteria. The candle jar will usually be incubated at 42°C.

ANNEXURE-3

Tests for identification of Escherichia coli O157:H7

Kovacs reagent for indole reaction
Composition and preparation

4-Dimethylaminobenzaldehyde	5 g
Hydrochloric acid = 1.18 - 1.19 g/ml	25 ml
2-Methylbutan-2-ol	75 ml

Mix the components.

Tryptone/tryptophane medium for indole reaction
Composition and preparation

Tryptone	10 g
Sodium chloride	5 g
DL-Tryptophane	1 g
Water	1000 ml

Dissolve tryptone and chemicals in the water at 100°C. Adjust pH to 7.5 after sterilisation. Dispense 5 ml of medium into tubes and autoclave at 121°C for 15 min.

Indole test Description

When Kovacs reagent containing amyl alcohol and p-dimethylaminobenzaldehyde is added, indole can be extracted into the amyl alcohol layer by shaking a little. Indole and p-dimethylaminobenzaldehyde produces a red/pink colour.

ANNEXURE-4

Tests for identification of vibrios

Alkaline peptone water

Composition and preparation

Peptone	10g
Sodium Chloride	20g
Water	1000ml

Add the ingredients in 1 litre of distilled water. Mix well, distribute into final containers and sterilize by autoclaving at 121°C for 15 min.

Thiosulfate-Citrate-Bile salts-Sucrose agar

Composition and preparation

Proteose peptone	10.0 g
Yeast extract	5.0g
Sodium thiosulphate	10.0g
Sodium citrate	10.0g
Bile	8.0g
Sucrose	20.0g
Sodium chloride	10.0g
Ferric citrate	1.0g
Bromo thymol blue	0.04g
Thymol blue	0.04g
Agar	15.0g
Water	1000ml
Final pH (at 25°C)	

Suspend 89.08 grams in 1000 ml distilled water. Heat to boiling to dissolve the medium completely. Cool to 45-50°C. Mix well and pour into sterile Petri plates.

ANNEXURE-5

Tests for identification of Listeria monocytogens

Buffered Listeria Enrichment Broth (BLEB)
Composition and preparation

Media Base

Trypticase soy broth	30 g
Yeast extract	6 g
Monopotassium phosphate (anhydrous)	1.35 g/liter
Disodium phosphate (anhydrous)	9.6 g/liter
Sodium Pyruvate (Sodium salt)	1.11 g/liter
Distilled water	1 liter

Weigh ingredients and dissolve in water. Autoclave 15 min at 121°C.Final pH, 7.3 ± 0.1.

Selective Supplements

Acriflavin HCl	10 mg/liter
Nalidixic acid (sodium salt)	40 mg/liter
Cycloheximide	50 mg/liter

Prepare acriflavin and nalidixic acid supplements as 0.5% (w/v) stock solutions in distilled water. Prepare cycloheximide supplement as 1.0% (w/v) stock solution in 40% (v/v) solution of ethanol in water. Filter-sterilize stock solutions and store at 4°C, protect acriflavin from light.

Aseptically add the 3 selective supplements to enrichment after 4 hrs incubation at 30°C. The final volume per supplement stock solution determined by total volume of BLEB used in enriched sample.

Fraser broth

Composition and preparation

Peptic digest of animal tissue	5.0g
Casein enzymic hydrolysate	5.0g
Yeast extract	5.0g
Meat extract	5.0g
Sodium chloride	20.0g
Disodium hydrogen phosphate.2H2O	12.0g
Potassium dihydrogen phosphate	1.35g
Esculin	1.0g
Lithium chloride	3.0g
Final pH (at 25°C)	7.2±0.2

Suspend 54.92 gm (equivalent weight of dehydrated medium per litre) in 1000 ml distilled water. Heat if necessary to dissolve the medium completely. Sterilize by autoclaving at 15 lbs pressure (121°C) for 15 min. Cool to 45-50°C and aseptically add rehydrated contents of 1 vial of Fraser Selective Supplement and 2 vials of Fraser Supplement to 1000 ml medium for primary enrichment or 1 vial of each to 500 ml medium for secondary enrichment. Mix well and dispense as desired.

PALCAM agar

Composition and preparation

Basal medium

Peptone	23 g
Starch	1 g
NaCl	5 g
Columbia agar	13 g
Mannitol	10 g
Ferric ammonium citrate	0.5 g
Esculin (aesculin)	0.8 g
Dextrose (glucose)	0.5 g
Lithium chloride	15.0 g
Phenol red	0.08 g
Distilled water	1000 ml

Selective agents

Polymyxin B sulfate	10 mg
Acriflavin	5 mg
Ceftazidine	20 mg
Distilled water	2 ml

To make 500 ml of medium, weigh 34.4 gm basal medium powder (all ingredients except the three selective agents) and suspend in 500 ml distilled water. Sterilize by autoclaving at 121°C for 15 min. Dissolve the selective agent supplement mixture in sterile distilled water at 17.5 mg/ml and filter sterilize. Add 1 ml selective agent supplement solution to 500 ml sterile basal medium that has been cooled to 50°C. Mix gently and pour plates. Final pH, 7.2 ± 0.1.

Trypticase Soy Agar with 0.6% Yeast Extract (TSAYE)

Composition and preparation

Trypticase soy agar	40 g
Yeast extract	6 g
Distilled water	1 liter

Weigh ingredients, add water, mix, and autoclave 15 min at 121°C. Final pH, 7.3 ± 0.2.After autoclaving, swirl to disperse molten agar.

Motility Test Medium

Composition and preparation

Beef extract	3 g
Peptone or gelysate	10 g
*NaCl	5 g
Agar	4 g
Distilled water	1 liter

Heat with agitation and boil 1-2 min to dissolve agar. For use with Salmonella, see the instructions listed below. Dispense 8 ml portions into 16 × 150 screw-cap tubes. Autoclave 15 min at 121°C.Final pH, 7.4 ± 0.2.

ANNEXURE-6

Tests for identification of Bacillus cereus

Mannitol-Egg Yolk-Polymyxin (MYP) Agar

Composition & Preparation

Base

Beef extract	1 g
Peptone	10 g
Mannitol	10 g
NaCl	10 g
Phenol red (1% solution in 95% ethanol)	2.5 ml
Agar	15 g
Distilled water	900 ml

Heat with agitation to dissolve agar. Adjust pH so that the value after sterilization is 7.2 ± 0.2. Dispense 225 ml portions to 500 ml Erlenmeyer flask. Autoclave 15 min at 121°C. Cool to 50°C. MYP agar is commercially available from Difco.

Polymyxin B solution, 0.1%

Dissolve 500,000 units polymyxin B sulfate in 50 ml distilled water. Filter-sterilize and store in the dark at 4°C until needed.

Egg yolk emulsion, 50%

Also available from commercial suppliers.

Final medium

To 225 ml melted base add 2.5 ml polymyxin B solution and 12.5 ml egg yolk emulsion. Mix and dispense 18 ml portions to sterile 15 × 100 mm petri dishes. Dry plates at room temperature for 24 hrs before use.

MRVP Broth

Composition& preparation

Buffered peptone	7.0g
Glucose	5.0g
Dipotassium phosphate	5.0g

Dissolve ingredients in water and adjust pH if necessary. Dispense 5 ml portions into 20 × 150 mm tubes. Autoclave 10 min at 121°C.Final pH, 6.5 ± 0.2.

Voges-Proskauer Reagent A: Barritt's reagent A

Alpha-Naphthol, 5%	50 ml
Absolute Ethanol	1000ml

Voges-Proskauer Reagent A: Barritt's reagent B

Potassium Hydroxide	400 gm
Deionized Water	1000ml

Phenol Red Glucose Broth

Composition & Preparation

Proteose peptone No. 3	10 g
NaCl	5 g
Beef extract (optional)	1 g
Dextrose	5 g
Phenol red	
(7.2 ml of 0.25% solution)	0.018 g
Distilled water	1 liter

Dispense 2.5 ml portions into 13 × 100 mm tubes. Autoclave 10 min at 118°C.Final pH, 7.4 ± 0.2.

Nitrite Detection Reagents

Composition & Preparation

A. Sulfanilic acid reagent

Sulfanilic acid	1 g
5 N acetic acid	125 ml

B. N-(l-naphthyl)ethylenediamine reagent

N-(l-naphthyl)ethylenediamine dihydrochloride	0.25 g
5 N acetic acid	200 ml

C. α-Naphthol reagent

α-Naphthol reagent	1 g
5 N acetic acid	200 ml

To prepare 5 N acetic acid, add 28.75 ml glacial acetic acid to 71.25 ml distilled water.

Store reagents in glass-stoppered brown bottles.

Motility Medium (for Bacillus cereus)

Composition & Preparation

Trypticase	10 g
Yeast extract	2.5 g
Dextrose	5 g
Na2HPO4	2.5 g
Agar	3 g
Distilled water	1 liter

Heat with agitation to dissolve agar. Dispense 100 ml portions to 170 ml bottles. Autoclave 15 min at 121°C.Final pH, 7.4 ± 0.2. Cool to 50°C. Aseptically dispense 2 ml portions to sterile tubes. Store at room temperature 2 days before use.

Nitrate Broth

Composition & Preparation

Beef extract	3 g
Peptone	5 g
KNO3 (nitrite-free)	1 g
Distilled water	1 liter

Dissolve ingredients. Dispense 5 ml portions into tubes. Autoclave 15 min at 121°C.Final pH, 7.0 ± 0.2.

Lysozyme Broth

Composition & Preparation

Prepare nutrient broth as recommended. Dispense 99 ml portions to 170 ml bottles. Autoclave 15 min at 121°C. Cool to room temperature before use.

Lysozyme solution

Dissolve 0.1 g lysozyme in 65 ml sterile 0.0l N HCl. Heat to boiling for 20 min. Dilute to 100 ml with sterile 0.0l N HCl. Alternatively, dissolve 0.1 g lysozyme in 100 ml distilled water. Sterilize by filtration through 0.45 µm membrane. Test for sterility before use. Add 1 ml lysozyme solution to 99 ml nutrient broth. Mix and dispense 2.5 ml portions to sterile 13 × 100 mm tubes.

Tyrosine Agar

Composition & Preparation

Base

Prepare Nutrient agar. Dispense 100 ml portions into 170 ml bottles. Autoclave 15 min at 121°C. Cool to 48°C.

Tyrosine suspension

Suspend 0.5 g L-tyrosine in 10 ml distilled water in 20 × 150 mm culture tube. Mix thoroughly with Vortex mixer. Autoclave 15 min at 121°C.

Final medium

Combine 100 ml base with sterile tyrosine suspension. Mix thoroughly by gently inverting bottle 2 or 3 times. Aseptically dispense 3.5 ml into 13 × 100 mm tubes with frequent mixing. Slant tubes and cool rapidly to prevent separation of tyrosine.

Biochemical tests for Bacillus cereus

1. Acid from glucose: Inoculate 3 ml broth with 2 mm loopful of culture. Incubate tubes anaerobically 24 h at 35°C (GasPak anaerobic jar). Shake tubes vigorously and observe for growth as indicated by increased turbidity and color change from red to yellow, which indicates that acid has been produced anaerobically from glucose.

2. Nitrate reduction: Inoculate 5 ml broth with 3 mm loopful of culture. Incubate tubes 24 hrs at 35°C. To test for nitrite, add 0.25 ml each of nitrite test reagents A and C to each culture. An orange color, which develops within 10 min, indicates that nitrate has been reduced to nitrite.

3. VP test: Inoculate 5 ml medium with 3 mm loopful of culture and incubate tubes 48 ± 2 hrs at 35°C. Test for production of acetylmethyl-carbinol by pipetting 1 ml culture into a tube and adding 0.6 ml alpha-naphthol solution and 0.2 ml 40% potassium hydroxide. Shake, and add a few crystals of creatine. Observe results after holding for 1 h at room temperature. The test is positive if pink or violet color develops.

4. Tyrosine decomposition: Inoculate entire surface of tyrosine agar slant with 3 mm loopful of culture. Incubate slants 48 hrs at 35°C. Observe for clearing of medium near growth, which indicates that tyrosine has been decomposed. Examine negative slants for obvious signs of growth, and incubate for a total of 7 days before considering as negative.

5. Growth in Lysozyme broth. Inoculate 2.5 ml of nutrient broth containing 0.001% lysozyme with 2 mm loopful of culture. Also inoculate 2.5 ml of plain nutrient broth as positive control. Incubate tubes 24 hrs at 35°C. Examine for growth in lysozyme broth and in nutrient broth control. Incubate negative tubes for additional 24 hrs before discarding.

ANNEXURE-7

Tests for identification of Staphylococcus aureus

Baird-Parker Medium, pH 7.0

Composition and preparation

Basal medium

Tryptone	10 g
Beef extract	5 g
Yeast extract	1 g
Sodium pyruvate	10 g
Glycine	12 g
Lithium chloride•6H2O	5 g
Agar	20 g

Autoclave 15 min at 121°C.Final pH, 7.0 ± 0.2. If desired for immediate use, maintain the melted medium at 48-50°C before adding enrichment. Otherwise, store solidified medium at 4 ± 1°C up to 1 month. Melt medium before use.

Enrichment

Bacto EY tellurite enrichment.

Complete medium

Aseptically add 5 ml prewarmed (45-50°C) Bacto EY tellurite enrichment to 95 ml melted base. Mix well (avoiding bubbles) and pour 15-18 ml portions into sterile 15 × 100 mm petri dishes. The medium must be densely opaque. Dry plates before use. Store prepared plates at 20-25°C for up to 5 days.

Reference: Official Methods of Analysis of AOAC International, 15th Edition (1990), p. 429

Trypticase (Tryptic) Soy Agar

Composition and preparation

Trypticase peptone	15 g
Phytone peptone	5 g
NaCl	5 g
Agar	15 g
Distilled water	1 liter

Heat with agitation to dissolve agar. Boil 1 min. Dispense into suitable tubes or flasks. Autoclave 15 min at 121°C. Final pH, 7.3 ± 0.2.

Brain Heart Infusion (BHI) Broth

Calf brain, infusion from	200 g
Beef heart, infusion from	250 g
Proteose peptone (Difco) or	
polypeptone (Bioquest)	10 g
NaCl	5 g
Na2HPO4	2.5 g
Dextrose	2.0 g
Distilled water	1 liter

Dissolve ingredients in distilled water with gentle heat.

Dispense broth into bottles or tubes for storage. Autoclave 15 min at 121°C.Final pH, 7.4 ± 0.2. Commercially available BHI is acceptable.

To prepare brain heart infusion agar, add 15 g agar to 1 liter BHI broth. Heat to dissolve agar before dispensing into bottles or flasks.Autoclave 15 min at 121°C.

ANNEXURE-8

Tests for identification of Yersinia enterocolitica

CIN agar

Composition and preparation

Basal Media

Special peptone	20 g
Yeast extract	2 g
Mannitol	20 g
Pyruvic acid (Na salt)	2 g
NaCl	1 g
$MgSO4•7H2O$ (10 mg/ml)	1 ml
Agar	12 g
Distilled water	756 ml

Irgasan (Ciba-Geigy) solution

May be stored at -20°C up to 4 weeks.

Desoxycholate solution

Sodium desoxycholate	0.5 g
Distilled water	200 ml

Bring to boil with stirring; cool to 50-55°C.

A. Sodium hydroxide, [5 N] 1 ml

B. Neutral red, [3 mg/ml] 10 ml

C. Crystal violet, [0.1 mg/ml] 10 ml

D. Cefsulodin (Abbott Labs), [1.5 mg/ml] 10 ml

E. Novobiocin, [0.25 mg/ml] 10 ml

F. Strontium chloride, [10%; filter-sterilized] 10 ml

May be stored at -70°C. Thaw to room temperature just before use.

Preparation: Add ingredients for basal medium to water and bring to boil with stirring. Cool to about 80°C (10 min in 50°C water bath). Add Irgasan solution and mix well. Cool to 50-55°C. Add solution desoxycholate; solution should remain clear. Add solutions A through E. Slowly add solution F with stirring. Adjust pH to 7.4 with 5 N NaOH. Dispense 15-20 ml into each petri dish. Commercially prepared dehydrated Yersinia selective agar (Difco) with supplements may be substituted. Follow manufacturer's instructions for preparation.

Peptone Sorbitol Bile Broth

Composition and preparation

Na_2HPO_4	8.23 g
$NaH_2PO_4 \cdot H_2O$	1.2 g
Bile salts No. 3	1.5 g
NaCl	5 g
Sorbitol	10 g
Peptone	5 g
Distilled water	1 liter

Dispense 100 ml into Wheaton bottles. Autoclave 15 min at 121°C.Final pH, 7.6 ± 0.2.

Bile Esculin Agar

Composition and preparation

Beef extract	3 g
Peptone	5 g
Esculin	1 g
Oxgall	40 g
Ferric citrate	0.5 g
Agar	15 g
Distilled water	1 liter

Heat with agitation to dissolve. Dispense into tubes, autoclave 15 min at 121°C, and slant until solidified. Final pH, 6.6 ± 0.2.

Christensen's Urea Agar

Composition and preparation

Base

Peptone	1 g
NaCl	5 g
Dextrose	1 g
KH_2PO_4	2 g
Phenol red	
(6 ml of 1:500 solution)	0.012 g
Agar	15 g
Distilled water	900 ml

Dissolve all ingredients except urea in 900 ml water (basal medium).For halophilic Vibrio spp., add extra 15 gm NaCl (final NaCl concentration, 2%).Autoclave 15 min at 121°C. Cool to 50-55°C.

Urea concentrate

Urea	20 g
Distilled water	100 ml

Dissolve urea in 100 ml water.

Filter-sterilize; add aseptically to cooled basal medium. Mix. Final pH, 6.8 ± 0.1. Dispense to sterile tubes or petri dishes. Slant tubes for 2 cm butt and 3 cm slant.

Anaerobic Egg Yolk Agar

Composition and preparation

Agar base

Yeast extract	5 g
Tryptone	5 g
Proteose peptone	20 g
NaCl	5 g
Agar	20 g
Distilled water	1 liter

Autoclave 15 min at 121°C. Adjust pH to 7.0 ± 0.2.

2 Fresh eggs: Treatment of eggs

Wash 2 fresh eggs with stiff brush and drain. Soak eggs in 70% ethanol for 1 h. Crack eggs aseptically. Retain yolks. Drain contents of yolk sacs into sterile stoppered graduate and discard sacs. Add yolk to an equal volume of sterile 0.85% saline. Invert graduate several times to mix. Egg yolk emulsion (50%) is available commercially.

Preparation of medium

To 1 liter melted medium (48-50°C) add 80 ml yolk-saline mixture (available from Difco as Bacto Egg Yolk Enrichment 50%), and mix. Pour plates immediately. After solidification dry 2-3 days at ambient temperature or at 35°C for 24 hrs. Check plates for contamination before use. After drying, plates may be stored for a short period in the refrigerator.

Lysine Arginine Iron Agar

Peptone	5 g
Yeast extract	3 g
Glucose	1 g
L-Lysine	10 g
L-Arginine	10 g
Ferric ammonium citrate	0.5 g
Sodium thiosulfate	0.04 g
Bromcresol purple	0.02 g
Agar	15 g

Adjust pH to 6.8. Heat to boiling and dispense 5 ml into each 13 × 100 mm screw-cap culture tube. Autoclave at 121°C for 12 min. Cool tubes in slanted position. (This medium may also be prepared by supplementing Difco lysine iron agar (LIA) with 10 g L-arginine per liter.)

Pyrazinamidase Agar

Tryptic soy agar	30 g
Yeast extract	3 g
Pyrazine-carboxamide	1 g

0.2 M Tris-maleate, pH 6.0 1 liter

Heat to boiling; dispense 5 ml in 16 × 125 mm tubes. Autoclave at 121°C for 15 min. Cool slanted.

ANNEXURE-9

Long-term cold storage of bacterial strains

Materials

- Cryolabel and a permanent marker
- Log phase liquid bacterial culture (culture grown for about 8–12 hrs)
- Micropipette and sterile tips
- Sterile cryovial with screw-cap
- Sterile glycerol (autoclaved)
- Cryo-box

Procedure

1. Grow the bacterial strains to log phase culture (~8 hrs) in 1. 5 ml of Luria-Bertani (LB) broth or Tryptic Soy broth at 37°C.
2. Using the cryolabel, mark a sterile 1.5 ml cryovial with screw-cap tube with the date and strain number
3. Using a micropipette, add 150 µl of sterile glycerol to the tube.
4. Using a new tip transfer 850 µl of the bacterial culture to the same cryovial.
5. Cap the cryovial and mix well by inverting it several times. It is very important that glycerol should be mixed well the bacterial culture before freezing.
6. Arrange the bacterial strains in a cryobox and store in a -80°C freezer.
7. To recover bacterial strains from the glycerol stock, open the cryovial and use a sterile loop or pipette tip to scrape some of the frozen bacteria from the top. Streak the bacterial strain onto an LB agar or blood agar plate.
8. Place the strains back in the freezer. Do not let the glycerol stock unthaw, as frequent freeze and thaw cycles reduce shelf life of strains. Placing the glycerol stock on ice bath while streaking onto agar plates will prevent it from thawing completely and will improve the storage life. Do not to freeze/thaw your glycerol stock too many times.

Long-term storage of bacterial strains at ambient temperatures

Materials

- Permanent marker
- Sticker labels
- Bacterial culture grown on non-selective medium (LB agar or blood agar)
- Straight wire for inoculation
- Freshly prepared sterile nutrient agar (with 0.8% agar) in sugar tubes
- Sterile rubber bungs for airtight sealing sugar tubes
- Paraffin wax

Procedure

1. Grow the bacterial strains overnight in LB agar or blood agar at 37°C.
2. Using sterile straight wire, pick the bacterial colony and stab-inoculate in nutrient agar (with 0.8% agar) in sugar tubes and mark the date and strain number.
3. Incubate the bacterial strains over night with cotton plugs at 37°C.
4. Stopper the culture tubes with sterile rubber bungs. This will be sealed with molten paraffin wax.
5. Melt the paraffin wax in a wide mouthed container

6. For airtight sealing, dip the strain tubes with to cover the rubber bung
7. Store the strains in a labeled box in dark at room temperature. Check the strains frequently. The level of nutrient agar in the culture tube should not be reduced. Revive the cultures once in six months (steps 1-6).

ANNEXURE-10

List of control strains

Bacillus cereus ATCC 14579 or M15149

Brevibacillus laterosporus ATCC 64

Campylobacter jejuni ATCC 29428

C. coli ATCC 33559

C. lari ATCC 35221

Escherichia coil CCUG 29889

Enteroaggregative Escherichia coli (EAEC) IDH-4832

Enteropathogenic Escherichia coli (EPEC) MICRO-0052

Enteroinvasive Escherichia coli (EIEC) IDH-10275

Escherichia coli O157:H7 ATCC 35150 (EHEC) ATCC 35150

Escherichia coil ATCC 25922 (for AMR testing)

Pseudomonas aeruginosa ATCC 27853 and ATCC 27853

Listeria monocytogens ATCC 35152

Salmonella enterica Typhimurium ATCC 14028

Shigella flexneri ATCC 12022

S. sonnei NK4010

S. boydii NK 2379

Staphylococcus aureus ATCC 25923 or ATCC 43300

Vibrio cholerae O1 Ogawa O395

V. cholerae O1 Inaba N16961

V. cholerae O139 MO10

Vibrio parahaemolyticus ATCC 17802

Yersinia enterocolitica ATCC 23715

ANNEXURE-11

Over view of bacterial culture media and other tests for characterization

ANNEXURE-12

Equipment & Standards

Biosafety Cabinet

Autoclave

Incubator(10°C)

Incubator at 37°C and 41.5°C

CO2 incubator

Centrifuge

PCR Thermocycler

Real-time PCR

Electrophoresis unit

Microwave

UV-transilluminator or gel doc

Water bath (50°C)

Balance

Bunsen burner

Homogenizer

4°C refrigerator

-80°Cfreezer

Pipettes

PFGE

Anaerobic jar

Vacuum pump and filtration assembly (Millipore)

ANNEXURE-13

Abbreviations

RL	Ringers lactate
NA	Nutrient Agar
BPB	Buffered peptone broth
PB	Preston Broth
SFB	Selenite F broth
TCBS	Thiosulfate-citrate-bile-sucrose (tcbs) agar
MAC	Mac Conkey agar
SMAC	Sorbitol-MacConkey agar
PSBB	Peptone Sorbitol Bile Broth
BLEB	Buffered Listeria Enrichment Broth
CCD	CCD (Charcoal cefoperazone deoxycholate
ABA	Anaerobic blood agar
CIN	Cefsulodin-Irgasan-Novobiocin
BPA	Baird Parker Agar
APW	Alkaline peptone water
XLD	Xylose lysine desoxycholate
HE	Hektoen Enteric
FB	Fraser Broth
BA	Blood Agar
RCM	Robertsons cooked meat broth
MYP	Mannitol-Egg Yolk-Polymyxin
TW	Tryptone Water
CA	Columbia agar
NB	Nutrient Broth
CT-SMAC	Cefixime Tellurite Sorbitol MacConkey agar
TSAYE	Trypticase Soy Agar with 0.6% Yeast Extract (TSAYE)
AEYA	Anaerobic egg yolk agar
LVEYA	Liver Veal egg yolk agar
UBEA	Urease, bile esculin agar
TSA	Trypticase (Tryptic) Soy Agar
BHI	Brain heart infusion
TPGY	Trypticase-Peptone-Glucose-Yeast Extract Broth
TSI	Triple Sugar Iron
MIO	Motility-Indol-Ornithine Agar
LIA	Lysine Iron (LIA) Agar
SCA	Simmons Citrate Agar
PALCAM	Polymyxin-acriflavine-LiCl-ceftazidime-aesculin-mannitol agar
SU	Sampling Unit
AU	Analytical Unit

ANNEXURE-14

References

1. WHO GFN Laboratory Protocol: "Biochemical Identification of Salmonella and Shigella, Using an Abbreviated Panel of Tests" – version 002; October 2015

2. WHO network building capacity to detect, control and prevent foodborne and other enteric infections from farm to table" Laboratory Protocol

3. Foodborne disease outbreaks: Guidelines for investigation and control, WHO

4.ICMR, SOP Bacteriology

5. Mackie & McCartney, Practical Medical Microbiology, 14th Edition

6. Standard Operating Procedures, Bacteriology,Antimicrobial Resistance Surveillance and Research Network, ICMR

7. Standard Operating Procedures for isolation and identification of Vibrio cholerae. NICED, Kolkata

8. WHO Global Salm-Surv.PCR for identification of Escherichia coli toxins VT1, VT2 and EAE,2003

9. WHO Global Salm-Surv. Laboratory protocol forMultiplex PCR for differentiation of C. coli and C. jejuni

10. Waleed Khan, Muhammad Irshad, Gauhar Rehman and Anwar Hussain. HAV in fresh vegetables: a hidden health risk in district Mardan, Pakistan. SpringerPlus 2014, 3:675

11. Martínez-Martínez, Mónica, Marta Diez-Valcarce, Nigel Cook, Marta Hernández and David Rodríguez-Lázaro. "Evaluation of Extraction Methods for Efficient Detection of Enteric Viruses in Pork Meat Products." Food Analytical Methods 4, no. 1 (2011): 13-22.

12. Martin-Latil, Sandra, Catherine Hennechart-Collette, Laurent Guillier and Sylvie Perelle. "Duplex Rt-Qpcr for the Detection of Hepatitis E Virus in Water, Using a Process Control." International Journal of Food Microbiology 157, no. 2 (2012): 167-173.

13. Martin-Latil Sandra, Hennechart-Collette Catherine, Guillier Laurent, Perelle Sylvie. "Development of an Extraction Method for Hev Detection in Foodstuffs Containing Raw Pig Liver and Its Implementation for Naturally Contaminated Food." International Journal of Food Microbiology, 2013.

14. Isolation, identification, and characterization of Listeria spp. from various animal origin foods Deepti N. Nayak,1 C. V. Savalia,1 I. H. Kalyani,2 Rajeev Kumar,1 and D. P. Kshirsagar1 Veterinary world

15. Maroua Gdoura-Ben Amor, Mariam Siala,Mariem Zayani, Noël Grosset ,Salma Smaoui, Feriele Messadi-Akrout, Florence Baron, Sophie Jan,Michel Gautier and Radhouane Gdour. Foodstuffs in Tunisia. Frontiers in microbiology

16. Salik Nazki. Isolation, molecular characterization and prevalence of Clostridium perfringens in sheep and goats of Kashmir Himalayas, India. Veterinary world

17. WHO Global Salm-Surv. Laboratory protocol for Isolation of thermotolerant Campylobacter from water, 2003

18. WHO Global Salm-Surv. Laboratory protocol for Isolation of thermotolerant Campylobacter from food, 2003

19. Global Salm-Surv Laboratory protocol Isolation and identification of enterohaemorrhagic Escherichia coli O157:H7 2003

20. Hitchins AD, Jinneman K, Chen Y. Bacteriological Analytical Manual. Chapter 10, Detection of Listeria monocytogenes in Foods and Environmental Samples and enumeration of Listeria monocytogenes in foods

21. Blodgett, R. 2006. Appendix 2. Most Probable Number from Serial Dilutions. In U.S. Food and Drug Administration Bacteriological Analytical Manual Online

22. Haim M. Solomon and Timothy Lilly, Jr. US FDA Bacteriological Analytical Manual Chapter 17. Clostridium botulinum. 2001

23. Stephen D. Weagant (ret.) and Peter FengBacteriological Analytical Manual, Chapter 8. Yersinia enterocolitica.

24. Jeffrey W. Bier (ret.), George J. Jackson (ret.), Ann M. Adams, and Richard A. Rude (ret.) Bacteriological

Analytical Manual. Chapter 19.Parasitic Animals in Foods

25. Abbas BA et al. Molecular Detection of Bacillus cereus Emetic Toxin Gene By Pcr And Determine Its Susceptibility Against Punica Granatum Extracts. Basrah Journal Of Veterinary Research 2012; 11(4):79.
26. Mohammad Bokaeian et al. Molecular Detection of Staphylococcus aureus Enterotoxin A and B Genes in Clinical Samples from Patients Referred to Health Centers in Zahedan City. Research in molecular medicine
27. Hoehne M, Schreier E: Detection of Norovirus genogroup I and II by multiplex real-time RT- PCR using a 3'-minor groove binder-DNA probe; BMC Infect Dis, 2006; 6: 69.
28. Clinical and Laboratory Standards Institute (CLSI). 2021. Performance Standards for Antimicrobial Susceptibility Testing 31st ed. CLSI supplement M100. Wayne, PA: Clinical and Laboratory Standards Institute.
29. Clinical and Laboratory Standards Institute (CLSI). 2015. Methods for antimicrobial dilution and disk susceptibility testing of infrequently isolated or fastidious bacteria, 3rd ed. CLSI guideline M45. Clinical and Laboratory Standards Institute, Wayne, PA

Table. S1. List of antimicrobials for susceptibility testing of foodborne pathogens.

For Salmonella spp. and Shigella spp., aminoglycosides, first- and second-generation cephalosporins, and cephamycins may appear active in vitro but are not effective clinically and should not be reported as susceptible. X indicates the antibiotics to be tested for the corresponding pathogen.

Table S2. Zone diameter (in mm) and MIC breakpoints for Enterobacterales and Campylobacter

sulfamethoxazole (1.25/23.75)						

**for Shigella spp only; # for Salmonella spp; $Modified disk diffusion incubation conditions 42°C for 24 hrs.*

Table S3. Zone diameter (in mm) and MIC breakpoints for non-Enterobacterales

				steria monocytogens								

**Vancomycin MIC µg /ml: ≥2, 4-8 and ≥16 for S, I and R, respectively*

Table S4. Disk diffusion QC ranges for Non-fastidious organisms and antimicrobial agents

	one diameter (in mm) of QC Strain		
		eudomonas aeruginosa	***Staphylococcus aureus***
sulfamethoxazole (1.25/			

For Salmonella spp. and Shigella spp., aminoglycosides, first- and second-generation cephalosporins, and cephamycins may appear active in vitro but are not effective clinically and should not be reported as susceptible. X indicates the antibiotics to be tested for the corresponding pathogen.

Supplement Figures

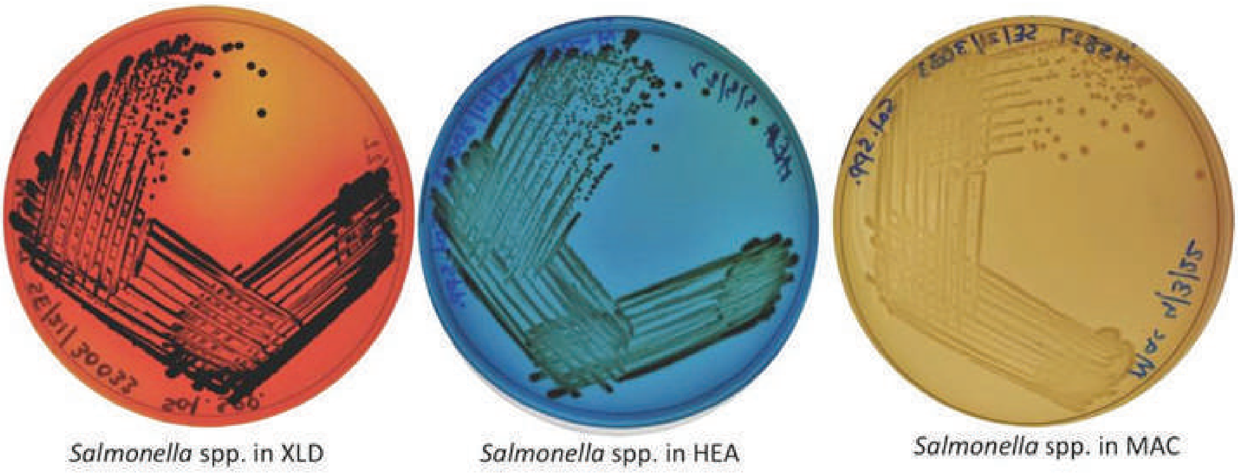

Fig. S1a. *Salmonella enterica* colony morphology in different selective media

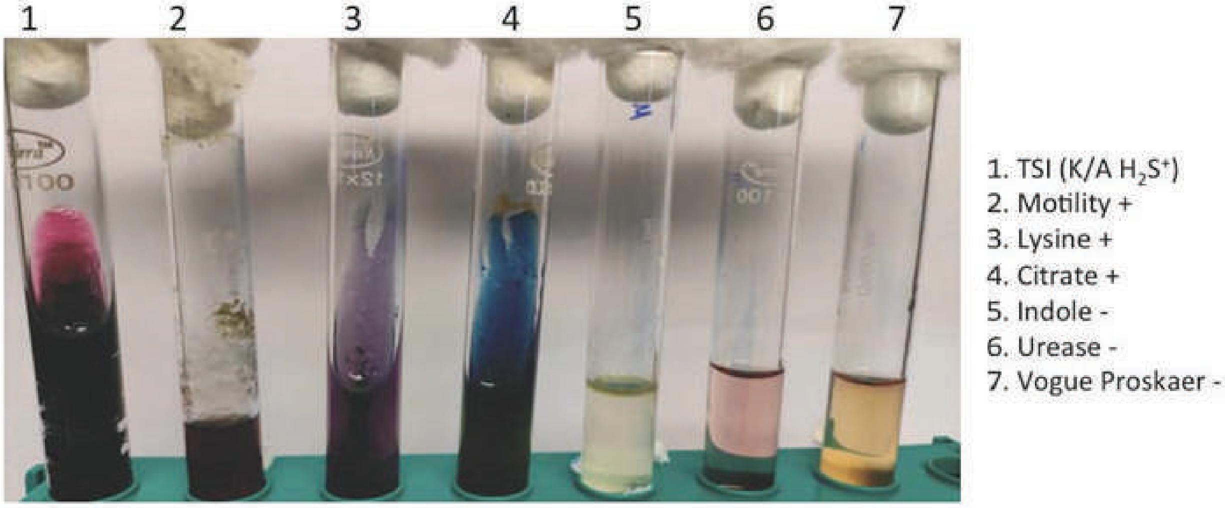

Fig. S1b. *Salmonella enterica* biochemical test results

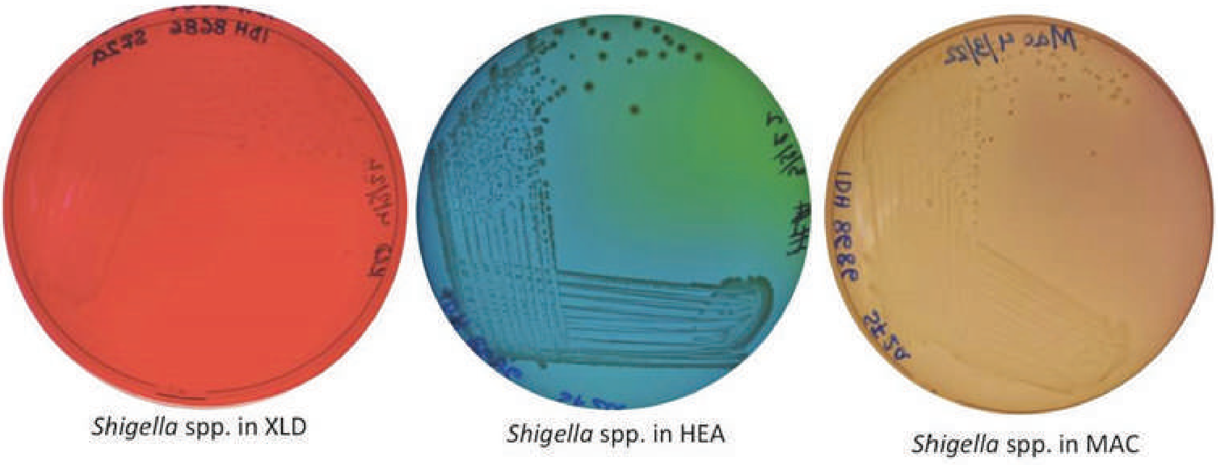

Fig. S2a. *Shigella* spp. colony morphology in different selective media

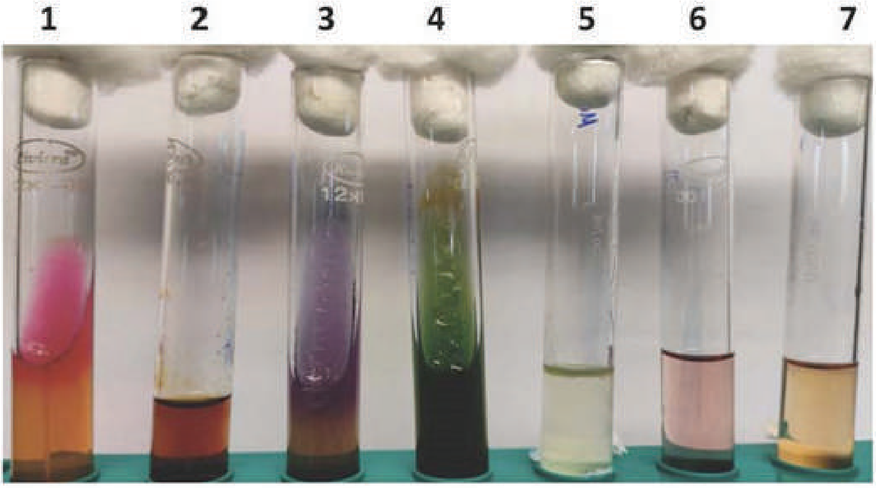

1. TSI K/A
2. Motility -
3. Lysine -
4. Citrate -
5. Indole -
6. Urease -
7. Vogue Proskaer -

Fig. S2b. *Shigella* spp. biochemical test results

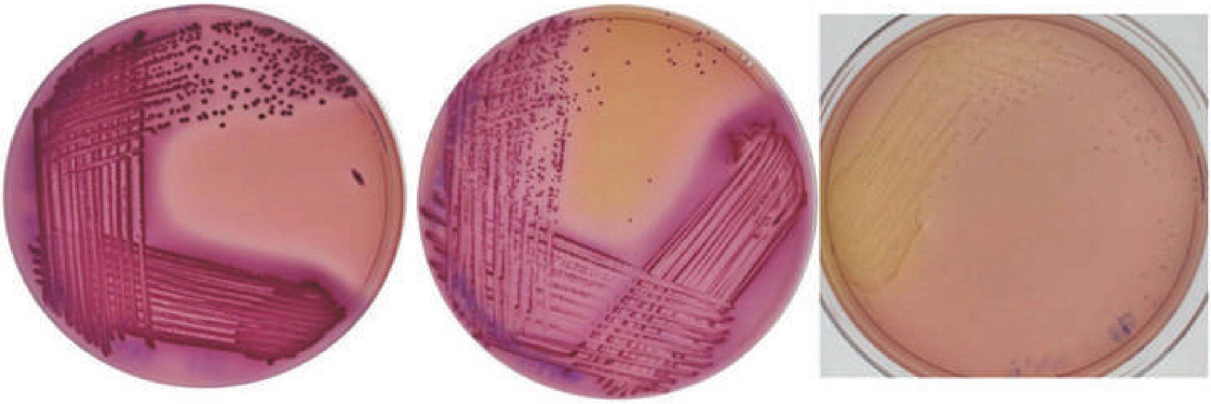

STEC on MAC | STEC on S-MAC | STEC O157:H7 on S-MAC

Fig. S3a. STEC colony morphology in MacConkey and Sorbitol MacConkey media

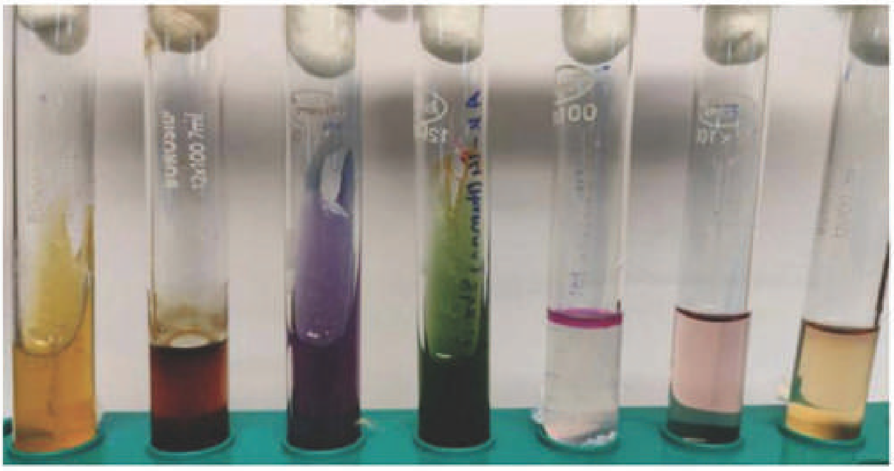

1. TSI A/A
2. Motility +
3. Lysine +
4. Citrate -
5. Indole +
6. Urease -
7. Vogue Proskaer -

Fig. S3b. STEC biochemical test results

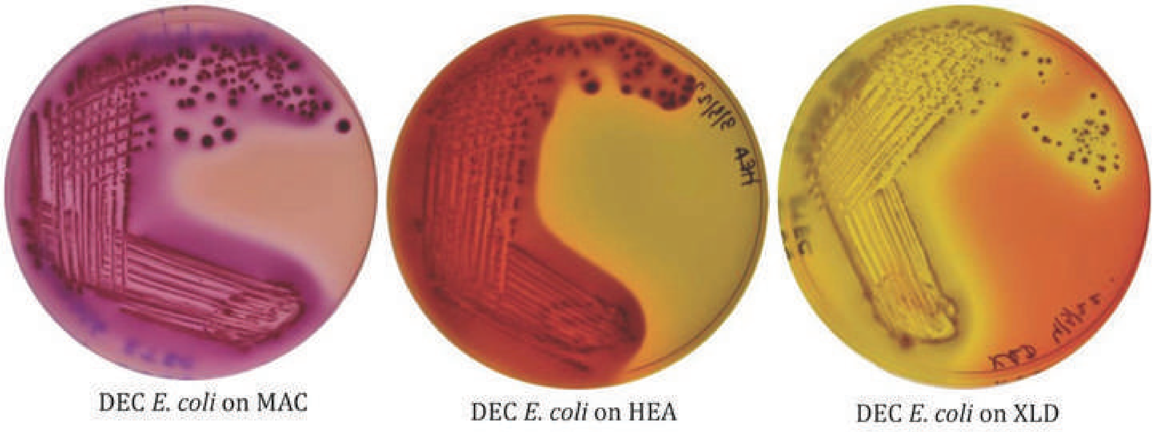

Fig. S4a. DEC colony morphology in different media

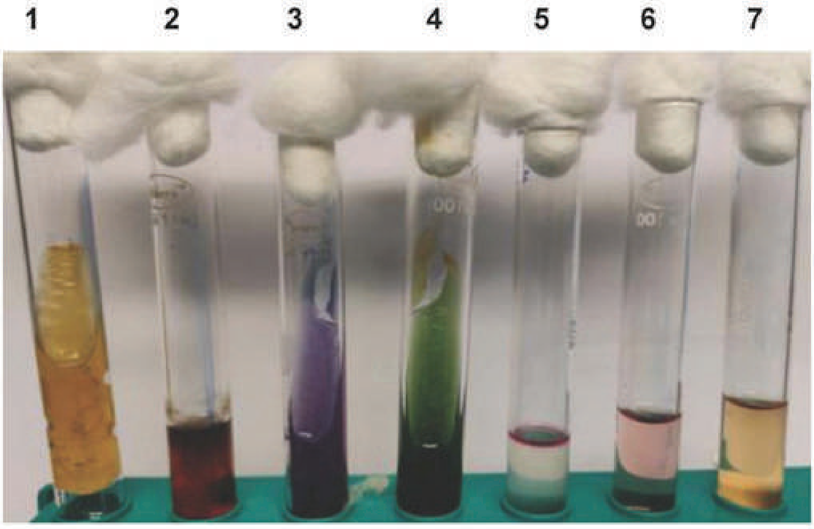

1. TSI A/AG
2. Motility +
3. Lysine +
4. Citrate -
5. Indole +
6. Urease -
7. Vogue Proskaer -

Fig. S4b. DEC biochemical test results

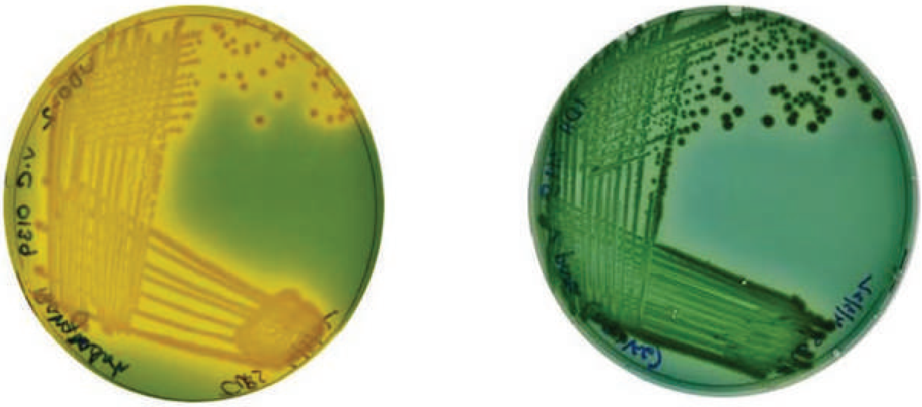

Fig. S5 a. Growth of ***Vibrio cholerae*** (yellow colonies) and ***V. parahaemolyticus*** (green colonies) in Thiosulphate-Citrate Bile salts Sucrose (TCBS) agar.

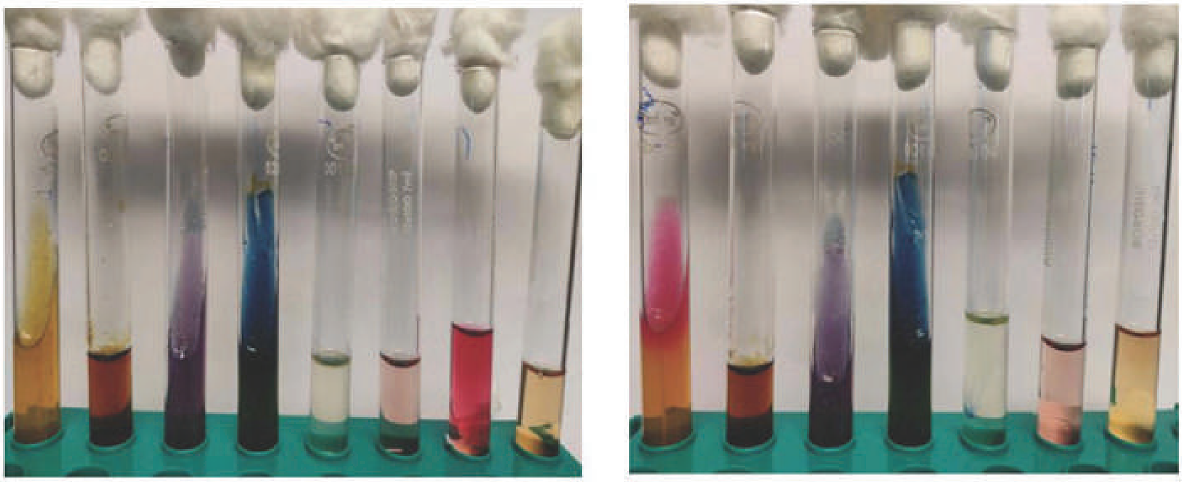

V. cholerae

1. TSI A/A
2. Motility +
3. Lysine +
4. Citrate +
5. Indole +
6. Urease -
7. Vogue Proskaer (+); 8: Vogue Proskaer (-)

V. parahaemolyticus

1. TSI K/A
2. Motility +
3. Lysine +
4. Citrate +
5. Indole +
6. Urease Variable (*trh*-positive strains are +)
7. Vogue Proskaer -

Fig. S5b. *V. cholerae* and *V. parahaemolyticus* biochemical test results

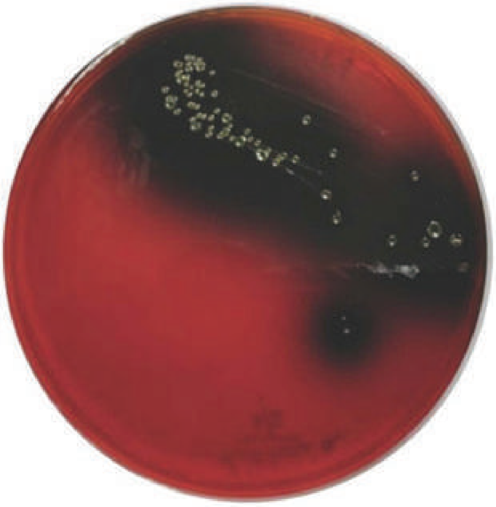

Growth of *Listeria monocytogenes* in PALCAM Agar

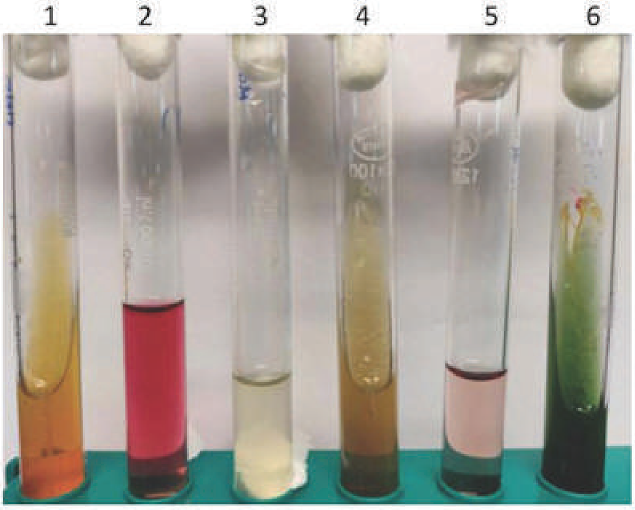

1. TSI A/A
2. Vogue Proskaer +
3. Indole -
4. Lysine -
5. Urease -
6. Citrate -

Fig. S6. Growth and biochemical test results of *Listeria monocytogenes*

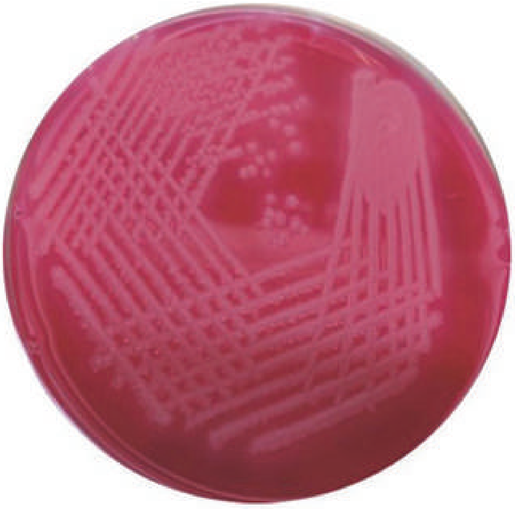

Growth of *Bacillus cereus* in MYP Agar

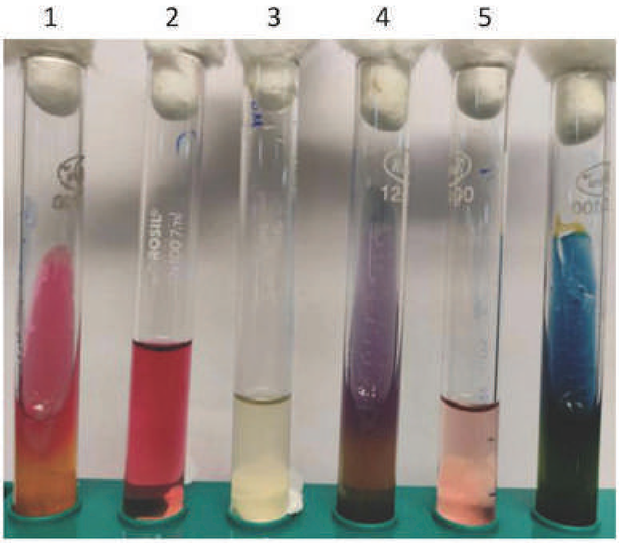

1. TSI K/-
2. Vogue Proskaer +
3. Indole -
4. Lysine -
5. Urease -
6. Citrate +

Fig. S7. Growth and biochemical test results of *Bacillus cereus*

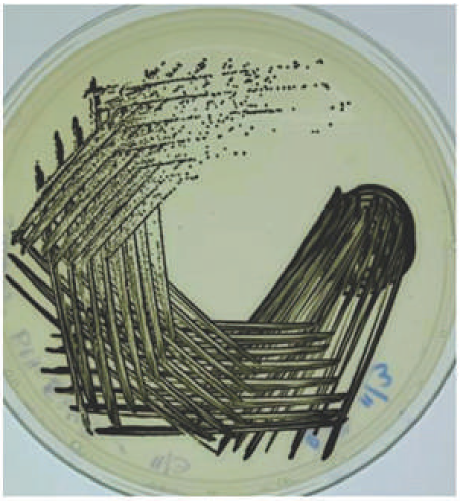

Growth of *Staphylococcus aureus* in Baird Parker Agar

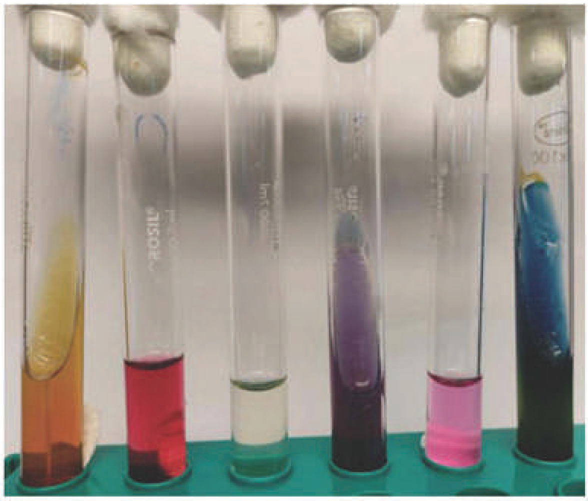

1. TSI -
2. Vogue Proskaer +
3. Indole -
4. Lysine +
5. Urease +
6. Citrate +

Fig. S8. Growth and biochemical test results of *Staphylococcus aureus*

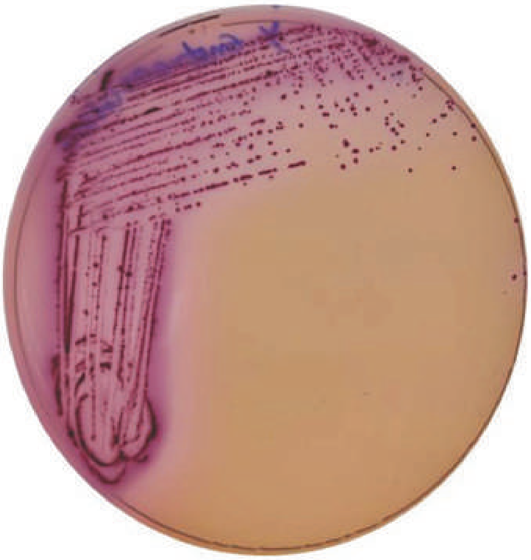

Growth of *Yersinia enterocolitica* in CIN Agar

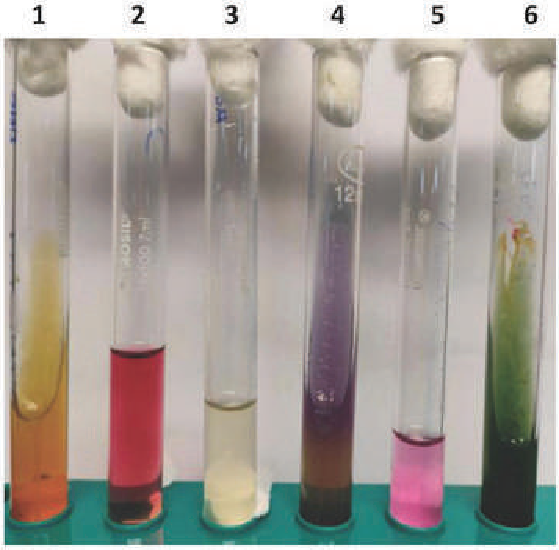

1. TSI A/A
2. Vogue Proskaerv +
3. Indole -
4. Lysine -
5. Urease +
6. Citrate -

Fig. S9. Growth and biochemical test results of *Yersinia enterocolitica*

INDIAN COUNCIL OF MEDICAL RESEARCH

Serving the nation since 1911

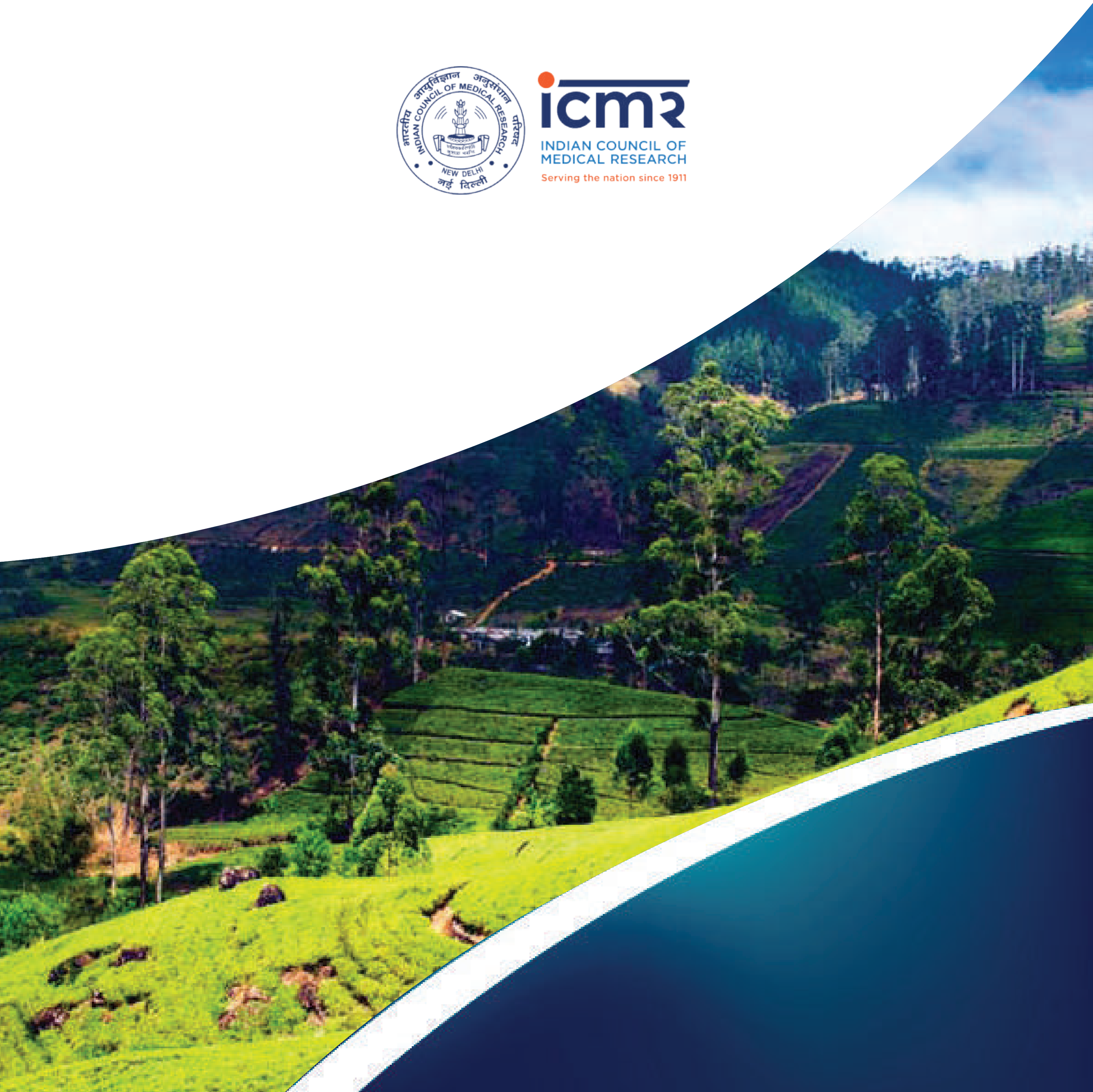

Supplement: Multimedia Appendix 1 [file resprot_v13i1e56469_app1.pdf]
